# Everolimus and *de novo* malignancies after liver transplantation: A nationwide emulated trial using real-world data

**DOI:** 10.1016/j.jhepr.2026.101934

**Published:** 2026-08-07

**Authors:** Ilias Kounis, Tri-Long Nguyen, Christophe Desterke, Nathalie Goutte, Didier Samuel, Daniel Azoulay, Eric Vibert, Faouzi Saliba, Audrey Coilly, Paul Landais, Cyrille Féray

**Affiliations:** 1 INSERM, Université Paris-Saclay, UMR-S 1193, Villejuif, France; 2 AP-HP Hôpital Paul-Brousse, Centre Hépato-Biliaire, Villejuif, France; 3 Université Paris-Saclay, Inserm, Physiopathogénèse et Traitement des Maladies du Foie, Villejuif, France; 4 France FHU Hepatinov, Villejuif, France; 5 Department of Public Health, University of Copenhagen, Denmark; 6 Clinical Research Institute, Montpellier University, France

**Keywords:** mTOR inhibitor, Everolimus, EVR, liver transplantation, immunosuppression, *de novo* malignancies

## Abstract

**Background & Aims:**

Liver transplantation (LT) is associated with an increased risk of *de novo* malignancies (DNMs). Everolimus is an mTOR inhibitor with immunosuppressive and potentially antineoplastic properties. We aimed to evaluate the association between everolimus use and the incidence of DNMs following LT.

**Methods:**

Emulated target trials were conducted using French nationwide administrative health records. The cloning–censoring–weighting approach was applied to estimate the effects of everolimus initiation, everolimus discontinuation, and concomitant tacrolimus use among everolimus users. The primary outcome was the cumulative incidence of DNMs. Secondary outcomes included overall survival and site-specific cancer incidence.

**Results:**

A total of 7,981 patients underwent LT for cirrhosis or hepatocellular carcinoma (44%) between 2009 and 2020. The 10-year cumulative incidence of DNMs was 25.7%. DNMs were less frequent among patients who initiated everolimus within the first 3 years after LT (hazard ratio [HR] 0.84; 95% CI 0.79–0.88). Everolimus discontinuation was associated with an increased risk of DNMs (HR 1.34; 95% CI 1.21–1.45), whereas concomitant tacrolimus use was not significantly associated with DNM risk (HR 1.03; 95% CI 0.83–1.21). Site-specific emulated target trials demonstrated lower incidences of bladder, hematological, lung, prostate, skin, and lymphoid malignancies with everolimus use. The main findings remained robust in sensitivity analyses using alternative definitions of lag times and grace periods.

**Conclusion:**

Sustained everolimus exposure was associated with a lower incidence of *de novo* malignancies after LT. These findings support everolimus-based immunosuppressive strategies incorporating calcineurin inhibitor minimization to potentially reduce long-term oncologic risk.

**Impact and implications:**

This study addressed the lack of randomized evidence regarding the role of everolimus (EVR) in preventing *de novo* malignancies after liver transplantation by leveraging a causal inference framework applied to nationwide data. These findings are important for transplant physicians, researchers, and policymakers, as they suggest that sustained EVR exposure is associated with a reduced risk of post-transplant malignancies without compromising overall survival. In practice, this supports the consideration of EVR as part of immunosuppressive strategies and highlights the potential risks associated with its discontinuation. These results may inform clinical decision-making and future research on optimizing long-term immunosuppression to balance graft protection and cancer risk.

## Highlights

• *De novo* malignancies remain a major long-term complication after liver transplantation.
• A nationwide target trial emulation using cloning, censoring and weighting evaluated long-term everolimus exposure.
• Everolimus initiation reduced de novo malignancy risk, whereas discontinuation increased the risk.
• These findings support sustained everolimus exposure as a potential strategy to reduce post-transplant cancer burden.

## Graphical abstract

**Figure:**
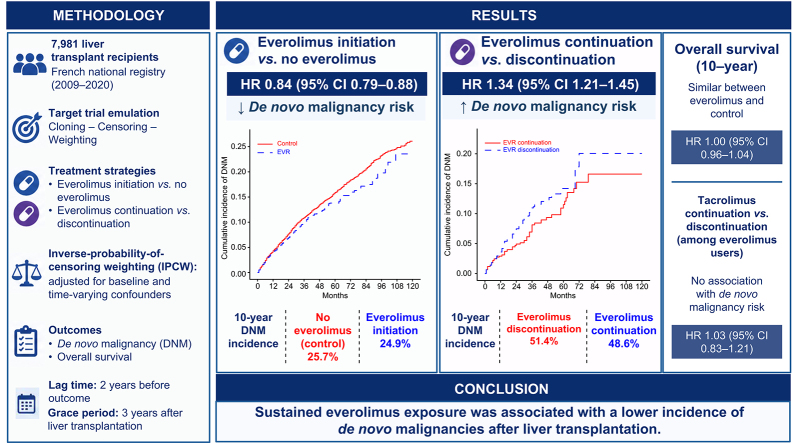

## Introduction

Liver transplantation (LT) is associated with an elevated risk of developing *de novo* malignancies (DNM).[1], [2], [3] This risk is largely attributable to the long-term immunosuppressive therapy that is essential to prevent allograft rejection but may impair antitumor immune surveillance.^4^ Immunosuppressive regimens vary in terms of their effects on rejection and cancer risk.^5^

Calcineurin inhibitors (CNIs) such as cyclosporine and tacrolimus (TAC) are standard first-line immunosuppressants^6^ but are linked to nephrotoxicity^7^ and cancer.^8^ These limitations have encouraged the adoption of mammalian target of rapamycin (mTOR) inhibitors.^9^ Everolimus (EVR) has so far been evaluated with and without CNIs.^10^^,^^11^ EVR improves renal function while maintaining immunosuppression when introduced early after LT.[10], [11], [12], [13] At higher doses, EVR exhibits antitumor activity,[14], [15], [16] thus supporting its use in LT recipients with prior hepatocellular carcinoma or an elevated cancer risk.^17^ However, due to concerns regarding wound-healing complications,^18^ EVR is generally a second-line option.

European guidelines recommend minimizing CNI exposure in patients with a high risk of hepatic or extrahepatic cancer recurrence, and suggest mTOR inhibitor-based regimens for recurrent or *de novo* non-melanoma skin cancer.^17^ However, the effects of mTOR inhibitors on post-transplant DNM or a recurrence of HCC in the native liver are unclear. Meta-analyses of retrospective studies have suggested that mTOR inhibitor-based immunosuppression may decrease HCC recurrence and improve survival, although study heterogeneity has limited any definitive interpretation.[19], [20], [21], [22], [23], [24] A recent Korean population-based study also reported the potential benefits of EVR in reducing HCC recurrence.^25^ The only randomized controlled trial to have evaluated mTOR inhibitors relative to HCC after LT did not demonstrate overall efficacy, although subgroup analyses indicated potential benefits in patients with more active tumors.^26^ Other evidence has shown that cumulative TAC exposure increases the incidence of post-transplant cancer.^27^ In kidney transplantation, mTOR inhibitors reduce non-melanoma skin cancer, although their effects on other malignancies remain uncertain.^28^^,^^29^

To date, no randomized trial has assessed the efficacy of EVR in preventing DNM after LT, largely because such trials require long-term follow-up and large samples.^30^ However, using nationwide French data, we were able to evaluate the effect of EVR on DNM after LT by applying a cloning-censoring-weighting (CCW) framework to mimic randomized trials.[31], [32], [33], [34] Our objective was to estimate the impact of EVR initiation on DNM incidence and post-LT survival, including the consequences of subsequent EVR discontinuation.^35^

## Materials and methods

### Ethics and data protection

This project was approved by the French National Commission for Information and Liberty (CNIL N°919274). No informed consent was required as the data were de-identified and re-used for research, in accordance with French regulations applicable to SNDS data.

### Data sources and measurements

The study population included patients discharged after their first LT, which had been required for decompensated cirrhosis (*e.g*. portal hypertension-related bleeding, ascites, and hepatic encephalopathy), primary liver tumors, or both, between January 1, 2009, and January 1, 2020. No exclusion criteria were applied.

This study used data from the French National Health Data System (*Système National de Données de Santé* – SNDS), covering nearly the entire French population of 67.7 million residents.^36^ The SNDS combines data from the *Datamart de Consommation Inter-Régimes* (DCIR), *Programme de Médicalisation des Systèmes d'Information* (PMSI), and CEPIDC.^37^ The DCIR captures outpatient healthcare procedures performed in ambulatory settings, while the PMSI records diagnoses and procedures associated with hospital care. The SNDS includes demographic data, hospital discharge reports, outpatient care information, and long-term sickness records.^38^ Diagnostic codes used ICD-10, procedures were identified using the Common Classification of Medical Procedures (CCMP) classification, and medications were recorded using CIP codes linked to the WHO ATC classification. Medication units were calculated from dispensing records, with treatment considered to be continuous if any interruptions lasted less than 90 days. If medication was being received within the final study year, treatment was considered to be ongoing until the end of the study. During hospitalization, the treatment days matched the length of stay. All patients received immunosuppressive treatment during the study period.

LT was identified using the CCMP procedure codes HLEA001 (total liver transplantation) or HLEA002 (partial liver transplantation).^39^ Hospital discharge after LT was defined as day 0. Chemotherapy and radiotherapy sessions were identified through ICD-10 codes, CCMP procedures, and ATC molecule codes. Treatment was confirmed if chemotherapy was ongoing at least 90 days after discharge.

The area of residence was defined by the French *‘département’* zip code at LT. For social deprivation assessments, we used municipality-level indicators that included percentages of unemployment, high school graduation, and those in work. The socioeconomic index was calculated as the mean of these indicators within their *département*.^40^ Patients born overseas were also identified.

Risk factors were collected, including tobacco use, alcohol use, metabolic syndrome, HIV, HBV, HCV prior to LT, and cancer history. The diagnostic algorithms used to identify liver transplant recipients, the ICD-10 codes for DNM classification and CCMP codes are shown in Tables S1–3.

### Outcome definition: DNMs

The primary outcome was the occurrence of any type of DNM. The secondary outcome was overall survival.

DNM occurrence was defined by the association of (i) an ICD-10 cancer diagnosis code, excluding *in situ* tumors, and (ii) a cancer-related CCMP procedure (surgery, radiotherapy, radiofrequency ablation, cryotherapy, insertion of vascular access for chemotherapy and the administration of chemotherapy). Each ICD-10 code was first classified according to tumor site (lung, head and neck, kidney and ureter, colorectal, breast, prostate, pancreas, and uterus) and then aggregated into broader cancer categories: gynecological, prostate, skin (excluding melanoma), lymphoma, colorectal, bladder, lung, and head and neck cancers. The time to DNM was defined as the interval between day 0 and the first occurrence of any DNM.

### Exposures

Three treatment strategies were examined. The first corresponded to the initiation of EVR within a grace period after discharge following the first LT. The second and third corresponded to the discontinuation of EVR or tacrolimus, respectively, within a grace period after EVR initiation.

### Statistical analysis

For descriptive purposes, continuous variables are presented as medians and interquartile ranges, and categorical variables as counts.

#### Cumulative incidence and overall survival

First, we estimated the cumulative incidence of all DNM types, site-defined DNMs, invasive cancer, and all-cause mortality. Time-to-event was defined as the interval between hospital discharge after LT and the occurrence of DNM or death. Survival following DNM was also evaluated. Patients were censored administratively on January 1, 2021, with administrative censoring assumed to be non-informative. Descriptive cumulative incidence curves were generated using competing-risk methods, with death as a competing event. To estimate the effects of EVR under a target trial emulation framework, we applied a CCW approach: each patient was duplicated and assigned to both treatment strategies at baseline, censored upon deviation from the assigned strategy, and weighted using inverse-probability-of-censoring weights. Event-time analyses were performed using weighted cause-specific Cox models.

#### Emulated trial 1: EVR initiation vs. no EVR

In emulated target trial 1 (ETT-1), we evaluated the effect of initiating EVR on DNM occurrence and survival. To emulate the target trial, we implemented a CCW approach (Table S4).

#### Cloning

At baseline (date of hospital discharge after liver transplantation), each participant was cloned into two copies: one assigned to the EVR initiation strategy and the other to the no-EVR strategy. Cloning ensured that both trial arms were identical with respect to baseline characteristics. Time zero was defined as the date of discharge after LT, when patients became eligible to receive EVR.

#### Grace period

Initiating EVR within 3 years (*grace period*^41^) was the "treatment" strategy; not initiating EVR within this period constituted the "control" strategy. During this period, participants assigned to the EVR strategy were allowed to initiate EVR at any time within 3 years of hospital discharge following LT. In contrast, participants assigned to the control strategy were expected not to initiate EVR during this time. The control arm received tacrolimus with or without mycophenolate mofetil (MMF). A 3-year grace period was selected because 88.1% of EVR-treated patients initiated therapy within this interval.

#### Censoring

Artificial censoring was applied when the observed treatment deviated from the assigned strategy. Artificial censoring may induce selection bias because censoring becomes informative across treatment arms. Clones were followed from baseline and were censored artificially when their observed treatment deviated from the assigned strategy, as described by Maringe *et al.*^41^ and Gaber *et al.*^34^ Specifically, clones assigned to the control strategy were censored at the time of EVR initiation if EVR was started within the 3-year grace period. Conversely, clones assigned to the EVR strategy were censored if they had not initiated EVR by the end of this 3-year grace period. This artificial censoring was applied according to the procedure described by Maringe *et al.*^41^ and Gaber *et al.*^34^

DNMs occurring during the grace period were counted as events under both strategies because individuals were cloned at baseline and followed under each treatment assignment. This prevented immortal time bias because event occurrence was not conditional on future treatment initiation.

#### Lag time

To estimate the effects of EVR, we defined a 2-year lag between EVR initiation and outcome to reduce any potential reverse causality. EVR could be initiated before hospitalization when a DNM was first coded, thus creating a potential bias. This meant that patients assigned to the EVR arm who received EVR within the grace period, but in whom we observed DNM within 2 years of treatment initiation, were censored at the time of treatment initiation. This was necessary because the data collected on those patients could only inform us about their survival between time zero and EVR initiation. This induced a selection bias due to conditionality on the future, with two important considerations: i) this bias was symmetrical across the two cloned arms (EVR *vs.* control) since the patients involved were censored at precisely the same time across the two arms; and ii) this selection bias was theoretically corrected by the weighting within both arms (under the assumption of no omitted variables).

#### Weighting

To address the selection bias introduced by artificial censoring, we computed a time-varying inverse probability of censoring weights. Thirty covariates were included to estimate the time-varying probability of remaining uncensored. Covariates were retained if they were clinically relevant to EVR use or to post-transplant outcomes (Figs. S2 and S3), or if they demonstrated significant associations with EVR initiation in generalized additive models (Table S5). All time-varying covariates were updated during follow-up before estimating the probability of remaining uncensored.

The selected covariates included both baseline characteristics and time-varying confounders. Baseline covariates included age at liver transplantation, sex, area-level income, area-level unemployment, tobacco use, alcohol-related liver disease, cardiovascular disease, diabetes mellitus, arterial hypertension, stroke, sleep apnea, psychiatric disorders, inflammatory bowel disease, HIV infection, hepatitis C infection, hepatitis B infection, hepatitis D coinfection, autoimmune liver disease, secondary biliary cirrhosis, ascites, hepatic encephalopathy, sepsis before transplantation, TIPS (transjugular intrahepatic portosystemic shunt) placement, pre-transplant hemodialysis (chronic and acute), extrahepatic cancer count before transplantation, any cancer before transplantation, and hepatocellular carcinoma as the primary indication for transplantation.

Time-varying confounders included tumor recurrence and renal dysfunction, which were considered key confounders because they are major clinical indications for EVR initiation after liver transplantation. EVR is frequently introduced in the context of post-transplant renal dysfunction or oncological indications, such as prior HCC or HCC recurrence. Some potential confounders, such as treatment-related adverse events, could not be accounted for because they are not captured in the SNDS database.

#### Covariate balance

Covariate balance before and after weighting was assessed, with the inverse probability of censoring weights truncated at the 99th percentile. Under the CCW framework, the weighted sample approximated a pseudo-population in which measured confounding and selection biases were minimized. We assumed that these selected covariates ensured exchangeability between censored and uncensored observations. To evaluate the reduction of measured confounding, we examined standardized mean differences (Table S6), and a Love plot is shown in Fig. S3. For ETT 1 to 3, we computed E-values for point estimates and confidence interval limits closest to the null in order to assess the robustness of treatment effects to unmeasured confounding.^42^

Numbers needed to treat (NNT) and numbers needed to harm (NNH) were derived from the absolute difference in cumulative incidence functions between treatment strategies at 10 years.^43^ The estimand was the hazard ratio (HR) among patients discharged after their first LT. 95% CIs for HRs were obtained using non-parametric bootstrapping with 500 replicates, repeating the entire CCW procedure at each replicate to capture overall estimation uncertainty.

#### Emulated trial 2: EVR discontinuation vs. EVR continuation

To assess the role of EVR, we evaluated the effect of EVR discontinuation on the DNM risk among patients who initiated EVR. EVR could be discontinued during the grace period after initiation, and a lag time was applied to account for the interval between EVR withdrawal and outcome occurrence. Day 0 was defined as the EVR initiation date. In ETT-2, the control arm corresponded to continuing EVR beyond the grace period with standard background immunosuppression, whereas the intervention arm included patients who discontinued EVR within 3 years of initiation.

#### Emulated trial 3: Discontinuation of TAC after EVR initiation

To determine whether the effects observed in ETT-1 and ETT-2 were confounded by the use of TAC, ETT-3 evaluated the impact of TAC discontinuation among patients who initiated EVR. Day 0 was defined as the EVR initiation date. The control arm corresponded to continuing TAC after EVR initiation, while the intervention arm comprised patients who discontinued TAC within the 3-year grace period. Both strategies could include MMF according to routine practice.

#### Sensitivity analyses for DNM occurrence and overall survival

The ETTs assessed site-specific associations between EVR initiation and individual cancer incidence. Sensitivity analyses examined alternative grace periods (1–4 years) and evaluated the impact of varying grace periods and lag times on ETT-1 estimates for DNM-free and overall survival. Additional analyses for ETT-1 restricted follow-up to the pre-2015 period to account for temporal changes in liver transplantation practice, including the introduction of direct-acting antivirals for hepatitis C infection. Analyses restricted to patients transplanted for HCC were also performed for ETT-1, given that approximately 44% of the cohort had HCC and 55% of everolimus-treated patients had a history of HCC. Alternative gap definitions (30, 60, and 180 days) were also evaluated for treatment discontinuation in ETT-2 to account for short interruptions, dose adjustments, or temporary holds of immunosuppressive therapy that may have been incompletely captured by dispensing data.

All statistical analyses used R statistical software (version 2023.12.0) with the following packages: ggplot2, ggalluvial, GGally, survival, survminer, parallel, boot, splines, dplyr, tableone, tidyr, questionr, forestmodel, WeightIt, ggplot, and cobalt.

## Results

### Demographic and clinical profiles of liver transplant recipients

The study population included 7,981 patients who underwent LT for decompensated cirrhosis (e.g., portal hypertensive bleeding, ascites, or hepatic encephalopathy), primary liver tumors, or both between January 1, 2009, and January 1, 2020. No exclusion criteria were applied. The cohort was 77.5% male, with a median age of 57 years (IQR 50–62). The leading indications for LT were alcohol-related liver disease (68.0%), HCV (28.3%), and HBV (11.5%). HCC was present in 44.0% of the recipients (Table 1).

**Table 1:** Baseline characteristics of the study population prior to emulation of the target trial.

Variable	Total (N = 7,981)	Control arm (n = 5,993)	EVR arm (n = 1,988)
Demographics			
Male sex	6,185 (77.5%)	4,872 (81.3%)	1,683 (84.7%)
Age at LT (years)	57 [50, 62]	56 [49, 62]	58 [53, 63]
Patients transplanted in Paris and suburbs	2,737 (34.3)	2,236 (36.0)	501 (28.4)
Years of unemployment	8.4 [6.9, 9.8]	8.4 [6.9, 9.8]	8.4 [ 7.1, 10.0]
Annual income (euros)	17,887 [16,708, 20,226]	17,887 [16,708, 20,315]	17,922 [16,804, 19,683]
Cirrhosis-related complications			
Ascites	4,564 (57.2%)	3,675 (57.1%)	889 (57.4%)
Hepatic encephalopathy	2,187 (27.4%)	1,789 (27.8%)	398 (25.7%)
Sepsis	1,818 (22.8%)	1,491 (23.2%)	327 (21.1%)
TIPS	461 (5.8%)	349 (5.4%)	112 (7.2%)
Variceal bleeding	2,872 (36.0%)	2,280 (35.4%)	592 (38.2%)
Liver disease etiology			
Alcohol-related cirrhosis	5,431 (68.0%)	4,259 (66.2%)	1,172 (75.6%)
HCC	3,542 (44.0%)	2,681 (41.7%)	861 (55.5%)
Primary biliary cholangitis	482 (6.0%)	429 (6.7%)	53 (3.4%)
Autoimmune	330 (4.1%)	284 (4.4%)	46 (3.0%)
Biliary cirrhosis	274 (3.4%)	238 (3.7%)	36 (2.3%)
HCV	2,260 (28.3%)	1,835 (28.5%)	425 (27.4%)
HBV	921 (11.5%)	754 (11.7%)	167 (10.8%)
HDV	217 (2.7%)	184 (2.9%)	33 (2.1%)
Risk factors and comorbidities			
Alcohol use	5,431 (68.0%)	4,259 (66.2%)	1,172 (75.6%)
Tobacco consumption	2,541 (32.0%)	2,040 (31.7%)	501 (32.3%)
Diabetes	2,719 (34.0%)	2,126 (33.1%)	593 (38.3%)
Arterial hypertension	5,643 (71.0%)	4,488 (69.8%)	1,155 (74.5%)
Stroke	222 (2.8%)	159 (2.5%)	63 (4.1%)
Cardiovascular events	1,969 (25.0%)	1,524 (23.7%)	445 (28.7%)
Sleep apnea	447 (5.6%)	354 (5.5%)	93 (6.0%)
Renal failure	1,199 (15.0%)	884 (14.2%)	315 (17.9%)
Psychiatric disorders	791 (9.9%)	651 (10.1%)	140 (9.1%)
IBD	287 (3.6%)	251 (3.9%)	36 (2.3%)
HIV	195 (2.4%)	175 (2.7%)	20 (1.3%)
Cancer before LT	385 (4.8%)	283 (4.4%)	102 (6.6%)

EVR, everolimus; HCC, hepatocellular carcinoma; IBD, inflammatory bowel disease; LT, liver transplantation; TIPS, transjugular intrahepatic portosystemic shunt.

Among the 7,981 eligible liver transplant recipients, 1,988 received EVR at least 2 years before DNM occurrence or the end of follow-up.

Quantitative variables are represented as medians [Q1, Q3], and categorical variables are expressed as numbers (%).

Arterial hypertension was present in 71.0% of patients, diabetes mellitus in 34.0% of patients, and 32.0% had a history of tobacco use. The initial post-LT immunosuppressive regimen included TAC in 90.5% and MMF in 91.5% of cases.

### Incidence of DNM and survival

The cumulative incidence of DNM was 15.1% at 5 years and 25.7% at 10 years post-transplantation (Fig. 2). The most frequent malignancies at 10 years were head and neck cancer (5.0%), skin cancer (5.0%), myeloid neoplasms (4.7%) and bladder cancer (4.5%). Other sites included prostate (2.0%), lymphoma (1.9%), colorectal (1.7%) and gynecological (0.7%) cancers.

**Fig. 1:**
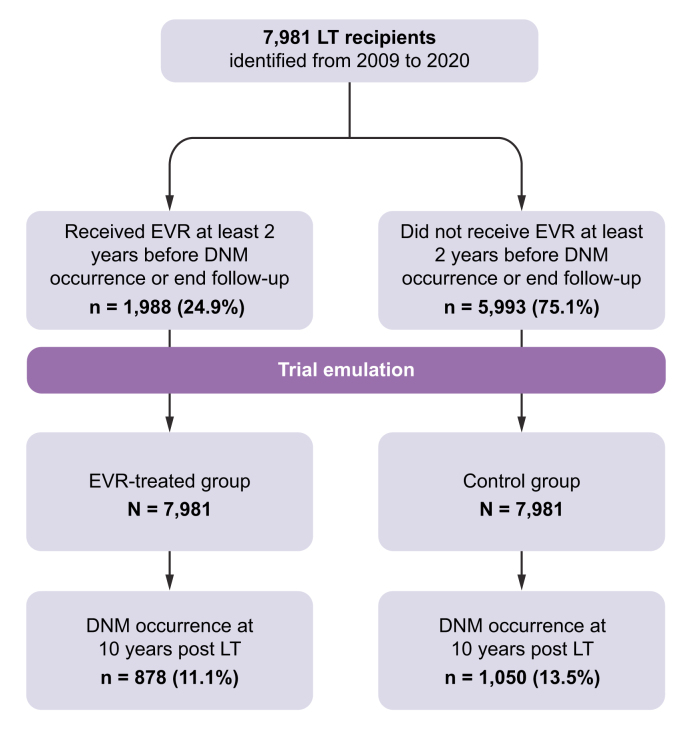
Study flowchart. Among 7,981 liver transplant recipients, 2,256 patients (28.3%) received EVR during follow-up. Of these, 1,988 (24.9% of the total cohort) initiated EVR at least two years before DNM occurrence or censoring. DNM, *de novo* malignancies; EVR, everolimus.

**Fig. 2:**
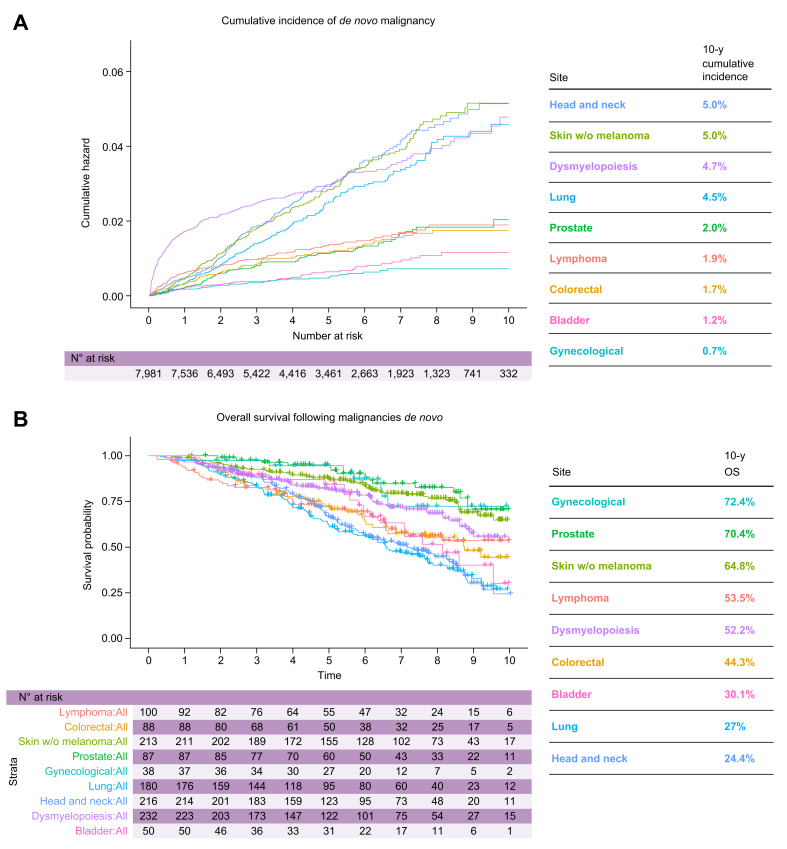
Cumulative incidence of DNM after liver transplantation and 10-year overall survival following DNM. Five- and ten-year cumulative incidence estimates of DNM were stratified by cancer site. Head and neck, non-melanoma skin, myeloid neoplasms, and bladder cancers were the most frequent malignancies found at 10 years (A). Kaplan–Meier estimates of 10-year overall survival after DNM diagnosis are shown by cancer site; head and neck, lung, and bladder cancers exhibited the lowest survival rates (B). DNM, *de novo* malignancies; EVR, everolimus.

Ten-year survival following DNM onset depended on the cancer type (Fig. 2). Head and neck (24.4%), lung (27.0%), and bladder (30.1%) cancers had the lowest survival rates. Skin (64.8%), prostate (70.4%), and gynecological (72.4%) cancers had the highest survival rates.

#### Emulated trial 1: EVR initiation *vs*. no EVR

During follow-up, 2,256 patients (28.3%) received EVR, 1,988 of whom (24.9% of the total cohort) initiated EVR at least 2 years before DNM occurrence or censoring (Fig. 1). Among patients treated with EVR within this grace period, concomitant immunosuppression included tacrolimus (n = 966; 48.9%), MMF (n = 486; 24.5%), and cyclosporine (n = 98; 4.9%).

After applying the clone–censor–weight approach, EVR initiation was associated with a lower 10-year risk of DNM (HR 0.84; 95% CI, 0.79–0.88). Based on the 10-year risk difference in the CCW-weighted pseudo-population (median RD –0.065), the NNT to prevent one DNM case was 16 (IQR, 10–23). The E-value for the point estimate was 1.67, indicating that an unmeasured confounder would need to be associated with both EVR initiation and DNM risk by a risk ratio of at least this magnitude (above and beyond measured covariates) to fully account for the observed association (Table S7).

**Fig. 3:**
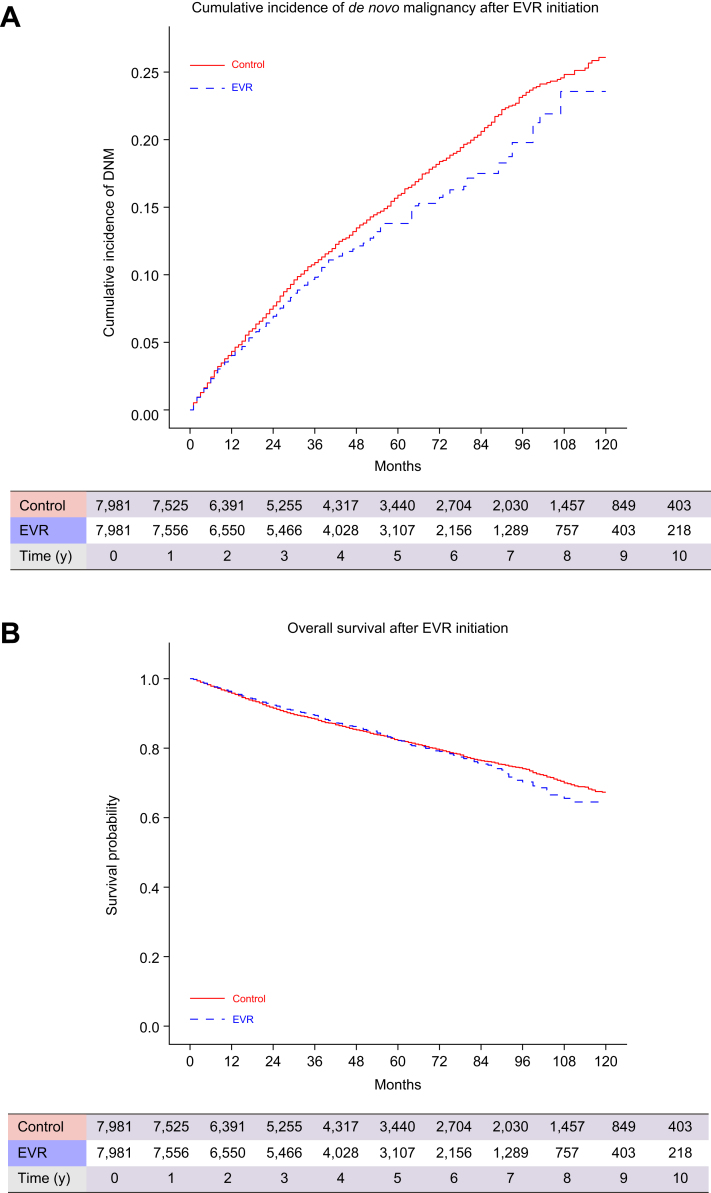
Cumulative probability of DNM and overall survival in emulated target trial 1 (EVR initiation *vs.* No EVR). Cumulative probability of DNM occurrence (A) and overall survival (B) over 10 years, comparing patients who initiated EVR *vs*. those who did not. Estimates were based on a 3-year grace period and a 2-year lag time. A total of 1,988 patients who initiated EVR within this framework were included in the treatment arm. Nelson–Aalen and Kaplan–Meier curves were weighted using the cloning–censoring–weighting method. EVR initiation was associated with a reduced risk of DNM (HR 0.84; 95% CI 0.79–0.88). Ten-year overall survival was similar between the arms (HR 1.00; 95% CI 0.96–1.04). DNM, *de novo* malignancies; EVR, everolimus; HR, hazard ratio.

Ten-year overall survival rates were similar in both arms. The HR for mortality was 1.00 (95% CI 0.96–1.04) (Fig. 3).

**Fig. 4:**
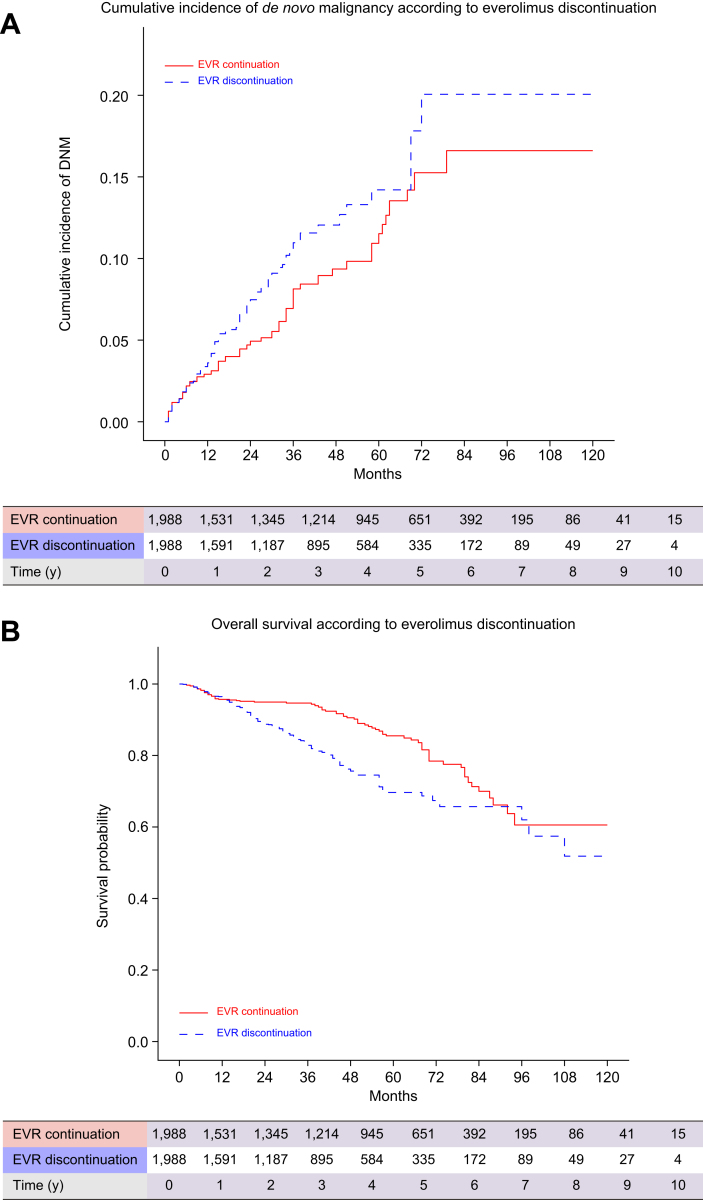
Cumulative probability of DNM and overall survival in emulated target trial 2 (EVR continuation *vs.* discontinuation). Cumulative probability of DNM occurrence (A), and 10-year overall survival among patients who continued EVR *vs.* those who discontinued it after initiation (n = 1,988). Analyses were based on a 3-year grace period and a 2-year lag time. A total of 675 patients were included in the EVR discontinuation arm. Nelson–Aalen and Kaplan–Meier curves were weighted using the cloning–censoring–weighting method. EVR discontinuation was associated with an increased risk of DNM (HR 1.34; 95% CI 1.21–1.45) and showed no significant effect on overall survival (HR 1.00; 95% CI 0.88–1.13). DNM, *de novo* malignancies; EVR, everolimus; HR, hazard ratio.

### Emulated trial 2: EVR discontinuation *vs*. continuation

ETT-2 included 1,988 patients who initiated EVR. Time zero was defined as the date of EVR initiation. The discontinuation of EVR within a 3-year grace period was compared with continued EVR use beyond this period, applying a 2-year lag. Overall, 967 patients (48.6%) discontinued EVR.

EVR discontinuation was associated with a significantly higher risk of DNM, with a risk difference of 0.0505 (95% CI 0.0341–0.0765) and an HR of 1.34 (95% CI 1.21–1.45) (Fig. 4), corresponding to a NNH of 20 (95% CI 13–29). The E-value was 1.84 (lower bound 1.56), suggesting moderate robustness to unmeasured confounding (Table S7).

**Fig. 5:**
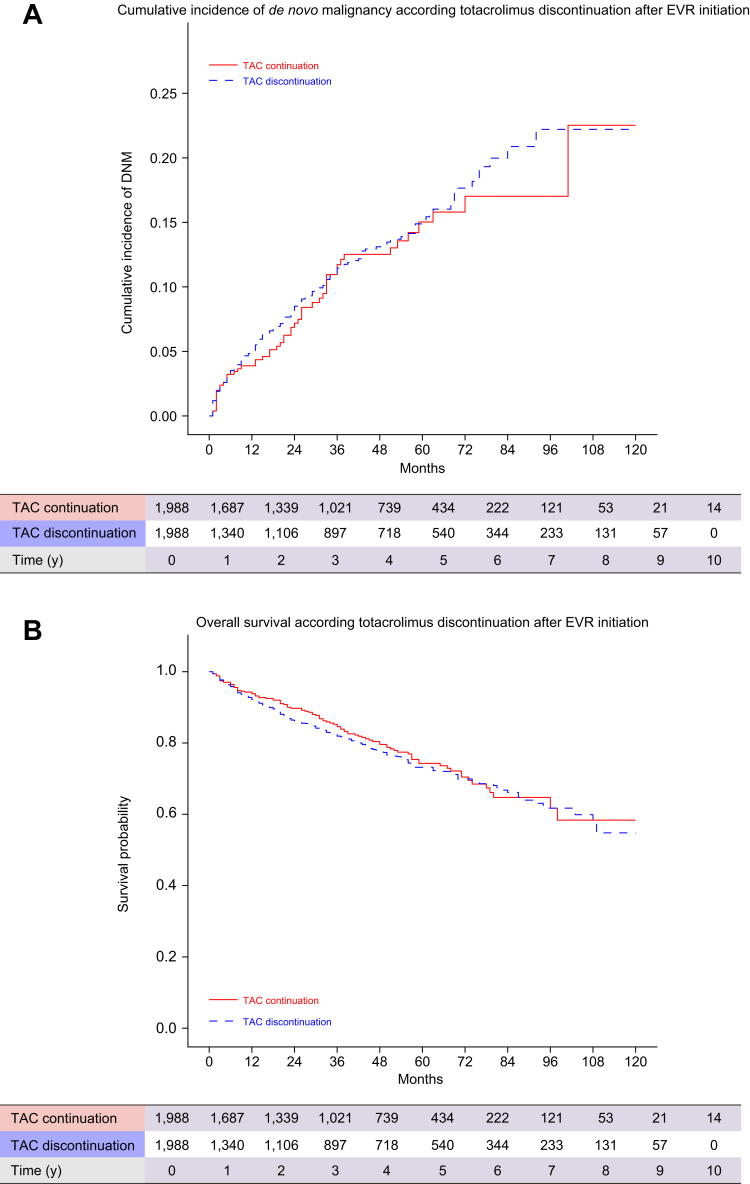
Cumulative probability of DNM and overall survival in emulated target trial 3 (discontinuation of tacrolimus after EVR initiation). Cumulative probability of DNM occurrence (A) and overall survival (B) over 10 years among EVR initiators (n = 1,988), comparing patients who continued TAC *vs*. those who discontinued it. Analyses used a 3-year grace period and a 2-year lag time. A total of 1,626 patients were included in the TAC discontinuation arm. Nelson–Aalen and Kaplan–Meier curves were weighted using the cloning–censoring–weighting method. The 10-year risk of DNM was similar between the arms (HR 1.03; 95% CI 0.83–1.21), as was overall survival (HR 1.00; 95% CI 0.92–1.07). DNM, *de novo* malignancies; EVR, everolimus; HR, hazard ratio; TAC, tacrolimus.

### Emulated trial 3: Discontinuation of TAC after EVR initiation

Among the 1,988 patients who initiated EVR, 1,626 (81.8%) discontinued TAC within the 3-year grace period and a 2-year lag-time. The 10-year HR for DNM occurrence was similar across the different arms (HR 1.03; 95% CI 0.83–1.21) (Fig. 5). The corresponding risk difference was 0.1258 (95% CI 0.1159–0.1388) yielding an NNH of 8 (95% CI 7–9). The E-value for the point estimate was 1.16 (lower bound 1.00), indicating that a weak unmeasured confounder would be sufficient to move the confidence interval to include the null.

### Site-specific analysis ETT

Site-specific analyses were performed using the same grace periods and lag times applied in ETT-1. EVR initiation was associated with a reduced incidence of bladder (HR 0.58; 95% CI 0.52–0.61), hematological (HR 0.96; 95% CI 0.95–0.98), lung (HR 0.90; 95% CI 0.72–0.96), prostate (HR 0.64; 95% CI 0.59–0.73), skin (HR 0.92; 95% CI 0.88–0.94), and lymphoma (HR 0.77; 95% CI 0.73–0.81) malignancies. No effect was observed among head and neck (HR 1.04; 95% CI 0.99–1.11), colorectal (HR 1.05; 95% CI 1.04–1.11), or gynecological (HR 1.09; 95% CI 0.86–1.33) cancers (Fig. S4).

### Sensitivity analysis

**Fig. 6:**
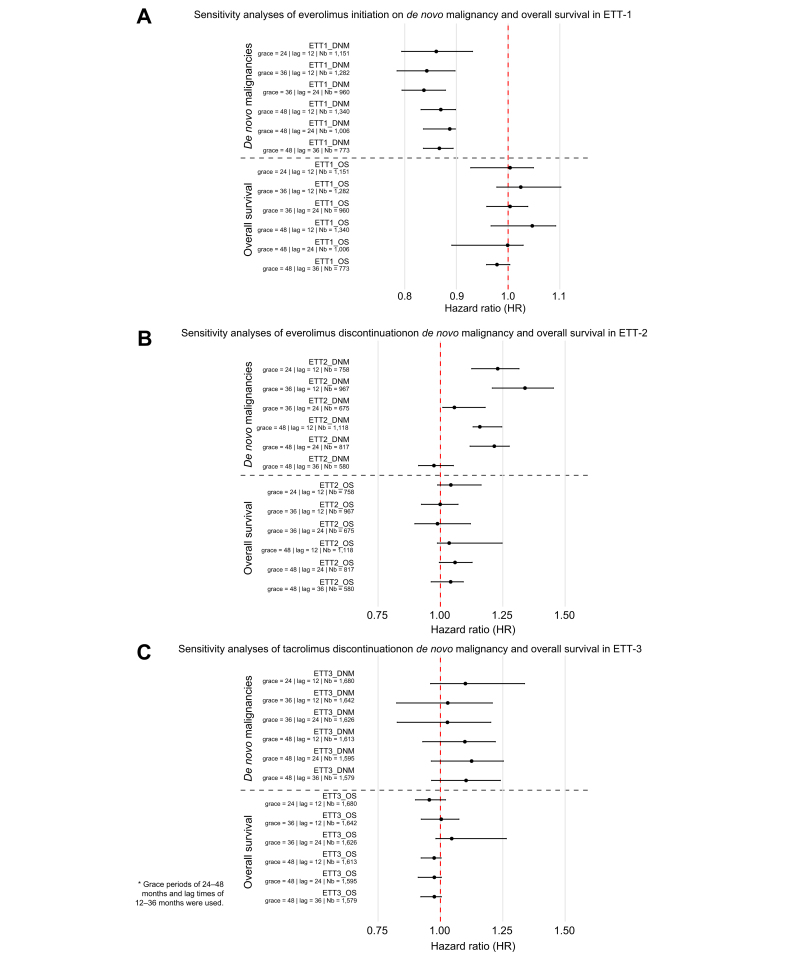
Sensitivity analysis across different grace period and lag time scenarios. Six parameter combinations were evaluated for each emulated target trial, varying the grace period (24, 36, or 48 months) and lag time (12, 24, or 36 months), with the constraint that grace ≠ lag time. All analyses used a 10-year follow-up period. Across all combinations, ETT-1 showed a consistent protective effect of EVR on DNM and no effect on overall survival (A). ETT-2 consistently showed an increased DNM risk associated with EVR discontinuation, except for the combination of grace = 48 and lag = 36 months (B). For ETT-3, all parameter combinations yielded similar results for DNM and overall survival, indicating no measurable influence of TAC (C). DNM, *de novo* malignancies; ETT, emulated target trial; EVR, everolimus; TAC, tacrolimus.

Varying the grace periods and lag times changed the number of treated patients, potentially influencing any previously observed associations. For each ETT, using a 10-year follow-up period, we tested six combinations: a grace period of 1–4 years and lag times of 1–3 years, excluding combinations in which the lag time equaled the grace period. In ETT-1, all six combinations showed a protective effect of EVR on DNM risk and no effect on overall survival. In ETT-2, an increased DNM risk was observed for all but one combination (grace period = 4 years, lag time = 3 years), where the number of treated patients was small because of the prolonged lag time (Fig. 6). In ETT-3, all combinations yielded similar results for 10-year DNM incidence and overall survival. We also evaluated lag time = 0. In ETT-1, the association was attenuated (Fig. S4), suggesting some degree of reverse causality when no lag was applied (EVR was prescribed because a DNM had been detected in an outpatient setting, and the DNM was coded later in the electronic health records). In ETT-2, removing the lag time had no effect, likely because DNM detection did not prompt EVR discontinuation.

The weighting phase of the CCW approach is critical. The weighting of our covariates was verified using a paired t-test, as shown in `Fig. S4.

Restricting analyses to the pre-2015 period yielded results consistent with the primary analysis for both DNM-free survival (HR 0.85, 95% CI 0.81–0.88) and overall survival (HR 1.04, 95% CI 0.92–1.10) (Fig. S6).

Analyses restricted to patients transplanted for HCC showed similar results, with an HR of 0.87 (95% CI, 0.72–0.90) for DNM occurrence and an HR of 0.99 (95% CI, 0.98–1.01) for overall survival (Fig. S7).

Sensitivity analyses using alternative gap definitions for treatment discontinuation (30, 60, and 180 days) produced estimates consistent with the main findings, with HRs for DNM occurrence ranging from 1.40 to 1.60 and for overall survival from 1.41 to 2.03 (Table S8).

## Discussion

This study used emulated target trials to evaluate the association between EVR use and the risk of *de novo* malignancy after LT. EVR initiation was associated with a lower probability of DNM, whereas EVR discontinuation was associated with an increased risk of DNMs, and no association was observed with a concomitant discontinuation of tacrolimus.

The antitumor properties of mTOR inhibitors are established at doses higher than those used post-transplantation. At lower post-LT doses, EVR likely exerts its effects through immunomodulatory mechanisms rather than direct cytotoxicity. EVR did not confer uniform protection across DNM types. Reduced incidences were observed in bladder, lung, hematological, prostate, skin, and lymphoid cancers but not head and neck, colorectal, or gynecological cancers, suggesting tumor-specific differences in immune sensitivity.

Nearly half of the patients who initiated EVR discontinued this treatment during follow-up. Although the SNDS did not capture the reasons for discontinuation, one potential explanation concerns EVR-related adverse effects and immunosuppression minimization in stable recipients. The ETT-2 trial showed that continued EVR use was associated with a lower risk of DNM.

Tacrolimus discontinuation among EVR initiators was not associated with a detectable change in the DNM risk. However, this finding should be interpreted with caution. In clinical practice, EVR initiation is typically accompanied by tacrolimus minimization, and detailed pharmacokinetic data on tacrolimus exposure were not available in the SNDS database. The literature on liver transplantation indicates that the oncologic risk is more strongly related to the intensity and timing of calcineurin inhibitor exposure – particularly high tacrolimus levels soon after transplantation and cumulative exposure during the first post-transplant year – rather than to prescription status alone.^44^^,^^45^ Consequently, the present analysis cannot exclude the possibility that the observed association between EVR exposure and lower DNM incidence partly reflected reduced calcineurin inhibitor exposure rather than a direct antineoplastic effect of EVR itself. In addition, most EVR-treated patients (81.8%) discontinued tacrolimus within the grace period, which reduced the contrast between continuation and discontinuation strategies and limited statistical power to detect differences between these groups. Taken together, these findings suggest that sustained EVR exposure may be associated with a lower probability of DNM after liver transplantation, but the relative contribution of EVR itself *vs.* concomitant calcineurin inhibitor minimization could not be determined in the absence of detailed exposure data.

While EVR initiation significantly reduced the risk of DNMs, this benefit did not translate into improved 10-year overall survival. This outcome is not necessarily discordant, given several contributing factors. Firstly, post-liver transplant mortality is multifactorial, with malignancies accounting for a limited proportion of deaths (approximately 30% in published series^46^), thereby limiting the overall impact of a moderate reduction in DNM incidence (HR 0.84). Moreover, overall survival is a non-specific endpoint that may lack the power to detect an indirect effect from a moderate reduction in DNM, particularly because only one-quarter of our cohort developed DNM. Secondly, the cancer types most effectively reduced by EVR (skin, prostate, hematologic, and lymphoid cancers) are generally associated with relatively favorable survival rates (53–72%). Conversely, the rates of cancers with the poorest prognosis, such as head and neck (24.4%), lung (27%), and bladder (30.1%), were either not reduced or only modestly affected by EVR. Finally, nearly half of the patients initiating EVR discontinued this treatment during follow-up, which likely attenuated any potential long-term survival benefits. Our inability to specifically assess cancer-related mortality, due to the inconsistent reporting of causes of death, further limited our understanding of this relationship.

The 10-year cumulative incidence of DNM (25.7%) was consistent with the findings of previous French studies, particularly regarding head and neck and skin cancers. The lower incidence of skin cancer in our cohort likely reflects stricter inclusion criteria that only captured invasive cancers requiring hospitalization. The relatively high incidence of hematological malignancies could be explained by coding practices in the administrative databases, whereby chronic hematological conditions – often diagnosed and managed in an outpatient setting – are recorded during hospitalizations related to liver transplantation or its follow-up. Survival rates after DNM diagnosis were also in line with findings in the literature, underscoring the long-term burden of malignancies after LT.[47], [48], [49], [50], [51], [52], [53]

We applied a CCW framework that relied on three assumptions: positivity (non-zero probability of EVR treatment), consistency (well-defined exposure), and exchangeability (addressed through CCW). Each emulated trial demonstrated adequate covariate balance, and the findings were robust across parameter variations. The SNDS database offers a comprehensive, longitudinal follow-up of the French population, enabling an accurate assessment of drug exposure, comorbidities, and outcomes. Its completeness ensures strong external validity, consistent with international recommendations for real-world evidence in drug evaluation.^51^^,^^52^

This study had some limitations. Only cancers requiring hospitalization were captured, potentially missing outpatient diagnoses, although the use of a lag period helped to mitigate this risk. In the SNDS, outpatient procedures recorded in the DCIR enable the identification of dermatologic excisions and oncologic treatments performed in ambulatory settings, although diagnostic codes do not always specify the underlying malignancy. Consequently, malignancies commonly diagnosed and treated in outpatient settings (particularly skin cancers) may not have been fully captured. Histological subtypes and TNM staging were unavailable. Cause of death coding was inconsistent, and this prevented any determination of cancer-specific mortality. Donor–recipient linkages were not available, limiting any evaluation of donor-related factors. Information on induction therapy, the treatment of acute rejection, or exposure to lymphocyte-depleting agents (known determinants of post-transplant lymphoproliferative disorders) was not available in the SNDS database. As with any studies using administrative claims data, misclassification was possible, although prior validations supported the accuracy of the SNDS in terms of identifying cancer.^53^ Medication adherence could not be confirmed, potentially underestimating the effects of initiation or discontinuation. Unmeasured confounding within the grace period could have weakened causal interpretation, although such confounders would affect both drug initiation and discontinuation decisions.

In conclusion, *de novo* malignancies represent a major long-term challenge after liver transplantation, underscoring the importance of sustained cancer surveillance, particularly for tumors amenable to early detection. Using an emulated target trial framework applied to a nationwide cohort, this study suggests that sustained exposure to EVR is associated with a lower incidence of DNMs. These findings support immunosuppressive strategies that incorporate calcineurin inhibitor minimization, including EVR-based regimens, as a strategy to potentially reduce the long-term oncologic risk after liver transplantation.

## Abbreviations

CCMP, common classification of medical procedures; CCW, cloning-censoring-weighting; CNI, calcineurin inhibitors; DCIR, Datamart de Consommation Inter-Régimes (National Health Insurance Reimbursement Database); DNMs, *de novo* malignancies; ETT, emulated target trials; EVR, everolimus; HCC, hepatocellular carcinoma; LT, liver transplantation; mTOR, mammalian target of rapamycin; MMF, mycophenolate mofetil; NNH, number needed to harm; NNT, number needed to treat; PMSI, Programme de médicalisation des systèmes d'information (French Hospital Discharge Database); SNDS, French National Health Data System (Système National de Données de Santé); TAC, tacrolimus.

## Authors’ contributions

Ilias Kounis, Tri-Long Nguyen, Paul Landais and Cyrille Feray contributed to the conception and design of this study and to preparation and finalization of the manuscript. Ilias Kounis, Tri-Long Nguyen, Paul Landais and Cyrille Feray performed the statistical analysis. Christophe Desterke, Nathalie Goutte, Paul Landais and Cyrille Feray participated in data collection. All authors critically reviewed the manuscript for important intellectual content and approved the final draft for submission.

## Data availability

According to French regulations on data protection, the authors cannot publicly release data from the French National Health Data System (*Système National de Données de Santé* - SNDS). However, any person or structure, public or private, for profit or non-profit, can access SNDS data following authorization from the French Data Protection Office (Commission *Nationale de l’Informatique et des Libertés*) to carry out a study or an evaluation of public interest.

## Ethical registration

The project was approved by the French National Commission for Information and Liberties (CNIL) N°919274.

## Prior presentations

These findings were presented at the International Liver Transplantation Society (ILTS) Congress in Singapore, May 2025, and at the European Association for the Study of the Liver (EASL) Congress in Milan, June 2024.

## Financial support

The authors declare that no financial support was provided for this study or compilation of this manuscript.

## Conflict of interest

The authors declare no conflicts of interest.

Please refer to the accompanying ICMJE disclosure forms for further details.
