# Supplementary material for: Everolimus and de novo malignancies after liver transplantation: A nationwide emulated trial using real-world data
Source: JHEP Rep. 2026 Aug 7;8(9):101934. doi: 10.1016/j.jhepr.2026.101934 (PMC13476556; doi:10.1016/j.jhepr.2026.101934)
Supplement: Supplementary file 1 [file mmc1.pdf]

Everolimus and *de novo* malignancies after liver transplantation: A nationwide emulated trial using real-world data

Ilias Kounis, Tri-Long Nguyen, Christophe Desterke, Nathalie Goutte, Didier Samuel, Daniel Azoulay, Eric Vibert, Faouzi Saliba, Audrey Coilly, Paul Landais, Cyrille Féray

Table of contents

Supplementary figure legends..2
Supplementary table legends..3
Supplementary figures..4
Supplementary tables..8

Supplementary figure legends

Fig. S1. Illustration of patient trajectories and their corresponding cloned treatment strategies in a target trial emulation of Everolimus (EVR) in terms of preventing *de novo* malignancies after liver transplantation. Each real patient contributed two clones at baseline, randomly assigned to either the "EVR strategy" or the "Control strategy". The grace period (vertical dashed blue line) represents the window allowed for EVR initiation. Patients were followed from transplantation until *de novo* malignancy (DNM), administrative censoring, loss to follow-up, or death (all collapsed into censoring). As an illustration, we display nine representative scenarios: Scenarios 1A–1C: EVR initiated within the grace period. The Control clones are censored at the time of EVR initiation in the real patient (protocol deviation). Scenarios 2A–2D: No EVR initiated during the grace period. The EVR clones are censored at the end of the grace period. Scenarios 3A–3B: Late initiation of EVR (after the grace period). The Treated clones are censored at EVR initiation.

Fig. S2. Directed acyclic graph of the causal structure between Everolimus treatment and *de novo* malignancy or overall survival. Age and sex, comorbidities and lifestyle factors (tobacco and alcohol use), socioeconomic status, underlying liver disease and liver function before transplantation, baseline kidney function, and pre-transplant malignancy were considered baseline confounders of the association between Everolimus treatment and *de novo* malignancy or overall survival. Everolimus may also influence the recurrence of post-transplant chronic kidney disease and hepatocellular carcinoma (HCC), which are considered post-transplant mediators and were therefore not included in the adjustment set.

Fig. S3: Covariate balance before and after inverse probability weighting. Absolute standardized mean differences (SMDs) are shown for baseline characteristics before (red circles) and after (blue triangles) inverse probability treatment weighting (IPTW). Vertical dashed lines indicate SMD thresholds of 0.1, below which covariates are considered to be well balanced between treatment arms. After weighting, all covariates achieved adequate balance, supporting a successful reduction of measured confounding.

Fig. S4: Site-Specific Cumulative Incidence of *De Novo* Malignancies. Cumulative incidence curves for DNM by cancer site, comparing patients who initiated EVR versus those who did not. The analysis was based on a 3-year grace period and 2-year lag time. Site-specific curves highlight the differences in EVR-associated risk across tumor types, including bladder, hematologic, lung, prostate, skin, lymphoma, head and neck, colorectal, and gynecological cancers.

Fig. S5: Sensitivity analysis using zero lag time for ETT1 and ETT2.

Fig. S6: Cumulative incidence of de novo malignancy after everolimus initiation for patients transplanted before end of 2015 with 3-year grace period and 2-year lag time

Fig. S7: Cumulative incidence of de novo malignancy after everolimus initiation for patients transplanted for HCC with 3-year grace period and 2-year lag time.

Supplementary table legends

Table S1: Algorithm used to identify liver transplant recipients from the French national healthcare database.

Table S2: List of ICD-10 codes used to define cancer diagnoses for DNM classification.

Table S3: CCMP procedure codes used to confirm the diagnosis of DNM.

Table S4: Summary of protocol components of the emulated target trials.

Table S5: Multivariate Generalized Additive Model (GAM). This approach estimates adjusted effects for each covariate on the hazard of death or retransplantation. This table reports the estimated coefficients (Estimate), standard errors (SE), z-values and corresponding p-values. Variables with $p < 0.05$ were considered to be statistically significant.

Table S6: Covariate balance before and after inverse probability of censoring weighting.

Table S7: Effects of treatments in trials ETT-1 to ETT-3: risk difference (RD), hazard ratio (HR), number needed to treat/harm (NNT/NNH), and E-value.

Table S8: Sensitivity analyses of ETT-2 using alternative dispensing-gap definitions (30, 60, and 180 days) to define treatment discontinuation

Supplementary figures

Supplementary Figure 1

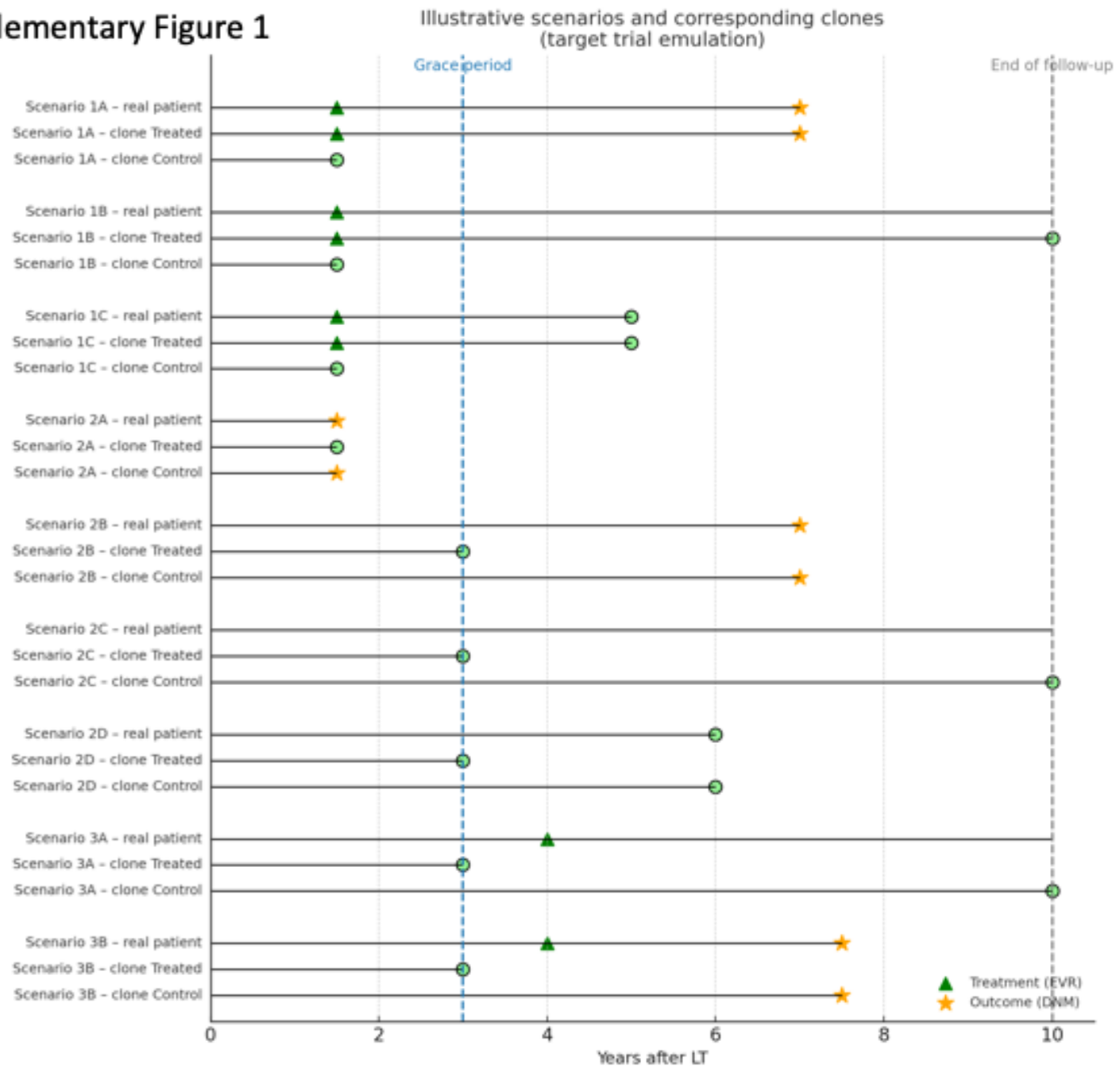

Hypothetical analysis dataset
for the target trial

Scenario	Arm	δ (event)	T* (years)
1A	Treated	1	7.0
1A	Control	0	1.5
1B	Treated	0	10.0
1B	Control	0	1.5
1C	Treated	0	5.0
1C	Control	0	1.5
2A	Treated	0	1.5
2A	Control	1	1.5
2B	Treated	0	3.0
2B	Control	1	7.0
2C	Treated	0	3.0
2C	Control	0	10.0
2D	Treated	0	3.0
2D	Control	0	6.0
3A	Treated	0	3.0
3A	Control	0	10.0
3B	Treated	0	3.0
3B	Control	1	7.5

Supplementary Figure 2

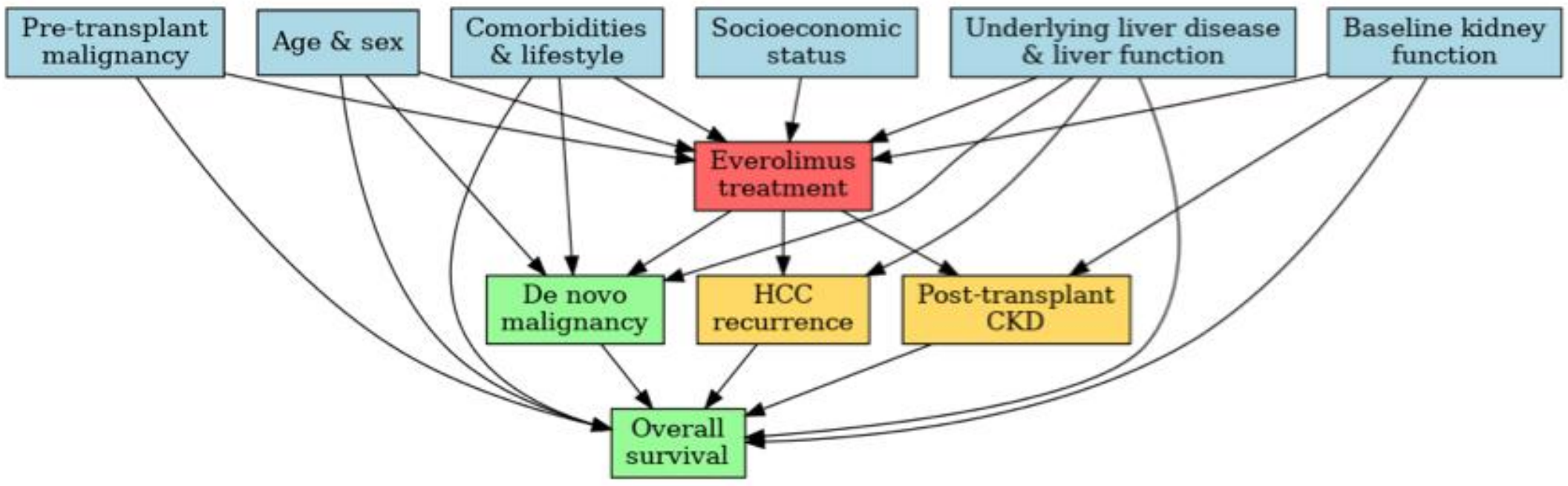

Supplementary Figure 3

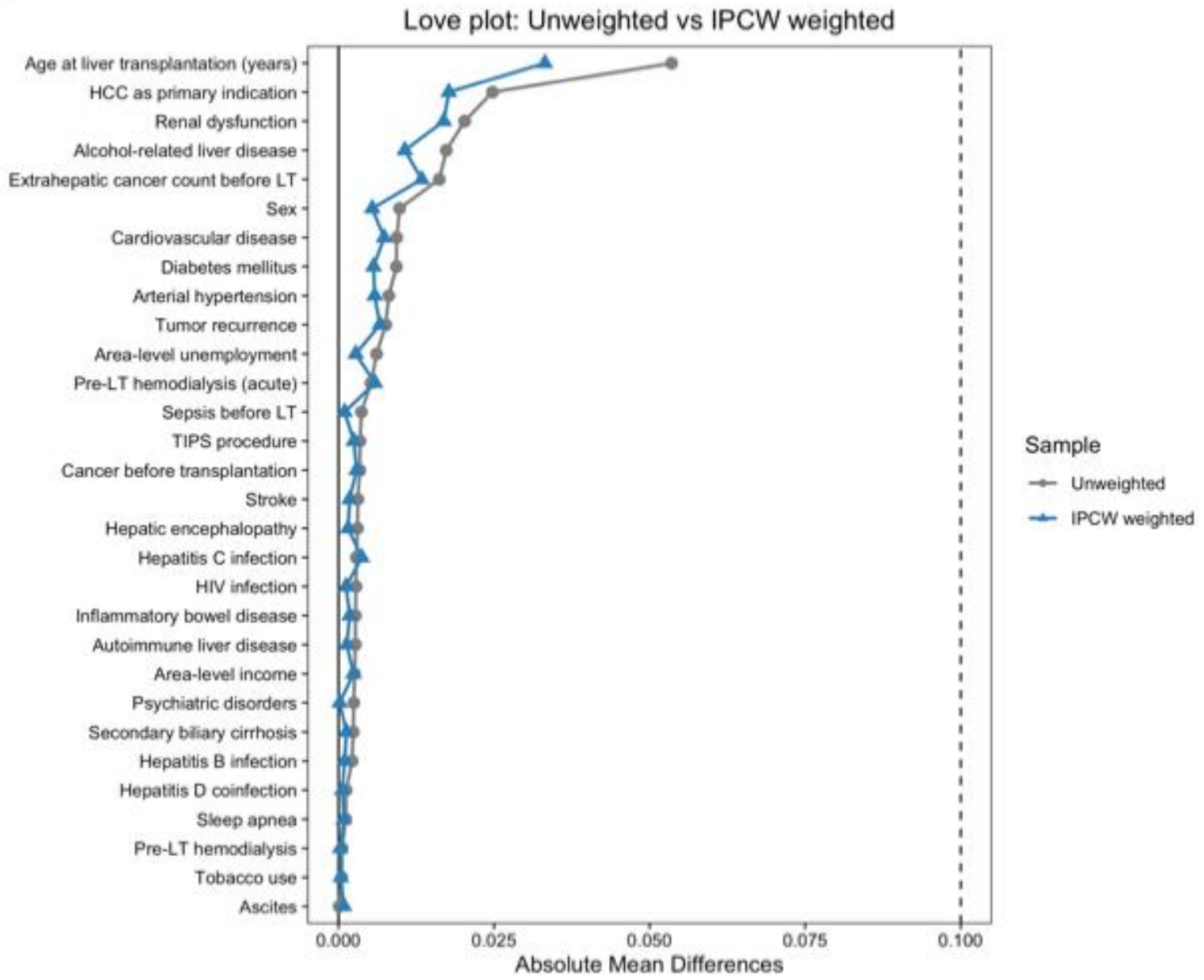

Supplementary Figure 4

Site-Specific Cumulative Incidence of *De Novo* Malignancies

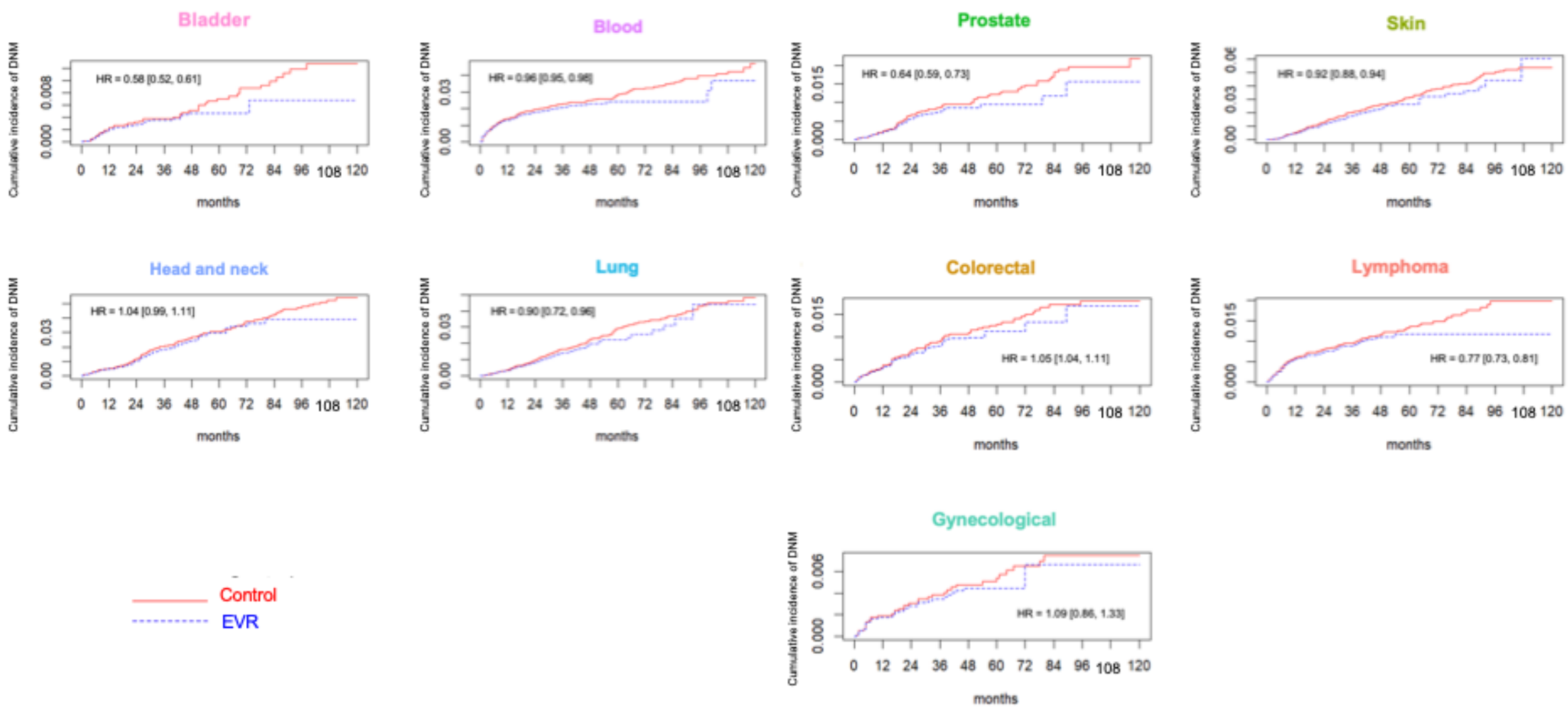

Supplementary Figure 5

Cumulative incidence of de novo malignancy after everolimus initiation using different grace periods (lag time = 0).

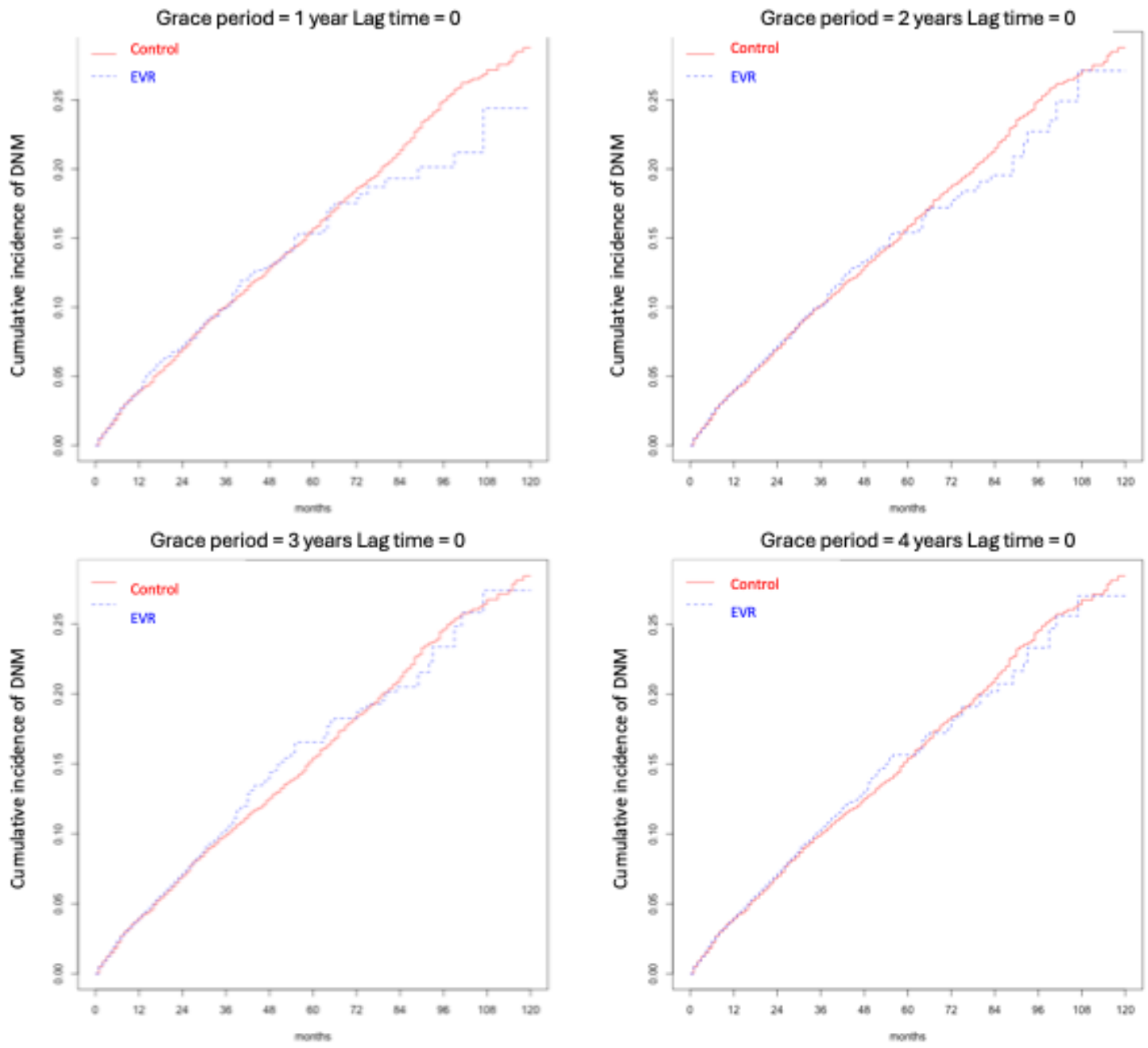

Supplementary Figure 5

Cumulative incidence of de novo malignancy after everolimus discontinuation using different grace periods (lag time = 0).

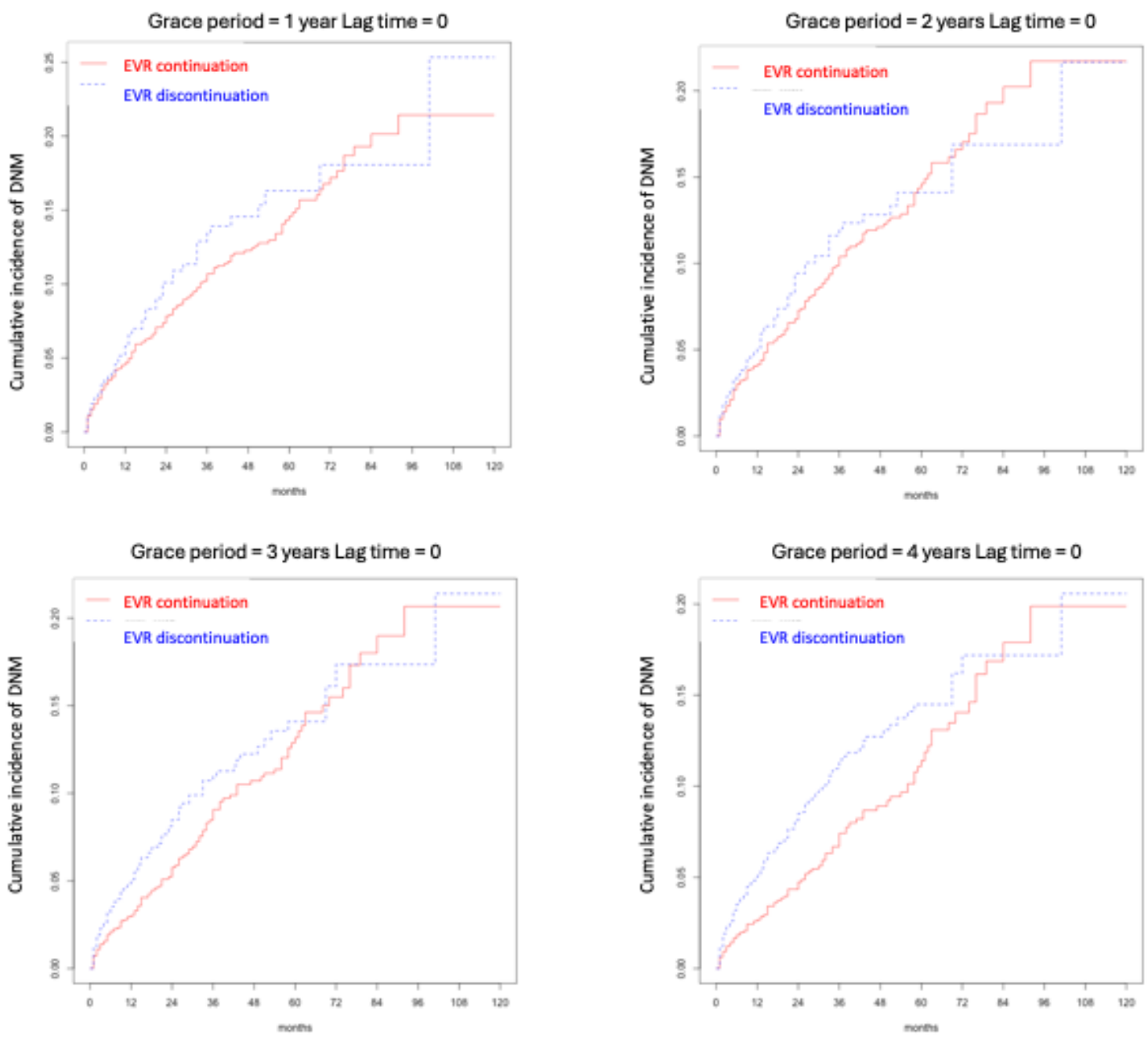

Supplementary Figure 6

Cumulative incidence of de novo malignancy after everolimus initiation for patients transplanted before end of 2015 with 3-year grace period and 2-year lag time

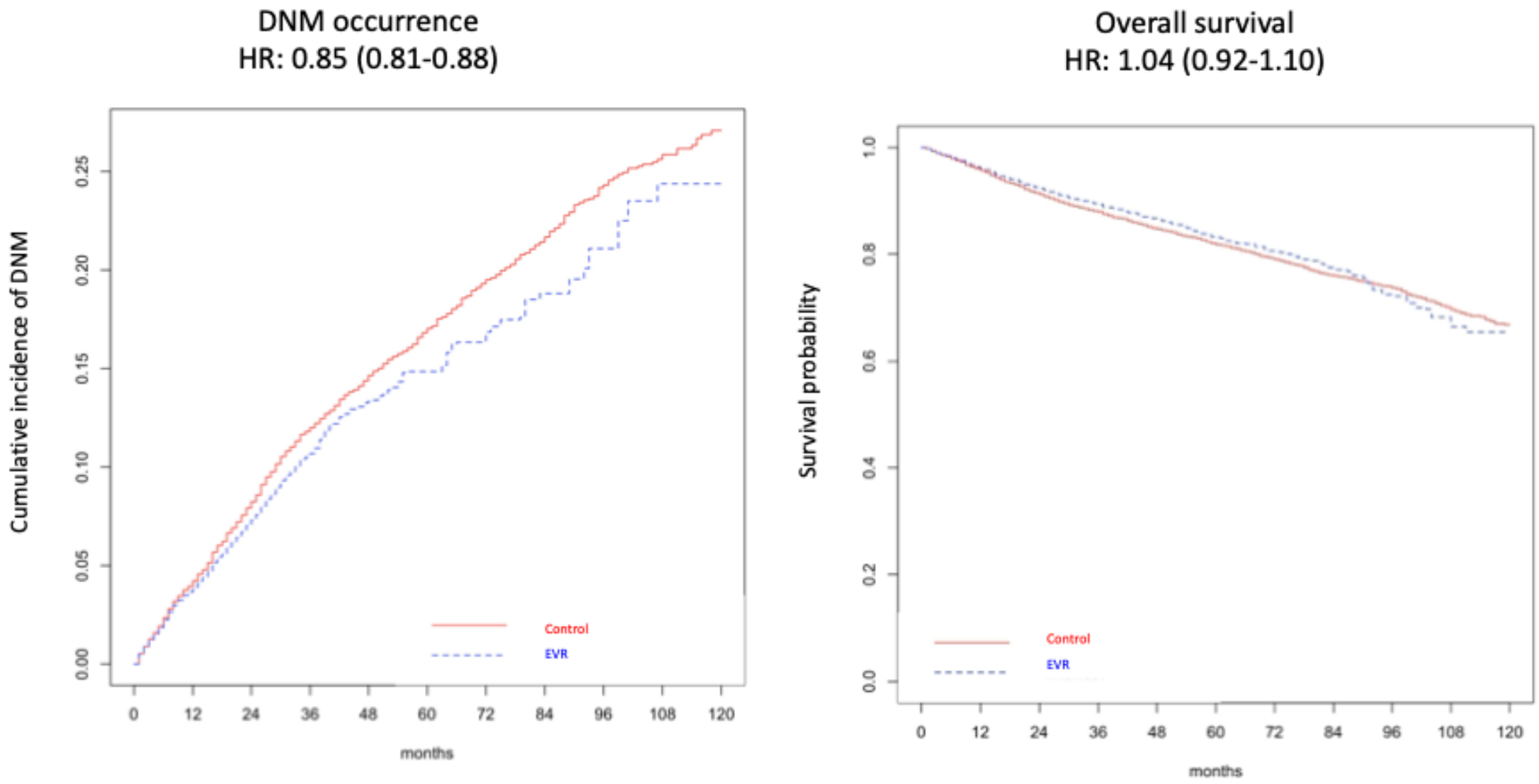

Supplementary Figure 7

Cumulative incidence of de novo malignancy after everolimus initiation for patients transplanted for HCC with 3-year grace period and 2-year lag time

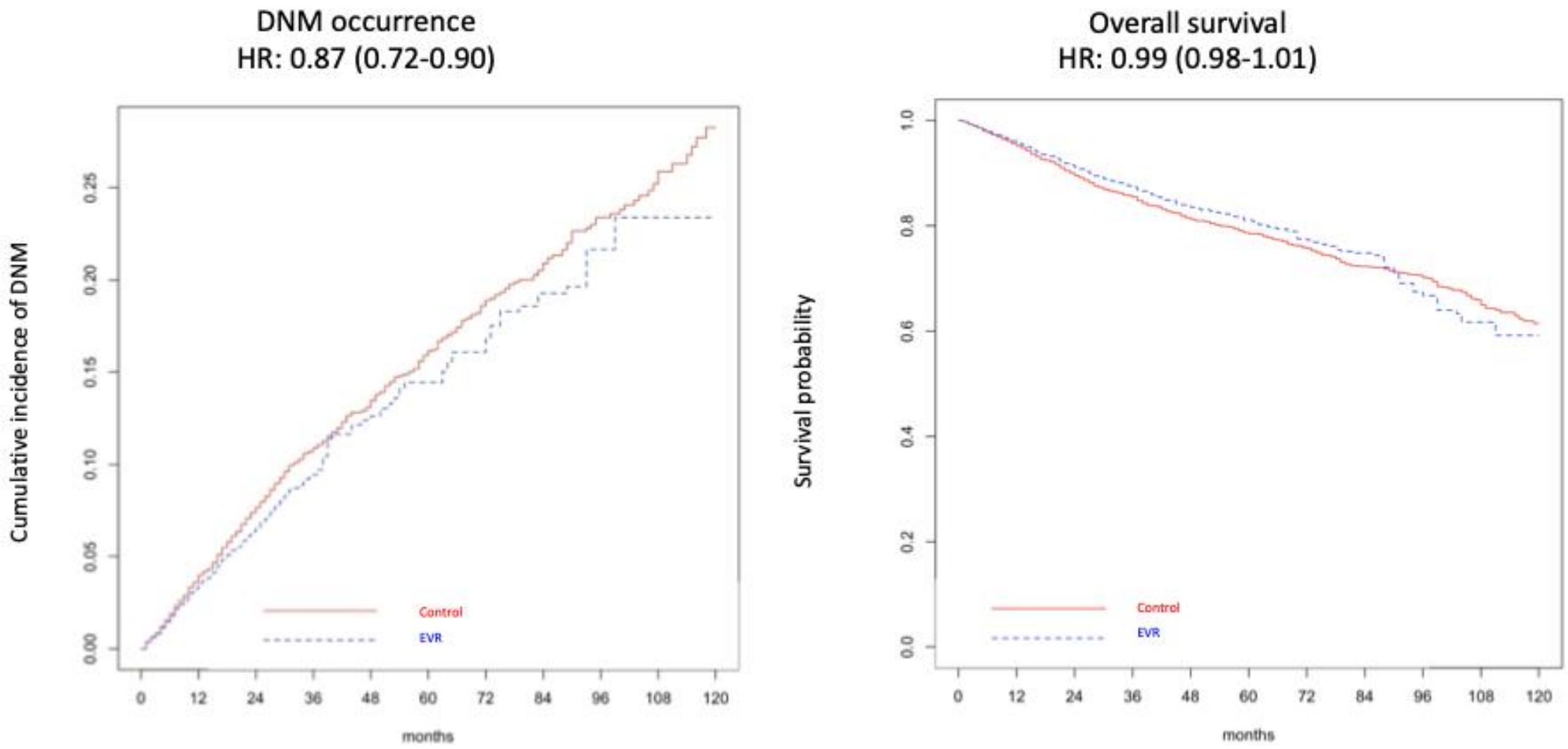

Supplementary tables

Table S1: Algorithm used to identify liver transplant recipients from the French national healthcare database.

Liver transplantation	
**CIM codes**	
Z944	Liver transplant [Orthotopic liver transplant]
(T864)	*(Liver transplant failure and rejection)*
**CCMP codes**	
HLEA002	Reduced liver transplantation
HLEA001	Total liver transplantation
HLQX008	Histopathological examination for oncological purposes of total hepatectomy specimens
**ATC codes**	
Reimbursement at least 3 months after hospital discharge after LT of one of the following:<br>L04AD02: Tacrolimus<br>L04AD01: Cyclosporine<br>L04AA06: Mycophenolic acid<br>L04AA18: Everolimus<br>And/or in association with one of the following:<br>L04AH01: Sirolimus<br>L04AX01: Azathioprine<br>H02AB06: Prednisolone<br>H02AB07: Prednisone	

Table S2: List of ICD-10 codes used to define cancer diagnoses for DNM classification.

ICD-10 Code	ICD label	Number of patients
**C22**	Hepatocellular carcinoma	3730
**C78**	Secondary malignant tumor of the lung	256
**C78**	Secondary malignant tumor of the liver and intrahepatic bile ducts	252
**C79**	Secondary malignant tumor of bone and bone marrow	240
**C34**	Malignant bronchus or lung tumor, unspecified	158
**C78**	Secondary malignant tumor of the retroperitoneum and peritoneum	156
**C44**	Malignant tumor of the skin of the face, other and unspecified parts	153
**C61**	Malignant prostate tumor	131
**C34**	Malignant tumor of the upper lobe, bronchi or lung	109
**C77**	Secondary and unspecified malignant tumor of the lymph nodes of the head,	92
**D46**	Refractory anemia, unspecified	84
**D47**	Monoclonal gammopathy of undetermined significance [GMSI]	82
**C77**	Secondary and unspecified malignant tumor of the intra-abdominal lymph nodes	76
**C78**	Secondary malignant tumor of the pleura	68
**C83**	Diffuse large B cell lymphoma	67
**C18**	Malignant colon tumor, unspecified	66
**C64**	Malignant tumor of the kidney, except the renal pelvis	64
**C67**	Malignant tumor of the bladder, unspecified	63
**C85**	Non-Hodgkin lymphoma, unspecified	55
**C34**	Malignant tumor of the lower lobe, bronchi or lung	51
**C32**	Malignant tumor of the larynx, unspecified	48
**C12**	Malignant tumor of the pyriform sinus	45
**D46**	Myelodysplastic syndrome, unspecified	45
**C20**	Malignant tumor of the rectum	44
**C15**	Malignant tumor of the esophagus, unspecified	43
**C50**	Malignant breast tumor, unspecified	43
**C01**	Malignant tumor of the base of the tongue	39
**C79**	Secondary malignant tumor of the brain and cerebral meninges	33
**C44**	Malignant tumor of the skin of the ear and external auditory canal	31
**C18**	Malignant tumor of the ascending colon	31
**C77**	Secondary and unspecified malignant tumor of the intrathoracic lymph nodes	27
**C10**	Malignant tumor of the oropharynx, unspecified	26
**C25**	Malignant tumor of the pancreas, unspecified	25
**C44**	Malignant tumor of the skin of the scalp and neck	24

**C44**	Malignant tumor of the skin of the upper limb, including the shoulder	24
**C79**	Secondary malignant tumor of other specific sites	24
**C02**	Malignant tumor of the tongue, unspecified	23
**C18**	Malignant tumor of the sigmoid colon	23
**C16**	Malignant stomach tumor, unspecified	22
**C85**	B-cell lymphomas, unspecified	22
**C32**	Malignant glottis tumor	22
**C32**	Malignant tumor of the supraglottic level	22
**C09**	Malignant tumor of the tonsil, unspecified	20
**D45**	Polycythemia vera	20
**C44**	Malignant skin tumor, unspecified	19
**C15**	Malignant tumor of the lower third of the esophagus	19
**C79**	Secondary malignant tumor of the adrenal gland	18
**C13**	Malignant tumor of the hypopharynx, unspecified	18
**C04**	Malignant tumor of the anterior floor of the mouth	18
**C44**	Malignant tumor of the skin of the trunk	16
**C25**	Malignant tumor of the pancreatic head	16
**C34**	Malignant tumor with contiguous locations of the bronchi and lung	16
**C10**	Malignant tumor of the lateral wall of the oropharynx	16
**C67**	Malignant tumor of the lateral wall of the bladder	16
**C26**	Malignant tumor of poorly defined sites of the digestive system	16
**C04**	Malignant tumor of the floor of the mouth, unspecified	15
**C02**	Malignant tumor of the point and lateral border of the tongue	15
**D47**	Myeloproliferative disease (chronic)	15
**C90**	Multiple myeloma	15
**C73**	Malignant thyroid tumor	15
**C15**	Malignant tumor of the middle third of the esophagus	14
**C83**	Small B cell lymphoma	13
**C83**	Lymphoma (diffuse) non-follicular, unspecified	13
**D46**	Other myelodysplastic syndromes	13
**C80**	Malignant tumor of unspecified primary site, as described	13
**C17**	Malignant tumor of the small intestine, unspecified	13
**C44**	Malignant tumor of the skin of the eyelid, including the canthus	12
**C10**	Malignant tumor with contiguous locations of the oropharynx	12
**C18**	Malignant tumor of the descending colon	12
**C80**	Malignant tumor of unspecified site	12
**C77**	Secondary, unspecified malignant tumor of a lymph node, unspecified	11
**C15**	Malignant tumor of the thoracic esophagus	11
**C32**	Malignant tumor with contiguous locations of the larynx	11
**C92**	Acute myeloblastic leukemia [AML]	11
**C78**	Secondary malignant tumor of the mediastinum	10
**C44**	Malignant tumor of the cutaneous surface of the lip	10

**D00**	Carcinoma in situ of the lip, oral cavity and pharynx	10
**C10**	Malignant tumor of the glossoepiglottic groove	10
**C18**	Malignant tumor of the cecum	10
**C50**	Malignant tumor of the supero-outer quadrant of the breast	10
**D47**	Essential (hemorrhagic) thrombocytopenia	10
**C79**	Secondary malignant tumor of the skin	9
**C44**	Malignant tumor of the skin of the lower limb, including the hip	9
**C83**	Other forms of diffuse non-Hodgkin lymphoma	9
**C34**	Malignant tumor of the main bronchus	9
**C15**	Malignant tumor of the cervical esophagus	9
**C09**	Malignant tumor with contiguous locations of the tonsil	9
**C07**	Malignant tumor of the parotid gland	9
**C04**	Malignant tumor of the lateral floor of the mouth	9
**C16**	Malignant tumor of the pyloric antrum	8
**C82**	Follicular lymphoma, unspecified	8
**C05**	Malignant tumor of the soft palate	8
**C19**	Malignant tumor of the rectosigmoid junction	8
**C18**	Malignant tumor of the transverse colon	8
**C18**	Malignant tumor of the left angle of the colon	8
**C71**	Malignant brain tumor, unspecified	8
**D46**	Refractory non-sideroblastic anemia, as specified	8
**D06**	Carcinoma in situ of the cervix, unspecified	7
**C53**	Malignant tumor of the cervix, unspecified	7
**C16**	Malignant tumor of the cardia	7
**C16**	Malignant tumor of the stomach body	7
**C25**	Malignant tumor of the endocrine pancreas	7
**C79**	Secondary malignant tumor of parts of the nervous system, other and unspecified	7
**C14**	Malignant tumor with contiguous locations of the lip, oral cavity and	7
**C21**	Malignant anal canal tumor	7
**C62**	Malignant tumor of a testicle, unspecified	6
**C46**	Kaposi sarcoma of the skin	6
**C38**	Malignant tumor of the pleura	6
**C56**	Malignant tumor of the ovary	6
**C85**	Other specific types of non-Hodgkin lymphoma	6
**C15**	Malignant tumor of the upper third of the esophagus	6
**C13**	Malignant tumor with contiguous locations of the hypopharynx	6
**C10**	Malignant tumor of the anterior surface of the epiglottis	6
**C09**	Malignant tumor of the tonsil pillar (anterior) (posterior)	6
**C06**	Malignant tumor of the mouth, unspecified	6
**C21**	Malignant tumor of the anus, unspecified	6
**D06**	Carcinoma in situ of the endocervix	5
**C53**	Malignant tumor of the endocervix	5
**C24**	Malignant tumor of the ampulla of Vater	5

**C78**	Secondary malignant tumor of the digestive organs, other and unspecified	5
**C83**	Burkitt lymphoma	5
**C81**	Hodgkin's lymphoma, unspecified	5
**C34**	Malignant tumor of the middle lobe, bronchi or lung	5
**C14**	Malignant tumor of the pharynx, unspecified	5
**C13**	Malignant tumor of the posterior wall of the hypopharynx	5
**C10**	Malignant tumor of the posterior wall of the oropharynx	5
**C05**	Malignant tumor of the palate, unspecified	5
**C04**	Malignant tumor with contiguous locations in the floor of the mouth	5
**C02**	Malignant tumor with contiguous locations of the tongue	5
**C18**	Malignant tumor of the right angle of the colon	5
**C91**	B-cell chronic lymphocytic leukemia	5
**C79**	Secondary malignant tumor of the bladder and urinary organs, other and not p	5
**C16**	Malignant tumor of the greater curvature of the stomach, unspecified	4
**C25**	Malignant tumor of the tail of the pancreas	4
**C25**	Malignant tumor of the pancreatic duct	4
**C84**	T-cell lymphomas, other and unspecified	4
**C41**	Malignant tumor of the bones of the skull and face	4
**C41**	Malignant tumor of the mandible	4
**C15**	Malignant tumor of the abdominal esophagus	4
**C15**	Malignant tumor with contiguous locations of the esophagus	4
**C13**	Malignant tumor of the aryepiglottic fold (hypopharyngeal side)	4
**C11**	Malignant tumor of the nasopharynx, unspecified	4
**C09**	Malignant tumor of the tonsillar fossa	4
**C02**	Malignant tumor of the dorsal surface of the tongue	4
**C02**	Malignant tumor of the anterior two-thirds of the tongue, unspecified	4
**C18**	Malignant tumor of the appendix	4
**C50**	Malignant tumor of the central part of the breast	4
**D46**	Refractory sideroblastic anemia	4
**D46**	Refractory anemia with excess blasts [AREB]	4
**C95**	Leukemia, unspecified	4
**C88**	Waldenstrom macroglobulinemia	4
**C67**	Malignant tumor with contiguous locations of the bladder	4
**C54**	Malignant endometrial tumor	3
**C62**	Malignant tumor of the descended testicle	3
**C16**	Malignant fundus tumor	3
**C84**	Mycosis fungoides	3
**C43**	Malignant melanoma of the face, other and unspecified parts	3
**C43**	Malignant melanoma of the lower limb, including the hip	3
**C43**	Melanoma (malignant) of the skin, unspecified	3

**C45**	Pleural mesothelioma	3
**C25**	Malignant tumor of the body of the pancreas	3
**C77**	Secondary and unspecified malignant tumor of the intrapelvic lymph nodes	3
**C88**	B-cell marginal zone lymphoma of mu-associated lymphoid tissues	3
**C82**	Follicular lymphoma, grade II	3
**C06**	Malignant tumor of the retromolar region	3
**C03**	Malignant tumor of the upper gum	3
**C03**	Malignant tumor of the lower gum	3
**C03**	Malignant tumor of the gum, unspecified	3
**C02**	Malignant tumor of a lingual tonsil	3
**C18**	Malignant tumor with contiguous locations of the colon	3
**C50**	Malignant tumor of the upper inner quadrant of the breast	3
**C50**	Malignant tumor with contiguous locations of the breast	3
**C71**	Malignant tumor of the brain, except lobes and ventricles	3
**D47**	Osteomyelofibrosis	3
**C95**	Acute unspecified cell leukemia	3
**C92**	Chronic myeloid leukemia [CML] ABL-BCR positive	3
**C67**	Malignant tumor of the dome of the bladder	3
**C74**	Malignant adrenal tumor, unspecified	3
**C66**	Malignant tumor of the ureter	3
**C49**	Malignant tumor of the connective tissue and other soft tissues of the upper limb	3
**C46**	Multiple organ Kaposi sarcoma	3
**C45**	Mesothelioma of the peritoneum	3
**C17**	Malignant tumor of the duodenum	3
**C44**	Malignant tumor with contiguous locations of the skin	2
**C25**	Malignant tumor of other parts of the pancreas	2
**C78**	Secondary malignant tumor of the respiratory organs, other and unspecified	2
**C78**	Secondary malignant tumor of the small intestine	2
**C77**	Secondary and unspecified malignant tumor of the inguinal lymph nodes	2
**C77**	Secondary and unspecified malignant tumor of the lymph nodes of the m sites	2
**D47**	Other specified tumors with unpredictable and unknown evolution of lymphoid tissues	2
**C84**	Peripheral T-cell lymphoma, not elsewhere classified	2
**C84**	Cutaneous T-cell lymphoma, unspecified	2
**C83**	Mantle cell lymphoma	2
**C82**	Follicular lymphoma, grade I	2
**C82**	Follicular lymphoma, grade III, unspecified	2
**B21**	HIV disease causes other malignant tumors of lymphoid tissues, hematologic	2

**C00**	Malignant tumor of the lower lip, (free edge)	2
**C79**	Secondary malignant tumor of the kidney and renal pelvis	2
**H60**	Malignant external otitis	2
**C76**	Malignant tumor of ill-defined location of the head, face and neck	2
**C32**	Malignant tumor of the subglottic level	2
**C11**	Malignant tumor of the upper wall of the nasopharynx	2
**C11**	Malignant tumor of the posterior wall of the nasopharynx	2
**C11**	Malignant tumor with contiguous locations of the nasopharynx	2
**C06**	Malignant tumor of the cheek mucosa	2
**C05**	Malignant tumor of the uvula	2
**C78**	Secondary malignant tumor of the large intestine and rectum	2
**C21**	Malignant tumor with contiguous locations of the rectum, anus and anal canal	2
**C71**	Malignant frontal lobe tumor	2
**C70**	Malignant tumor of the cerebral meninges	2
**D47**	Chronic eosinophilic leukemia [hypereosinophilic syndrome]	2
**D46**	Refractory anemia with dysplasia of multiple cell lines	2
**C90**	Extramedullary plasmacytoma	2
**C67**	Malignant trigone tumor of the bladder	2
**C67**	Malignant tumor of the anterior wall of the bladder	2
**C76**	Malignant tumor of ill-defined location of the pelvis	2
**C67**	Malignant tumor of the ureteral orifice	2
**C49**	Malignant tumor of the connective tissue and other soft tissues of the head, f	2
**C49**	Malignant tumor of the connective tissue and other soft tissues of the lower limb	2
**C49**	Malignant tumor with contiguous locations of connective tissue and other tissues	2
**C48**	Malignant tumor with contiguous locations of the retroperitoneum and peritoneum	2
**C46**	Soft tissue Kaposi sarcoma	2
**C46**	Kaposi's sarcoma of lymph nodes	2
**C46**	Kaposi's sarcoma, unspecified	2
**C41**	Malignant tumor of the spine	2
**C41**	Malignant tumor of the ribs, sternum and clavicle	2
**C41**	Malignant tumor of the bones of the pelvis, sacrum and coccyx	2
**C17**	Malignant tumor of the jejunum	2
**C17**	Malignant tumor of the ileum	2
**B21**	HIV disease causing a malignant tumor, unspecified	2
**D06**	Carcinoma in situ of other parts of the cervix	1
**C57**	Malignant tumor of the adnexa of the uterus, unspecified	1
**C54**	Malignant tumor with contiguous locations of the body of the uterus	1
**C53**	Malignant tumor of the ectocervix	1

**C53**	Malignant tumor with contiguous locations of the cervix of the uterus	1
**C63**	Malignant spermatic cord tumor	1
**C62**	Malignant tumor of the ectopic testicle	1
**D00**	Carcinoma in situ of the stomach	1
**C16**	Malignant tumor of the lesser curvature of the stomach, unspecified	1
**C16**	Malignant tumor with contiguous locations of the stomach	1
**D04**	Carcinoma in situ of the skin of the trunk	1
**C43**	Malignant melanoma of the ear and external auditory canal	1
**C43**	Malignant melanoma of the scalp and neck	1
**C43**	Malignant melanoma of the trunk	1
**C25**	Malignant tumor with contiguous locations of the pancreas	1
**C77**	Secondary, unspecified malignant tumor of the lymph nodes of the armpit	1
**C91**	Adult T-cell lymphoma/leukemia (HTLV-1 associated)	1
**C86**	T-cell (intestinal) lymphoma, enteropathic type	1
**C86**	Angioimmunoblastic T-cell lymphoma	1
**C85**	Mediastinal (thymic) large B-cell lymphoma	1
**C84**	ALK-positive anaplastic large cell lymphoma	1
**C83**	Immunoblastic lymphoma (diffuse)	1
**C83**	Lymphoblastic lymphoma (diffuse)	1
**C83**	Undifferentiated lymphoma (diffuse)	1
**C82**	Follicular lymphoma, grade IIIa	1
**C82**	Follicular lymphoma, grade IIIb	1
**C82**	Other forms of follicular lymphoma	1
**C81**	Hodgkin's lymphoma, predominantly lymphocytic, nodular form	1
**C81**	Hodgkin's lymphoma (classic), rich in lymphocytes	1
**B21**	HIV disease causes Burkitt lymphoma	1
**B21**	HIV disease causes other non-Hodgkin's lymphomas	1
**C00**	Malignant tumor of the corner of the lips	1
**C38**	Malignant tumor of the heart	1
**C69**	Malignant tumor of the orbit	1
**C69**	Malignant tumor with contiguous locations of the eye and its adnexa	1
**C32**	Malignant tumor of the laryngeal cartilage	1
**C31**	Malignant tumor with contiguous locations in the sinuses of the face	1
**C31**	Malignant tumor of the facial sinuses, unspecified	1
**C11**	Malignant tumor of the lateral wall of the nasopharynx	1
**C08**	Malignant tumor of the submandibular gland	1
**C06**	Malignant tumor of the vestibule of the mouth	1
**C06**	Malignant tumor with contiguous locations of the mouth, other parts and not pre	1
**C05**	Malignant tumor of the palatal vault	1

**C05**	Malignant tumor with contiguous locations on the palate	1
**D01**	Carcinoma in situ of the rectosigmoid junction	1
**D05**	Other carcinomas in situ of the breast	1
**C50**	Malignant tumor of the lower outer quadrant of the breast	1
**C50**	Malignant tumor of the axillary extension of the breast	1
**C50**	Malignant tumor of the nipple and areola	1
**C71**	Malignant tumor of the occipital lobe	1
**C71**	Malignant tumor with contiguous locations of the brain	1
**C70**	Malignant tumor of the meninges, unspecified	1
**C94**	Acute erythroid leukemia	1
**C94**	Acute panmyelosis with myelofibrosis	1
**C94**	Myelodysplastic and myeloproliferative disease, not elsewhere classified	1
**C93**	Acute monoblastic/monocytic leukemia	1
**C92**	Other myeloid leukemias	1
**C92**	Acute myeloid leukemia with dysplasia of several cell lines	1
**C92**	Myeloid leukemia, unspecified	1
**C91**	Hairy cell leukemia	1
**C91**	Other lymphoid leukemias	1
**C91**	Lymphocytic leukemia, unspecified	1
**C90**	Solitary plasmacytoma	1
**C88**	Other malignant immunoproliferative diseases	1
**C88**	Immunoproliferative (malignant) disease, unspecified	1
**C86**	Primary cutaneous lymphoproliferative syndrome with CD30-positive T cells	1
**C68**	Malignant tumor of the urethra	1
**C67**	Malignant tumor of the posterior wall of the bladder	1
**C96**	Multifocal and multisystem Langerhans cell histiocytosis (disseminated	1
**C96**	Multifocal and unisystemic Langerhans cell histiocytosis	1
**C96**	Unifocal Langerhans cell histiocytosis	1
**C96**	Histiocytic sarcoma	1
**C76**	Malignant tumor of ill-defined location of the thorax	1
**C76**	Malignant tumor of ill-defined location of the abdomen	1
**C76**	Malignant tumor of poorly defined site of a lower limb	1
**C74**	Malignant tumor of the adrenal medulla	1
**C72**	Malignant tumor with contiguous locations of the brain and other parts of the brain	1
**C72**	Malignant tumor of the (central) nervous system, unspecified	1
**C69**	Malignant choroidal tumor	1
**C69**	Malignant tumor of the lacrimal gland and lacrimal duct	1
**C68**	Malignant tumor of a urinary organ, unspecified	1
**C60**	Malignant tumor of the foreskin	1
**C60**	Malignant glans tumor	1
**C60**	Malignant tumor of the penis, unspecified	1

**C57**	Malignant tumor with contiguous locations of the female genital organs	1
**C57**	Malignant tumor of a female genital organ, unspecified	1
**C52**	Malignant tumor of the vagina	1
**C51**	Malignant tumor of the vulva, unspecified	1
**C49**	Malignant tumor of the connective tissue and other soft tissues of the trunk, unspecified	1
**C49**	Malignant tumor of connective tissue and other soft tissues, unspecified	1
**C48**	Malignant tumor of the retroperitoneum	1
**C48**	Malignant peritoneal tumor, unspecified	1
**C46**	Pulmonary Kaposi sarcoma	1
**C46**	Kaposi's sarcoma of other locations	1
**C45**	Mesothelioma, unspecified	1
**C41**	Malignant tumor with contiguous locations of bones and articular cartilage	1
**C40**	Malignant tumor of the scapula and long bones of the upper limb	1
**C40**	Malignant tumor of the short bones of the upper limb	1
**C40**	Malignant tumor of the bones and articular cartilage of a limb, unspecified	1
**C39**	Malignant tumor of the upper respiratory tract, part unspecified	1
**C39**	Malignant tumor with contiguous locations of the respiratory and intrathorax organs	1
**C38**	Malignant tumor of the posterior mediastinum	1
**C37**	Malignant tumor of the thymus	1
**C26**	Malignant tumor of the spleen	1
**C17**	Malignant tumor with contiguous locations of the small intestine	1
**B21**	HIV disease causing Kaposi's sarcoma	1

Table S3: CCMP procedure codes used to confirm the diagnosis of DNM

Code	Procedure	Number of patients
**HHFE006**	Rectocolic mucosectomy session, by endoscopy	441
**HHFE004**	Excision of a polyp of more than 1cm in diameter or of 4 or more polyps of the colon	440
**ZZMP015**	Creation of a personalized [individualized] restraint system for radiothesis	245
**ZZML002**	Control of the dose of external irradiation received [In vivo dosimetry]	222
**FCQX006**	Pathological examination with an oncological purpose of a group's surgical specimen	207
**HHQX004**	Pathological examination for oncological purposes of endoscopic surgical specimen	201
**ZZMK018**	Preparation for external irradiation with identification by CT scan, dosimetry	170
**ZZNL063**	External irradiation session by linear accelerator of equal or greater power	170
**YYYY128**	Supplement for setting up a multi-leaf collimator	157
**HLQX005**	Pathological examination for oncological purposes of specimens from several hepatectomies	156
**FCQX010**	Pathological examination for oncological purposes of 2 or 3 excised specimen	146
**PDFA001**	Excision of fascial and/or subfascial lesion of soft tissues	133
**ZZML001**	Control of the centering of external irradiation fields	132
**ZZML003**	Control of the centering of external irradiation fields at the center	122
**QZFA036**	Excision of superficial lesion of the skin by excision of a cutaneous area	118
**QZQX004**	Pathological examination for oncological purposes of a specimen of skin excision	111
**ZZNL051**	External irradiation session by dedicated machine producing photons	91
**ZZNL054**	External irradiation session by linear accelerator with intensity modulation	89
**ZZMP016**	Preparation for external irradiation in stereotaxic conditions without synchronization	87
**ZZNL060**	External irradiation session in stereotaxic conditions by dedicated professional machine	87
**HFQX005**	Pathological examination for oncological purposes of endoscopic surgical specimen	85
**JDQE001**	Urethrovesical fibroscopy	84
**ZZNL065**	External irradiation session by linear accelerator of equal or greater power	84

**QZFA004**	Excision of superficial lesion of the skin	74
**HLNC003**	Destruction of hepatic tumor with radiofrequency current	67
**YYYY026**	Supplement for mediastinal lymph node dissection during pneumonectomy	65
**HGFA005**	Single segmental resection of the small intestine for obstruction, by laparotomy	62
**JGHJ001**	Prostate biopsy, transrectally with ultrasound guidance	60
**FCQX012**	Pathological examination for oncological purposes of surgical specimens weighing 4 to 6 g	59
**JDFE001**	Resection of 4 or more bladder tumors, by endoscopy	58
**GFFA013**	Pulmonary lobectomy, by thoracotomy	53
**HLNA007**	Destruction of hepatic tumor with radiofrequency current, by laparotomy	50
**QZQX032**	Pathological examination for oncological purposes of several surgical specimens	50
**QZQX005**	Pathological examination for oncological purposes of skin and/or excision specimen	49
**17 FCFA**	Lymphonodal dissection of the hilum of the lung and the mediastinum, during an intervention	48
**JDFE002**	Resection of 1 to 3 bladder tumors, by endoscopy	45
**GFFC002**	Non-anatomic partial excision of the lung, by thoracoscopy	44
**ZZNL059**	External irradiation session in stereotaxic conditions by dedicated professional machine	44
**GFQX004**	Pathological examination for oncological purposes of a excised specimen from lobectomy	42
**HHFA009**	Right colectomy with restoration of continuity, by laparotomy	41
**HGQX005**	Pathological examination for oncological purposes of endoscopic surgical specimen	40
**HHFA026**	Right colectomy without restoration of continuity, by laparotomy	37
**JKFA031**	Conization of the cervix	37
**LBFA023**	Excision of lesion of the maxillary bone and/or body of the mandible	37
**QZEA031**	Total skin autograft on a surface location less than 10 cm_	37
**YYYY166**	Supplement for quality ballistic controls in p-imaging radiotherapy	37
**GFQX006**	Pathological examination for oncological purposes of pulmonary excision specimen	36
**HMQX004**	Pathological examination for oncological purposes of duct excision specimen	36
**HHQX008**	Pathological examination for oncological purposes of endoscopic surgical specimen	35
**HPQX005**	Pathological examination for oncological purposes of a tumor excision specimen	35

**HHQX006**	Pathological examination for oncological purposes of partial colectomy specimen	34
**QZFA007**	Excision of subcutaneous suprafascial lesion from 3 cm to 10 cm long axis	34
**HHFA021**	Total colectomy with preservation of the rectum, without restoration of continuity	33
**KCFA005**	Total thyroidectomy, by cervicotomy	32
**ZZNL050**	External irradiation session by linear accelerator with intensity modulation	32
**GFFA009**	Pulmonary lobectomy, by thoracotomy with preparation by thoracoscopy	29
**JGFC001**	Total vesiculoprostatectomy, by laroscopy	29
**HAQX017**	Pathological examination for oncological purposes of glossectomy specimen	27
**16 FCFA**	Unilateral partial cervical lymph node dissection, by cervicotomy	27
**GDFE004**	Excision of lesion or peeling of the mucosa of the glottic plane, by laryngoscopy	27
**FCQX011**	Pathological examination for oncological purposes of surgical specimens from 7 groups	25
**GFFA017**	Single non-anatomic partial excision of the lung, by thoracotomy	24
**JAQX005**	Pathological examination for oncological purposes of partial nephrectomy specimen	23
**QZFA002**	Excision of a subcutaneous suprafascial lesion of less than 3 cm in long axis	23
**JGFA006**	Total vesiculoprostatectomy, by laparotomy	22
**LAFA021**	Anterior ethmoidectomy, by endoscopy	22
**HNFA007**	Cephalic duodenopancreatectomy, by laparotomy	21
**HPFA004**	Resection of the greater omentum, by laparotomy	21
**GFFA029**	Single or multiple lung segmentectomy, by thoracotomy	20
**JKQX007**	Pathological examination for oncological purposes of cervical conization specimen	20
**JGQX004**	Pathological examination for oncological purposes of a vesiculoprostatectomy specimen	19
**JAFC005**	Partial nephrectomy, by laposcopy or retroperitoneoscopy	19
**LFFA001**	Bilateral total lumbar or lumbosacral laminarthrectomy, via posterior approach	19
**ZZMK020**	Resumption of preparation for external irradiation with identification by CT scan	19
**HKFA008**	Destruction and/or excision of non-tumorous superficial lesion of the anus	18
**KEFA002**	Partial or total adrenalectomy, by direct approach	18
**EPPP002**	Clearing obstruction of implantable site or tunneled central venous line by	17

**HGQX008**	Pathological examination for oncological purposes of segmental excision specimen	17
**KEQX004**	Pathological examination for oncological purposes of adrenalectomy specimen	16
**HEQX005**	Pathological examination for oncological purposes of endoscopic surgical specimen	16
**YYYY047**	Irradiation of less than 300 cm_ in fixed fields by accelerator by fraction of	16
**HHFA014**	Left colectomy without release of the left colic angle, without restoration	15
**HHFA006**	Left colectomy with release of the left colic angle, with restoration	15
**JAFA008**	Partial nephrectomy with dissection of the vascular pedicle, by lobectomy	15
**HHFA018**	Transverse colectomy, by laparotomy	14
**ZZNL052**	External irradiation session in stereotaxic conditions by machine producing	14
**GDFE003**	Laryngeal cordectomy, by direct laryngoscopy with laser	13
**HDFA002**	Lateral oropharyngectomy with tonsillectomy, via intraoral approach	13
**GFFA021**	Multiple non-anatomic partial excisions of the lung, by thoracotomy	13
**FAFA014**	Tonsillectomy by dissection	13
**GAFA005**	Transfixing excision of lesion of the skin of the nose and/or the nasal mucosa	13
**HFFA006**	Partial lower gastrectomy with gastrojejunal anastomosis, by laparotomy	12
**HAFA023**	Partial glossectomy of mobile tongue, via intraoral approach	12
**JHFA005**	Orchiectomy, via inguinal approach	12
**YYYY152**	Irradiation of less than 300 cm_ in fixed fields by accelerator by fraction of	12
**HFQX004**	Pathological examination for oncological purposes of gastrectomy specimen	11
**HAQX005**	Pathological examination for oncological purposes of excised specimen of lip	11
**HAQX004**	Pathological examination for oncological purposes of pelvimandibulectomy piece	11
**HCQX004**	Pathological examination for oncological purposes of gland excision specimen	11
**JGQX005**	Pathological examination for oncological purposes of a specimen of vesiculo-prostato-cystectomy	11
**JAQX004**	Pathological examination for oncological purposes of total nephrectomy specimen	11
**QEQX004**	Pathological examination for oncological purposes of a mastectomy specimen	11

**JKFA026**	Total hysterectomy, vaginal approach	11
**AZQX005**	Pathological examination for oncological purposes of a tumor excision specimen	11
**GGQX004**	Pathological examination for oncological purposes of a tumor excision specimen	11
**HAFA006**	Mobile tongue pelviglossectomy, via intraoral approach	11
**PAQX004**	Pathological examination for oncological purposes of a tumor excision specimen	11
**YYYY049**	Irradiation of less than 300 cm_ in fixed fields by accelerator	11
**HHFA024**	Left colectomy with release of the left colic angle, without restoration	10
**GCQX004**	Pathological examination for oncological purposes of cavity excised specimen	10
**GDQX008**	Pathological examination for oncological purposes of laryngectomy specimen	10
**LAFA018**	Bilateral total ethmoidectomy with sphenoidotomy, by endoscopy	10
**FFQX005**	Pathological examination for oncological purposes of splenectomy specimen	10
**JKFA028**	Total hysterectomy with unilateral or bilateral adnexectomy, by laparotomy	10
**JHFA017**	Orchiectomy, via scrotal approach	10
**KCFA008**	Unilateral lobo-isthmectomy of the thyroid gland, by cervicotomy	10
**AAFA002**	Excision of intraparenchymal brain tumor, by craniotomy	9
**HHFA002**	Left colectomy with release of the left colic angle, with restoration	9
**HCFA008**	Partial parotidectomy with dissection and preservation of the facial nerve	9
**GFQX009**	Pathological examination for oncological purposes of pulmonary excision specimen	9
**FAFA015**	Tonsillectomy by dissection, with adenoidectomy	9
**HAFA007**	Partial oral pelvectomy, via intraoral approach	9
**PDQX005**	Pathological examination for oncological purposes of a tumor excision specimen	9
**ZZNL062**	External irradiation session by linear accelerator of equal or greater power	9
**YYYY197**	Irradiation of less than 300 cm_ in fixed fields by accelerator	9
**HHFA008**	Right colectomy with restoration of continuity, by laparoscopy	8
**HHFA030**	Total coloproctectomy without restoration of continuity, by laparotomy	8
**GDQX005**	Pathological examination for oncological purposes of laryngectomy specimen	8

**GDNE003**	Destruction of laryngeal lesion by direct laryngoscopy with laser	8
**HCFA009**	Total parotidectomy with dissection and preservation of the facial nerve	8
**HDFA005**	Total pharyngolaryngectomy	8
**KCNL003**	Internal irradiation of the thyroid gland by administration of iodine 131 at a dose	8
**27 FCFA**	Complete unilateral cervical lymph node dissection, extended to the muscles	8
**LAFA022**	Bilateral total ethmoidectomy, by endoscopy	8
**GHFA004**	Excision of mediastinal tumor, by thoracotomy	7
**ZAQA001**	Exploration of the neck, by cervicotomy	7
**28 FCFA**	Excision of lymph node [node] of the neck for therapeutic purposes, by cervicotomy	7
**GDQX004**	Pathological examination for oncological purposes of laryngeal cordectomy specimen	7
**GCFD001**	Excision of nasopharynx lesion, nasally and/or orally	7
**HAND004**	Destruction of lesion of the mucous membrane of the mouth or oropharynx	7
**HAFA004**	Interrupting pelvimandibulectomy, via cervicofacial approach	7
**JGNL001**	Prostate brachytherapy by permanent insertion of iodine 125	7
**KCQX004**	Pathological examination for oncological purposes of subthyroidectomy specimen	7
**JKND002**	Destruction of lesion of the cervix, vagina, vulva, perineum	7
**JDNE001**	Destruction of bladder lesion, by endoscopy	7
**ZZNL058**	External irradiation session in stereotaxic conditions by machine producing	7
**HKQX005**	Pathological examination for oncological purposes of an excision specimen of the anus canal	6
**HHQX007**	Pathological examination for oncological purposes of appendectomy specimen	6
**HHFA022**	Total colectomy with preservation of the rectum, with ileorectal anastomosis	6
**HJFA002**	Rectosigmoid resection with infraperitoneal colorectal anastomosis	6
**HJFD005**	Resection of the rectal mucosa with anterior hemicircumferential plication	6
**JJFA004**	Salpingoovariectomy [Annexectomy], by laparotomy	6
**HNQX006**	Pathological examination for oncological purposes of a specimen of duodenopancreatectomy	6
**KCQX005**	Pathological examination for oncological purposes of lobectomy specimen	6
**BZQX004**	Pathological examination for oncological purposes of an excerpted organ specimen	6
**FEFF002**	Collection of blood hematopoietic stem cells by cytapheresis	6

**LAFA024**	Unilateral total ethmoidectomy, by endoscopy	6
**LAFA017**	Unilateral total ethmoidectomy with sphenoidotomy, by endoscopy	6
**JAFC006**	Unilateral total nephrectomy, by laparoscopic or retroperitoneoscopy	6
**JAFA023**	Unilateral total nephrectomy, by laparotomy	6
**JAFA009**	Total nephrectomy extended to the renal compartment, by laparotomy or lumbosacral approach	6
**YYYY048**	Irradiation of less than 300 cm_ in fixed fields by accelerator	6
**AFLB003**	Intrathecal injection session of anticancer pharmacological agent	5
**HHQX010**	Pathological examination for oncological purposes of total colectomy specimen	5
**HHFA017**	Left colectomy without release of the left colic angle, with restoration	5
**HHFA010**	Left colectomy without release of the left colic angle, with restoration	5
**HHFA005**	Total colectomy with preservation of the rectum, without restoration of continuity	5
**HHFA031**	Total coloproctectomy with ileoanal anastomosis, by laparotomy	5
**HJFA004**	Rectosigmoid resection with infraperitoneal colorectal anastomosis,	5
**HJFA006**	Rectosigmoid resection by laparotomy, with coloanal anastomosis	5
**GDNE002**	Destruction of laryngeal lesion, by direct laryngoscopy without laser	5
**GDFA005**	Total laryngectomy	5
**HDQX007**	Pathological examination for oncological purposes of a pharyngolaryngectal specimen	5
**HDFA019**	Oropharyngectomy by mandibulotomy	5
**QEQX008**	Pathological examination for oncological purposes of a mastectomy specimen	5
**KDQX005**	Pathological examination for oncological purposes of parathyroidectomy specimen	5
**JKQX005**	Pathological examination for oncological purposes of hysterectomy specimen	5
**JDFA008**	Total cystectomy with transintestinal cutaneous ureterostomy by loop	5
**JHFA008**	Orchidectomy extended to the spermatic cord [Orchiepididymectomy], by inward approach	5
**KEFC002**	Partial or total adrenalectomy, by laparoscopy or retroperitoneoscopy	5
**ZZNL053**	External irradiation session by linear accelerator of equal or greater power	5

**YYYY211**	Irradiation of less than 300 cm_ in fixed fields by accelerator by fraction	5
**YYYY459**	Irradiation of 300 cm_ and more in fixed fields by accelerator per fraction	5
**YYYY109**	Supplement for using custom and focused caches	5
**HHQX011**	Pathological examination for oncological purposes of coloprotectectomy specimen	4
**HJQX004**	Pathological examination for oncological purposes of rectosigmoidectomy	4
**HFFA005**	Total gastrectomy with restoration of continuity, by laparotomy	4
**FAQX004**	Pathological examination for oncological purposes of tonsillectomy specimen	4
**LAFA019**	Bilateral total ethmoidectomy with nasal septoplasty and sphenoidotomy	4
**GDFA008**	Extended supraglottic laryngectomy at the base of the tongue	4
**HCFA011**	Submandibular gland excision	4
**HDFA008**	Lateral oropharyngectomy, by cervicotomy	4
**HDFA009**	Posterior oropharyngectomy, via intraoral approach	4
**HDFA016**	Lateral pharyngectomy, by cervicotomy	4
**JJFC009**	Bilateral oophorectomy, by laparoscopy	4
**HNFA013**	Left pancreatectomy with splenectomy	4
**JGNJ900**	Destruction of prostate lesions by high intensity focused ultrasound,	4
**JHQX005**	Pathological examination for oncological purposes of total orchiectomy specimen	4
**KCNL004**	Internal irradiation of the thyroid gland by administration of iodine 131 at a dose	4
**LAFA015**	Unilateral total ethmoidectomy with nasal septoplasty and sphenoidotomy	4
**HPFC002**	Resection of the greater omentum, by laparoscopy	4
**JHFA006**	Orchiectomy with testicular prosthesis placement, via inguinal approach	4
**LAFA012**	Shaping resection of the middle level of the face, via intraoral approach	4
**YYYY056**	Irradiation in cyclotherapy by accelerator by fraction of 2 Grays	4
**ZANL001**	External intracranial irradiation in stereotaxic conditions with frightening frame	3
**HHFA004**	Total colectomy with preservation of the rectum, with ileorectal anastomosis	3
**HHFA028**	Total coloproctectomy with ileoanal anastomosis, by laparoscopy or laparotitis	3
**HJFC023**	Secondary proctectomy by laparoscopy with ileoanal anastomosis	3

**JFQX004**	Pathological examination for oncological purposes of total pelvectomy specimen	3
**HFQX008**	Pathological examination for oncological purposes of total gastrectomy specimen	3
**FBQX004**	Pathological examination for oncological purposes of a thymus excision specimen	3
**HEQX004**	Pathological examination for oncological purposes of ophagectomy specimen	3
**GDFE007**	Laryngeal cordectomy, by direct laryngoscopy without laser	3
**HEQX008**	Pathological examination for oncological purposes of ophagopharyngeal specimen	3
**HAND005**	Destruction of lesion of the mucous membrane of the mouth or oropharynx	3
**HDFA011**	Oropharyngectomy with interruptive partial mandibulectomy, via cervical approach	3
**GFQX008**	Pathological examination for oncological purposes of pneumonectomy specimen	3
**GFQX005**	Pathological examination for oncological purposes of a lobectomy specimen	3
**GFFA024**	Pneumonectomy, by thoracotomy	3
**JKND003**	Destruction of lesion of the cervix by colposcopy, with laser	3
**JKFC005**	Total hysterectomy, by laparoscopy	3
**JKFC003**	Total hysterectomy with unilateral or bilateral adnexectomy, by fluoroscopy	3
**JKNL002**	Intracavitary uterovaginal brachytherapy at pulsed dose rate, with dosimetry	3
**JDQX005**	Pathological examination for oncological purposes of cystectomy specimen	3
**JDFA025**	Total vesiculo-prostato-cystectomy with transintestinal cutaneous ureterostomy	3
**FAFA006**	Tonsillectomy by dissection, with adenoidectomy and unilateral myringotomy o	3
**LAFA023**	Unilateral anterior ethmoidectomy with nasal septoplasty, by endoscopy	3
**HAFA017**	Interrupting pelvi-glosso-mandibulectomy, via cervicofacial approach	3
**JAFA024**	Partial nephrectomy without dissection of the vascular pedicle, by lombotomy or	3
**KCFA001**	Unilateral lobectomy of the thyroid gland, by cervicotomy	3
**ZZMP012**	Preparation for intracranial irradiation in stereotaxic conditions on the back	3
**ZZNL048**	External irradiation session by linear accelerator of equal or greater power	3
**YYYY151**	Supplement for quality ballistic controls in radiotherapy by gammagraph	3

**HFFA003**	Partial upper gastrectomy [upper polar] with restoration of the	2
**HFFA002**	Partial lower gastrectomy with gastroduodenal anastomosis, by laparotomy	2
**HFFC002**	Lower partial gastrectomy with gastrojejunal anastomosis, by laparoscopic	2
**JJQX004**	Pathological examination for oncological purposes of oophorectomy specimen	2
**JJFC008**	Unilateral oophorectomy, by laparoscopy	2
**HNQX007**	Pathological examination for oncological purposes of pancreatectomy specimen	2
**HNFA005**	Excision of pancreatic tumor, by laparotomy	2
**GFQX010**	Pathological examination for oncological purposes of pulmonary excision specimen	2
**GFFA004**	Pulmonary lobectomy with resection-anastomosis or bronchus re-implantation	2
**GFFA027**	Pulmonary lobectomy with resection of the chest wall, by thoracotomy	2
**GFFA022**	Pulmonary lobectomy with resection of organ and/or neighboring structure,	2
**JKFA025**	Total hysterectomy with anterior or posterior colpoperineorrhaphy	2
**JKFA002**	Total hysterectomy with anterior and posterior colpoperineorrhaphy	2
**JDFA023**	Total vesiculo-prostato-cystectomy with cutaneous ureterostomy, by laparotomy	2
**LAFA016**	Unilateral total ethmoidectomy with nasal septoplasty, by endoscopy	2
**LAFA020**	Bilateral total ethmoidectomy with nasal septoplasty, by endoscopy	2
**GDFA006**	Vertical partial laryngectomy	2
**GDFA016**	Anterior frontal laryngectomy with epiglottoplasty	2
**GDFA013**	Hemilaryngopharyngectomy	2
**GDFA018**	Total laryngectomy extended to the base of the tongue	2
**GGLB001**	Intrapleural injection session of anticancer pharmacological agent	2
**HNQX004**	Pathological examination for oncological purposes of papil specimen	2
**HAND002**	Destruction of lesion of the mucous membrane of the mouth or oropharynx	2
**HAND001**	Destruction of lesion of the mucous membrane of the mouth or oropharynx from 2 cm to 4	2
**HCFA001**	Submandibular gland excision enlarged	2
**HCFA004**	Partial parotidectomy without facial nerve dissection	2
**HDNE001**	Destruction of hypopharyngeal lesion with laser, by endoscopy	2

**HJND001**	Destruction of rectal lesion, anally	2
**HJFA012**	Secondary proctectomy by laparotomy with trans ileoanal anastomosis	2
**HPLB003**	Intraperitoneal administration of anticancer pharmacological agent with hyper	2
**JDQX004**	Pathological examination for oncological purposes of total cystectomy specimen	2
**JHQX008**	Pathological examination for oncological purposes of partial amputation specimen	2
**JAFA032**	Total nephroureterectomy, by direct approach	2
**JAFA016**	Binephrectomy, by laparotomy	2
**JAFA029**	Total nephrectomy extended to the renal compartment with adrenalectomy, by laparotomy	2
**JDFA001**	Total cystectomy with cutaneous ureterostomy, by laparotomy	2
**JKFA020**	Total colpohysterectomy extended to parameters, by laparoscopy and by vaginal approach	2
**LBFA005**	Total maxillectomy without reconstruction	2
**LHFH001**	Vertebral bone tumorectomy, transcutaneously with CT guidance	2
**PAFH001**	Osteoarticular tumorectomy of a limb, transcutaneously with sc guidance	2
**ZZNL061**	External irradiation session using a machine with a power of less than 5 megavolts	2
**ZZNL045**	External proton irradiation session	2
**YYYY050**	Irradiation of less than 300 cm_ in fixed fields by accelerator	2
**YYYY244**	Irradiation of less than 300 cm_ in fixed fields by accelerator	2
**YYYY304**	Irradiation of 300 cm_ and more in fixed fields by accelerator	2
**YYYY337**	Irradiation of 300 cm_ and more in fixed fields by accelerator	2
**YYYY492**	Irradiation of 300 cm_ and more in fixed fields by accelerator	2
**YYYY055**	Irradiation in cyclotherapy by accelerator in fractions of 20 centiGrays	2
**HJFA001**	Rectocolic resection with retrorectal colic lowering by laparotomy	1
**HELE001**	Installation of intra-esophageal guide for brachytherapy	1
**LAFA014**	Mastoidectomy or total atticomastoidectomy	1
**JJQX005**	Pathological examination for carcinological purposes of several ovariects	1
**JJFA003**	Unilateral oophorectomy, by laparotomy	1
**JJFA005**	Bilateral oophorectomy, by laparotomy	1
**HNFA008**	Left pancreatectomy with preservation of the spleen, by laparotomy	1
**HNFA004**	Total duodenopancreatectomy with splenectomy	1
**GENE006**	Destruction of lesion of the tracheobronchial tree by cryotherapy or thermocoagulation	1

**GFFA008**	Upper lung lobectomy with side resection and plexus release	1
**GFFA019**	Excision of remaining lung lobe, by thoracotomy	1
**GFFA007**	Pneumonectomy with resection of organ and/or neighboring structure, by thoracotomy	1
**JGND002**	Prostate cryotherapy	1
**FENL001**	Internal irradiation for polycythemia, by intravenous injection of phosphorus	1
**ZZMP001**	Preparing for total body irradiation	1
**JKFC002**	Subtotal hysterectomy, by laparoscopy	1
**JKFA024**	Subtotal hysterectomy, by laparotomy	1
**JKFA005**	Total hysterectomy with unilateral or bilateral annexectomy, via vaginal approach	1
**JLNL002**	High dose rate intracavitary vaginal brachytherapy with two-dimensional dosimetry	1
**JDFA021**	Total vesiculo-prostato-cystectomy with replacement enterocystoplasty	1
**BKQX004**	Pathological examination for oncological purposes of exenteration part of the orbit	1
**DZQX005**	Pathological examination for oncological purposes of a tumor excision specimen	1
**FAFA010**	Tonsillectomy by dissection, with adenoidectomy and bilateral aerator placement	1
**GCQX005**	Pathological examination for oncological purposes of rhinoplasty specimen	1
**GBFA008**	Bilateral anterior ethmoidectomy with nasal septoplasty, by endoscopy	1
**GDFA010**	Arytenoidectomy or arytenoidopexy, by thyrotomy	1
**GDFA014**	Supraglottic laryngectomy	1
**GDFA011**	Supracricoid laryngectomy with crico-hyoido-epiglottopexy	1
**GHFA003**	Excision of mediastinal tumor with resection of organ and/or voila structure	1
**HAFA001**	Basic partial glossectomy, by cervicotomy	1
**HAFA003**	Total mobile tongue glossectomy, via cervicofacial approach	1
**HAFA027**	Mobile tongue pelviglossectomy, via cervicofacial approach	1
**HAFA033**	Non-interrupting pelvimandibulectomy, via intraoral approach	1
**HAFA024**	Non-interrupting pelvimandibulectomy, via cervicofacial approach	1
**HAFA002**	Non-interrupting pelvi-glosso-mandibulectomy, via intraoral approach	1
**HAFA026**	Non-interrupting pelvi-glosso-mandibulectomy, via cervicofacial approach	1
**HCFA005**	Total parotidectomy with excision of the parapharyngeal extension	1

**HDNE002**	Destruction of hypopharyngeal lesion without laser, by endoscopy	1
**HDFA004**	Oropharyngectomy with non-interrupting partial mandibulectomy	1
**HDFA010**	Total circular pharyngolaryngectomy	1
**HHFA029**	Total coloproctectomy without restoration of continuity, by laparoscopic or laparotomy	1
**HLNN900**	Destruction of hepatic tumor by radiofrequency, transcutaneously	1
**JEQX005**	Pathological examination for oncological purposes of partial ureterectomy	1
**JHQX007**	Pathological examination for oncological purposes of total amputation specimen	1
**JKQX006**	Pathological examination for oncological purposes of trachelectomy specimen	1
**JMQX005**	Pathological examination for oncological purposes of specimen of vulvectomy	1
**JAFA030**	Partial nephrectomy without dissection of the vascular pedicle, by laparotomy	1
**JAFA019**	Partial nephrectomy with dissection of the vascular pedicle, by laparotomy	1
**JAFA002**	Total nephrectomy, by lombotomy	1
**JAFC001**	Total nephroureterectomy, by laparoscopic route or retroperitoneoscopy	1
**JAFC019**	Total nephrectomy extended to the renal compartment, by laparoscopy or retroperitoneoscopy	1
**JDFA011**	Partial cystectomy, by laparotomy	1
**JDFA005**	Total cystectomy, by laparotomy	1
**JDFA016**	Total cystectomy with replacement enterocystoplasty	1
**JKND004**	Destruction of lesion of the cervix by colposcopy, without laser	1
**JKFA027**	Total colpohysterectomy extended to parameters, by laparotomy	1
**JMFA004**	Total vulvectomy with bilateral inguinal and iliac lymph node dissection	1
**JFFA003**	Anterior pelvectomy with transintestinal cutaneous ureterostomy by loop	1
**KCFA007**	Total thyroidectomy, by cervicothoracotomy	1
**KCMA001**	Secondary totalization of thyroidectomy, by cervicotomy	1
**LBFA006**	Upper maxillectomy without reconstruction	1
**LBFA018**	Shaping resection of the mandible, via intraoral approach	1
**PZQX004**	Pathological examination for oncological purposes of partial amputation specimen	1
**QEQX005**	Pathological examination for oncological purposes of several mastectomy specimens	1

**QEQX007**	Pathological examination for oncological purposes of several mastectomy specimens	1
**QZFA033**	Excision of superficial lesion of the skin by excision of a skin area	1
**ZZMK019**	Resumption of preparation for external irradiation without dosimetry, with simulation	1
**ZZNL064**	External irradiation session by linear accelerator of equal or greater power	1
**ZZNL055**	External irradiation in stereotaxic conditions by dedicated machine	1
**ZZNL018**	Deep interstitial brachytherapy with pulsed dose rate, with tri dosimetry	1
**ZZNL003**	Interstitial brachytherapy with pulsed dose rate after vector placement	1
**ZZMP004**	Manufacturing of a personalized applicator for brachytherapy	1
**ZZMP003**	Manufacturing of personalized lead protection for brachytherapy	1
**YYYY307**	Irradiation of 300 cm_ and more in fixed fields by accelerator per fraction of 20 centigrays for beams ranging from 5 to 7.9 MeV.	1
**YYYY347**	Irradiation of 300 cm_ and more in fixed fields by accelerator per fraction of 1.4 grays for beams ranging from 0.5 to 4.9 MeV.	1
**YYYY387**	Irradiation of 300 cm_ and more in fixed fields by accelerator per fraction of 2 grays for beams ranging from 5 to 7.9 MeV.	1
**YYYY481**	Irradiation of 300 cm_ and more in fixed fields by accelerator per fraction of 12 centigrays for beams ranging from 17 to 24.9 MeV.	1
**YYYY316**	Irradiation of 300 cm_ and more in fixed fields by accelerator per fraction of 1.2 grays for beams ranging from 17 to 24.9 MeV.	1
**YYYY360**	Irradiation of 300 cm_ or more in fixed fields by accelerator per fraction of 12 grays for beams more than 25 MeV.	1
**YYYY451**	Irradiation of 300 cm_ or more in fixed fields by accelerator per fraction of 0.9 grays for beams more than 25 MeV.	1
**YYYY267**	Irradiation in cyclotherapy by accelerator in fractions of 12 centiGrays	1

Table S4: Summary of protocol components of the emulated target trials.

Protocol Component	Description of Emulation
**Research questions**	Is EVR treatment after LT associated with a lower occurrence of DNM?<br>Is TAC discontinuation in an EVR initiator involved in the occurrence of DNM?
**Outcomes**	DNM occurrence<br>Overall survival
**Eligibility criteria**	Discharged after LT<br>Older than 18 years<br>First liver transplantation between 01/01/2009 and 01/01/2020
**Exclusions**	No exclusion criteria
**Treatment strategies**	ETT1: EVR initiation or not<br>ETT2: EVR discontinuation or not in EVR initiator<br>ETT3: TAC discontinuation or not in EVR initiator
**Treatment assignment**	Emulated randomization
**Follow up time**	ETT1: 10 years after LT<br><br>ETT2 or ETT3: 10 years after initiation
**Grace periods**	ETT1: 3 years after LT<br><br>ETT2 or ETT3: 3 years after EVR initiation
**Estimand**	Hazard ratio
**Analysis plan**	Emulation of target trial: Analysis conducted on the cloned data set assuming random treatment assignment and with a censoring indicator when there is a protocol deviation within the grace period; selection bias accounted for by using inverse probability of artificial censoring weights.<br><br>Type of primary analysis and statistical model: HR approach
**Adjustment variables**	Based on Cox regression analysis for DNM occurrence: age at transplant, sex, smoking status, alcohol-related liver disease, diabetes before transplant, HCC as transplant indication, recurrence of HCC post-transplant, pre-transplant creatinine, HCV, income level, chronic kidney disease, local unemployment rate, pre-transplant ascites, hepatic encephalopathy, and transplant center

**Sensitivity analyses**	Site-specific analysis<br><br>Role of grace period and lag-time

Table S5: Multivariate Generalized Additive Model (GAM).

To validate the associations suggested by the IPCW-RF, a parametric generalized additive model (GAM) was fitted. This approach estimates adjusted effects for each covariate on the hazard of death or retransplantation. This table reports the estimated coefficients (Estimate), standard errors (SE), z-values and corresponding p-values. Variables with p <0.05 were considered to be statistically significant.

Variable	OR (95% CI)	z-value	p-value
**Demographic**			
**Female sex**	0.87 (0.76–0.99)	−2.09	0.037
**Socioeconomic factors**			
**Area-level unemployment**	1.03 (1.01–1.05)	2.77	0.006
**Area-level income**	1.00 (1.00–1.00)	−0.10	0.919
**Pre-transplant comorbidities**			
**Tobacco use**	1.10 (0.99–1.24)	1.72	0.085
**Alcohol-related liver disease**	1.10 (0.96–1.26)	1.34	0.180
**Cardiovascular disease**	1.11 (0.98–1.25)	1.65	0.098
**Diabetes mellitus**	0.93 (0.83–1.03)	−1.36	0.175
**Arterial hypertension**	1.00 (0.88–1.14)	0.02	0.983
**Stroke**	1.33 (1.01–1.76)	2.00	0.046
**Sleep apnea**	1.00 (0.81–1.24)	0.03	0.977
**Psychiatric disorders**	0.93 (0.80–1.09)	−0.90	0.368
**HIV infection**	0.65 (0.44–0.96)	−2.15	0.032
**Pre-LT renal failure**	0.57 (0.37–0.89)	−2.45	0.014
**Liver disease severity before transplantation**			
**Ascites**	0.96 (0.85–1.08)	−0.68	0.498
**Hepatic encephalopathy**	0.90 (0.80–1.02)	−1.59	0.113
**Sepsis before LT**	0.97 (0.86–1.10)	−0.45	0.656
**TIPS procedure**	1.18 (0.96–1.46)	1.57	0.116
**Acute hemodialysis before LT**	1.04 (0.93–1.16)	0.67	0.504
**Underlying liver disease**			
**HCC as primary indication**	1.47 (1.30–1.66)	6.18	<0.001
**Hepatitis C infection**	0.88 (0.78–1.00)	−1.95	0.052
**Hepatitis B infection**	0.85 (0.72–1.01)	−1.86	0.063
**Autoimmune liver disease**	1.10 (0.82–1.47)	0.62	0.534
**Secondary biliary cirrhosis**	0.77 (0.54–1.09)	−1.47	0.142
**Cancer history**			

**Extrahepatic cancer before LT**	1.16 (0.72–1.86)	0.61	0.539
**Cancer before transplantation**	1.30 (0.72–2.34)	0.88	0.377
**Post-transplant events**			
**HCC recurrence**	4.25 (3.38–5.33)	12.47	<0.001
**Renal dysfunction after LT**	1.34 (1.20–1.49)	5.28	<0.001

Table S6: Covariate balance before and after inverse probability of censoring weighting.

Covariate	No-EVR strategy (Unweighted)	EVR strategy (Unweighted)	No-EVR strategy (IPCW-weighted)	EVR strategy (IPCW-weighted)	Absolute SMD
**Baseline covariates**					
**Age at liver transplantation (years)**	53.01	53.63	53.25	53.63	0.033
**Extrahepatic cancer count before LT**	0.052	0.056	0.052	0.056	0.013
**Area-level income**	17587	17572	17586	17572	0.002
**Area-level unemployment**	8.094	8.112	8.104	8.112	0.003
**Sex**	0.233	0.223	0.229	0.223	0.005
**Tobacco use**	0.318	0.318	0.318	0.318	<0.001
**Alcohol-related liver disease**	0.663	0.680	0.669	0.680	0.011
**Cardiovascular disease**	0.237	0.246	0.239	0.246	0.007
**Diabetes mellitus**	0.331	0.340	0.335	0.340	0.006
**Arterial hypertension**	0.699	0.707	0.701	0.707	0.006
**Stroke**	0.025	0.028	0.026	0.028	0.002
**Sleep apnea**	0.055	0.056	0.055	0.056	0.001
**Psychiatric disorders**	0.102	0.099	0.099	0.099	<0.001
**Inflammatory bowel disease**	0.039	0.036	0.038	0.036	0.002
**HIV infection**	0.027	0.024	0.026	0.024	0.001
**Pre-LT renal failure**	0.016	0.016	0.016	0.016	<0.001
**Cancer before transplantation**	0.045	0.048	0.045	0.048	0.003

**Ascites**	0.572	0.572	0.573	0.572	<0.001
**Hepatic encephalopathy**	0.277	0.274	0.276	0.274	0.002
**Sepsis before LT**	0.232	0.228	0.229	0.228	0.001
**TIPS procedure**	0.054	0.058	0.055	0.058	0.002
**HCC as primary indication**	0.418	0.443	0.425	0.443	0.018
**Hepatitis C infection**	0.286	0.283	0.287	0.283	0.004
**Hepatitis B infection**	0.118	0.115	0.116	0.115	0.001
**Hepatitis D coinfection**	0.028	0.027	0.028	0.027	0.001
**Autoimmune liver disease**	0.044	0.041	0.043	0.041	0.001
**Secondary biliary cirrhosis**	0.037	0.034	0.036	0.034	0.001
**Time-varying covariates**					
**HCC recurrence**	0.023	0.031	0.024	0.031	0.007
**Renal dysfunction**	0.211	0.232	0.215	0.232	0.017

Table S7: Effects of treatments in trials ETT-1 to ETT-3: risk difference (RD), hazard ratio (HR), number needed to treat/harm (NNT/NNH), and E-value

Trial	RD median (IQR)	HR median (95% CI)	NNT / NNH	E-value (point ; lower)
**ETT-1**	−0.0646 (−0.0963; −0.0428)	0.84 (0.79–0.88)	NNT 16 (10–23)	1.67
**ETT-2**	0.0505 (0.0341; 0.0765)	1.34 (1.21–1.45)	NNH 20 (13–29)	1.84
**ETT-3**	0.1258 (0.1159; 0.1388)	1.03 (0.83–1.21)	NNH 8 (7–9)	1.16

*RD = Risk Difference; HR = Hazard Ratio; NNT = Number Needed to Treat; NNH = Number Needed to Harm; IQR = Interquartile Range; CI = Confidence Interval.

Table S8: Sensitivity analyses of ETT-2 using alternative dispensing-gap definitions (30, 60, and 180 days).

Gap definition	Outcome	HR, median (Q1–Q3)	RD, median (Q1–Q3)	NNT/NNH	E-value
**30 days**	DNM occurrence	1.40 (1.37–1.43)	0.06 (0.05–0.06)	17.28 (NNH)	2.15
**30 days**	Overall survival	1.41 (1.36–1.46)	−0.02 (−0.06 to 0.01)	43.86 (NNT)	2.17
**60 days**	DNM occurrence	1.43 (1.29–1.55)	0.08 (0.05–0.10)	12.90 (NNH)	2.23
**60 days**	Overall survival	1.88 (1.81–2.13)	0.19 (0.09–0.33)	5.19 (NNH)	3.18
**180 days**	DNM occurrence	1.60 (1.58–1.63)	0.06 (0.05–0.09)	16.10 (NNH)	2.58
**180 days**	Overall survival	2.03 (2.00–2.25)	0.03 (−0.10 to 0.07)	34.21 (NNH)	3.49

Abbreviations: HR, hazard ratio; RD, risk difference; NNT, number needed to treat; NNH, number needed to harm. Gap definitions refer to the maximum allowed interval between dispensings used to define treatment discontinuation in ETT-2.
