# Supplementary material for: Everolimus and de novo malignancies after liver transplantation: A nationwide emulated trial using real-world data
Source: JHEP Rep. 2026 Aug 7;8(9):101934. doi: 10.1016/j.jhepr.2026.101934 (PMC13476556; doi:10.1016/j.jhepr.2026.101934)
Supplement: Supplementary file 3 [file mmc4.pdf]

Everolimus and *de novo* malignancies after liver transplantation: A nationwide emulated trial using real-world data

Authors

Ilias Kounis, Tri-Long Nguyen, Christophe Desterke, …, Audrey Coilly, Paul Landais, Cyrille Féray

Correspondence

ilias.kounis@aphp.fr (I. Kounis).

Graphical abstract

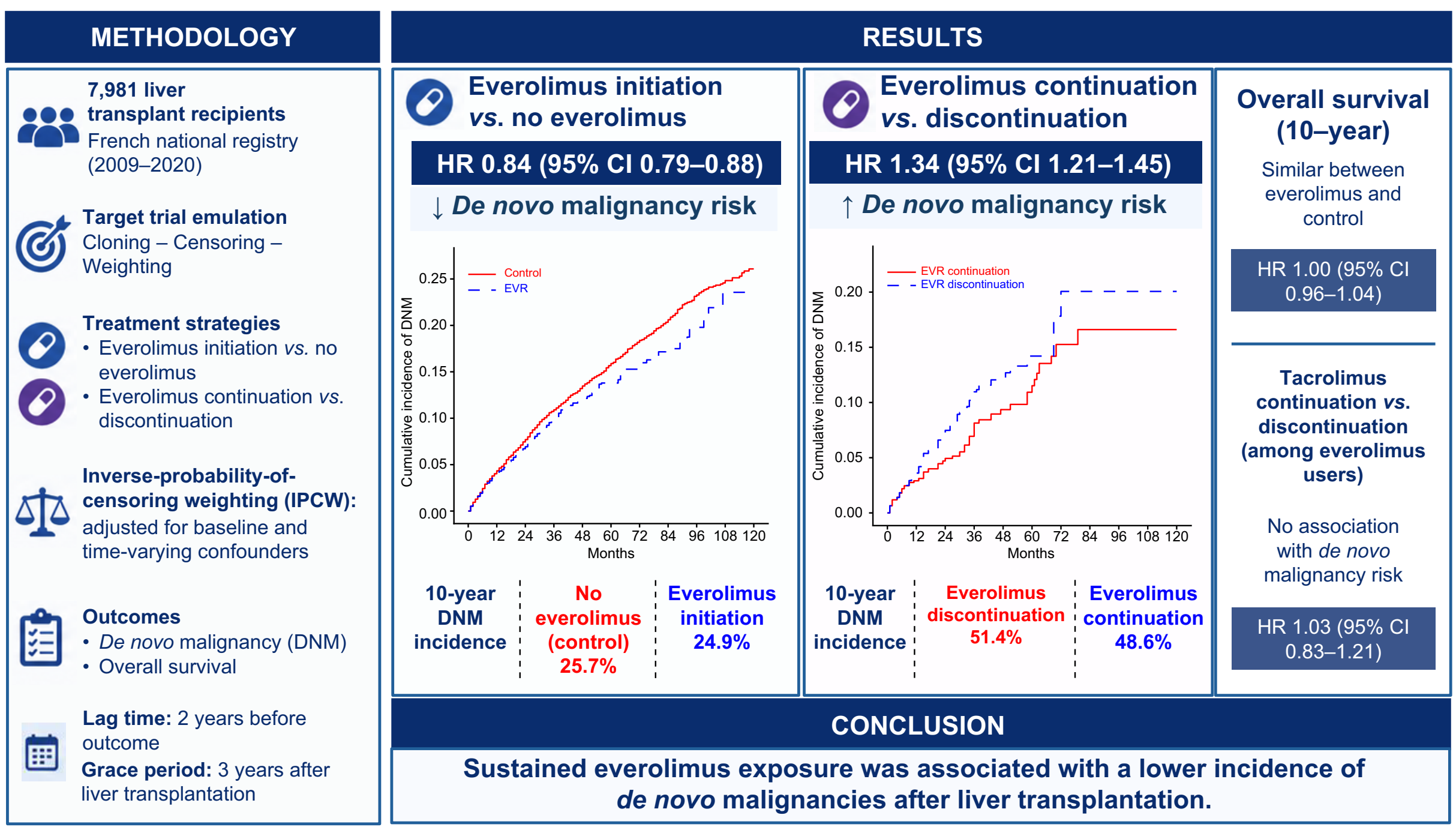

Highlights:

- *De novo* malignancies remain a major long-term complication after liver transplantation.
- A nationwide target trial emulation using cloning, censoring and weighting evaluated long-term everolimus exposure.
- Everolimus initiation reduced de novo malignancy risk, whereas discontinuation increased the risk.
- These findings support sustained everolimus exposure as a potential strategy to reduce post-transplant cancer burden.

Impact and implications:

This study addressed the lack of randomized evidence regarding the role of everolimus (EVR) in preventing *de novo* malignancies after liver transplantation by leveraging a causal inference framework applied to nationwide data. These findings are important for transplant physicians, researchers, and policymakers, as they suggest that sustained EVR exposure is associated with a reduced risk of post-transplant malignancies without compromising overall survival. In practice, this supports the consideration of EVR as part of immunosuppressive strategies and highlights the potential risks associated with its discontinuation. These results may inform clinical decision-making and future research on optimizing long-term immunosuppression to balance graft protection and cancer risk.

https://doi.org/10.1016/j.jhepr.2026.101934
© 2026 The Author(s). Published by Elsevier B.V. on behalf of European Association for the Study of the Liver (EASL). This is an open access article under the CC BY license (http://creativecommons.org/licenses/by/4.0/). JHEP Reports, 2026, 8, 1–13

JHEP|Reports

Everolimus and *de novo* malignancies after liver transplantation: A nationwide emulated trial using real-world data

Ilias Kounis[1,2,3,4,*], Tri-Long Nguyen[5], Christophe Desterke[1], Nathalie Goutte[2], Didier Samuel[1,2,3,4], Daniel Azoulay[1,2,3,4], Eric Vibert[1,2,3,4], Faouzi Saliba[1,2,3,4], Audrey Coilly[1,2,3,4], Paul Landais[6], Cyrille Féray[1,2,3,4]

JHEP Reports **2026**. vol. 8 | 1–13

Check for updates

Background & Aims: Liver transplantation (LT) is associated with an increased risk of *de novo* malignancies (DNMs). Everolimus is an mTOR inhibitor with immunosuppressive and potentially antineoplastic properties. We aimed to evaluate the association between everolimus use and the incidence of DNMs following LT.

Methods: Emulated target trials were conducted using French nationwide administrative health records. The cloning–censoring–weighting approach was applied to estimate the effects of everolimus initiation, everolimus discontinuation, and concomitant tacrolimus use among everolimus users. The primary outcome was the cumulative incidence of DNMs. Secondary outcomes included overall survival and site-specific cancer incidence.

Results: A total of 7,981 patients underwent LT for cirrhosis or hepatocellular carcinoma (44%) between 2009 and 2020. The 10-year cumulative incidence of DNMs was 25.7%. DNMs were less frequent among patients who initiated everolimus within the first 3 years after LT (hazard ratio [HR] 0.84; 95% CI 0.79–0.88). Everolimus discontinuation was associated with an increased risk of DNMs (HR 1.34; 95% CI 1.21–1.45), whereas concomitant tacrolimus use was not significantly associated with DNM risk (HR 1.03; 95% CI 0.83–1.21). Site-specific emulated target trials demonstrated lower incidences of bladder, hematological, lung, prostate, skin, and lymphoid malignancies with everolimus use. The main findings remained robust in sensitivity analyses using alternative definitions of lag times and grace periods.

Conclusion: Sustained everolimus exposure was associated with a lower incidence of *de novo* malignancies after LT. These findings support everolimus-based immunosuppressive strategies incorporating calcineurin inhibitor minimization to potentially reduce long-term oncologic risk.

© 2026 The Author(s). Published by Elsevier B.V. on behalf of European Association for the Study of the Liver (EASL). This is an open access article under the CC BY license (http://creativecommons.org/licenses/by/4.0/).

Introduction

Liver transplantation (LT) is associated with an elevated risk of developing *de novo* malignancies (DNM).[1–3] This risk is largely attributable to the long-term immunosuppressive therapy that is essential to prevent allograft rejection but may impair antitumor immune surveillance.[4] Immunosuppressive regimens vary in terms of their effects on rejection and cancer risk.[5]

Calcineurin inhibitors (CNIs) such as cyclosporine and tacrolimus (TAC) are standard first-line immunosuppressants[6] but are linked to nephrotoxicity[7] and cancer.[8] These limitations have encouraged the adoption of mammalian target of rapamycin (mTOR) inhibitors.[9] Everolimus (EVR) has so far been evaluated with and without CNIs.[10,11] EVR improves renal function while maintaining immunosuppression when introduced early after LT.[10–13] At higher doses, EVR exhibits antitumor activity,[14–16] thus supporting its use in LT recipients with prior hepatocellular carcinoma or an elevated cancer risk.[17] However, due to concerns regarding wound-healing complications,[18] EVR is generally a second-line option.

European guidelines recommend minimizing CNI exposure in patients with a high risk of hepatic or extrahepatic cancer recurrence, and suggest mTOR inhibitor-based regimens for recurrent or *de novo* non-melanoma skin cancer.[17] However, the effects of mTOR inhibitors on post-transplant DNM or a recurrence of HCC in the native liver are unclear. Meta-analyses of retrospective studies have suggested that mTOR inhibitor-based immunosuppression may decrease HCC recurrence and improve survival, although study heterogeneity has limited any definitive interpretation.[19–24] A recent Korean population-based study also reported the potential benefits of EVR in reducing HCC recurrence.[25] The only randomized controlled trial to have evaluated mTOR inhibitors relative to HCC after LT did not demonstrate overall efficacy, although subgroup analyses indicated potential benefits in patients with more active tumors.[26] Other evidence has shown that

* Corresponding author. Address: AP-HP Hôpital Paul-Brousse, Centre Hépato-Biliaire, 12 Avenue Paul Vaillant Couturier, 94800, Villejuif, France.
E-mail address: ilias.kounis@aphp.fr (I. Kounis).
https://doi.org/10.1016/j.jhepr.2026.101934

cumulative TAC exposure increases the incidence of post-transplant cancer.[27] In kidney transplantation, mTOR inhibitors reduce non-melanoma skin cancer, although their effects on other malignancies remain uncertain.[28,29]

To date, no randomized trial has assessed the efficacy of EVR in preventing DNM after LT, largely because such trials require long-term follow-up and large samples.[30] However, using nationwide French data, we were able to evaluate the effect of EVR on DNM after LT by applying a cloning-censoring-weighting (CCW) framework to mimic randomized trials.[31–34] Our objective was to estimate the impact of EVR initiation on DNM incidence and post-LT survival, including the consequences of subsequent EVR discontinuation.[35]

Materials and methods

Ethics and data protection

This project was approved by the French National Commission for Information and Liberty (CNIL N°919274). No informed consent was required as the data were de-identified and re-used for research, in accordance with French regulations applicable to SNDS data.

Data sources and measurements

The study population included patients discharged after their first LT, which had been required for decompensated cirrhosis (*e.g*. portal hypertension-related bleeding, ascites, and hepatic encephalopathy), primary liver tumors, or both, between January 1, 2009, and January 1, 2020. No exclusion criteria were applied.

This study used data from the French National Health Data System (*Système National de Données de Santé* – SNDS), covering nearly the entire French population of 67.7 million residents.[36] The SNDS combines data from the *Datamart de Consommation Inter-Régimes* (DCIR), *Programme de Médicalisation des Systèmes d'Information* (PMSI), and CEPIDC.[37] The DCIR captures outpatient healthcare procedures performed in ambulatory settings, while the PMSI records diagnoses and procedures associated with hospital care. The SNDS includes demographic data, hospital discharge reports, outpatient care information, and long-term sickness records.[38] Diagnostic codes used ICD-10, procedures were identified using the Common Classification of Medical Procedures (CCMP) classification, and medications were recorded using CIP codes linked to the WHO ATC classification. Medication units were calculated from dispensing records, with treatment considered to be continuous if any interruptions lasted less than 90 days. If medication was being received within the final study year, treatment was considered to be ongoing until the end of the study. During hospitalization, the treatment days matched the length of stay. All patients received immunosuppressive treatment during the study period.

LT was identified using the CCMP procedure codes HLEA001 (total liver transplantation) or HLEA002 (partial liver transplantation).[39] Hospital discharge after LT was defined as day 0. Chemotherapy and radiotherapy sessions were identified through ICD-10 codes, CCMP procedures, and ATC molecule codes. Treatment was confirmed if chemotherapy was ongoing at least 90 days after discharge.

The area of residence was defined by the French *'département'* zip code at LT. For social deprivation assessments, we used municipality-level indicators that included percentages of unemployment, high school graduation, and those in work. The socioeconomic index was calculated as the mean of these indicators within their *département*.[40] Patients born overseas were also identified.

Risk factors were collected, including tobacco use, alcohol use, metabolic syndrome, HIV, HBV, HCV prior to LT, and cancer history. The diagnostic algorithms used to identify liver transplant recipients, the ICD-10 codes for DNM classification and CCMP codes are shown in Tables S1–3.

Outcome definition: DNMs

The primary outcome was the occurrence of any type of DNM. The secondary outcome was overall survival.

DNM occurrence was defined by the association of (i) an ICD-10 cancer diagnosis code, excluding *in situ* tumors, and (ii) a cancer-related CCMP procedure (surgery, radiotherapy, radiofrequency ablation, cryotherapy, insertion of vascular access for chemotherapy and the administration of chemotherapy). Each ICD-10 code was first classified according to tumor site (lung, head and neck, kidney and ureter, colorectal, breast, prostate, pancreas, and uterus) and then aggregated into broader cancer categories: gynecological, prostate, skin (excluding melanoma), lymphoma, colorectal, bladder, lung, and head and neck cancers. The time to DNM was defined as the interval between day 0 and the first occurrence of any DNM.

Exposures

Three treatment strategies were examined. The first corresponded to the initiation of EVR within a grace period after discharge following the first LT. The second and third corresponded to the discontinuation of EVR or tacrolimus, respectively, within a grace period after EVR initiation.

Statistical analysis

For descriptive purposes, continuous variables are presented as medians and interquartile ranges, and categorical variables as counts.

Cumulative incidence and overall survival

First, we estimated the cumulative incidence of all DNM types, site-defined DNMs, invasive cancer, and all-cause mortality. Time-to-event was defined as the interval between hospital discharge after LT and the occurrence of DNM or death. Survival following DNM was also evaluated. Patients were censored administratively on January 1, 2021, with administrative censoring assumed to be non-informative. Descriptive cumulative incidence curves were generated using competing-risk methods, with death as a competing event. To estimate the effects of EVR under a target trial emulation framework, we applied a CCW approach: each patient was duplicated and assigned to both treatment strategies at baseline, censored upon deviation from the assigned strategy, and weighted using inverse-probability-of-censoring weights. Event-time analyses were performed using weighted cause-specific Cox models.

Emulated trial 1: EVR initiation vs. no EVR

In emulated target trial 1 (ETT-1), we evaluated the effect of initiating EVR on DNM occurrence and survival. To emulate the target trial, we implemented a CCW approach (Table S4).

Cloning

At baseline (date of hospital discharge after liver transplantation), each participant was cloned into two copies: one assigned to the EVR initiation strategy and the other to the no-EVR strategy. Cloning ensured that both trial arms were identical with respect to baseline characteristics. Time zero was defined as the date of discharge after LT, when patients became eligible to receive EVR.

Grace period

Initiating EVR within 3 years (*grace period*[41]) was the "treatment" strategy; not initiating EVR within this period constituted the "control" strategy. During this period, participants assigned to the EVR strategy were allowed to initiate EVR at any time within 3 years of hospital discharge following LT. In contrast, participants assigned to the control strategy were expected not to initiate EVR during this time. The control arm received tacrolimus with or without mycophenolate mofetil (MMF). A 3-year grace period was selected because 88.1% of EVR-treated patients initiated therapy within this interval.

Censoring

Artificial censoring was applied when the observed treatment deviated from the assigned strategy. Artificial censoring may induce selection bias because censoring becomes informative across treatment arms. Clones were followed from baseline and were censored artificially when their observed treatment deviated from the assigned strategy, as described by Maringe *et al.*[41] and Gaber *et al.*[34] Specifically, clones assigned to the control strategy were censored at the time of EVR initiation if EVR was started within the 3-year grace period. Conversely, clones assigned to the EVR strategy were censored if they had not initiated EVR by the end of this 3-year grace period. This artificial censoring was applied according to the procedure described by Maringe *et al.*[41] and Gaber *et al.*[34]

DNMs occurring during the grace period were counted as events under both strategies because individuals were cloned at baseline and followed under each treatment assignment. This prevented immortal time bias because event occurrence was not conditional on future treatment initiation.

Lag time

To estimate the effects of EVR, we defined a 2-year lag between EVR initiation and outcome to reduce any potential reverse causality. EVR could be initiated before hospitalization when a DNM was first coded, thus creating a potential bias. This meant that patients assigned to the EVR arm who received EVR within the grace period, but in whom we observed DNM within 2 years of treatment initiation, were censored at the time of treatment initiation. This was necessary because the data collected on those patients could only inform us about their survival between time zero and EVR initiation. This induced a selection bias due to conditionality on the future, with two important considerations: i) this bias was symmetrical across the two cloned arms (EVR *vs.* control) since the patients involved were censored at precisely the same time across the two arms; and ii) this selection bias was theoretically corrected by the weighting within both arms (under the assumption of no omitted variables).

Weighting

To address the selection bias introduced by artificial censoring, we computed a time-varying inverse probability of censoring weights. Thirty covariates were included to estimate the time-varying probability of remaining uncensored. Covariates were retained if they were clinically relevant to EVR use or to post-transplant outcomes (Figs. S2 and S3), or if they demonstrated significant associations with EVR initiation in generalized additive models (Table S5). All time-varying covariates were updated during follow-up before estimating the probability of remaining uncensored.

The selected covariates included both baseline characteristics and time-varying confounders. Baseline covariates included age at liver transplantation, sex, area-level income, area-level unemployment, tobacco use, alcohol-related liver disease, cardiovascular disease, diabetes mellitus, arterial hypertension, stroke, sleep apnea, psychiatric disorders, inflammatory bowel disease, HIV infection, hepatitis C infection, hepatitis B infection, hepatitis D coinfection, autoimmune liver disease, secondary biliary cirrhosis, ascites, hepatic encephalopathy, sepsis before transplantation, TIPS (transjugular intrahepatic portosystemic shunt) placement, pre-transplant hemodialysis (chronic and acute), extrahepatic cancer count before transplantation, any cancer before transplantation, and hepatocellular carcinoma as the primary indication for transplantation.

Time-varying confounders included tumor recurrence and renal dysfunction, which were considered key confounders because they are major clinical indications for EVR initiation after liver transplantation. EVR is frequently introduced in the context of post-transplant renal dysfunction or oncological indications, such as prior HCC or HCC recurrence. Some potential confounders, such as treatment-related adverse events, could not be accounted for because they are not captured in the SNDS database.

Covariate balance

Covariate balance before and after weighting was assessed, with the inverse probability of censoring weights truncated at the 99th percentile. Under the CCW framework, the weighted sample approximated a pseudo-population in which measured confounding and selection biases were minimized. We assumed that these selected covariates ensured exchangeability between censored and uncensored observations. To evaluate the reduction of measured confounding, we examined standardized mean differences (Table S6), and a Love plot is shown in Fig. S3. For ETT 1 to 3, we computed E-values for point estimates and confidence interval limits closest to the null in order to assess the robustness of treatment effects to unmeasured confounding.[42]

Numbers needed to treat (NNT) and numbers needed to harm (NNH) were derived from the absolute difference in cumulative incidence functions between treatment strategies at 10 years.[43] The estimand was the hazard ratio (HR) among

patients discharged after their first LT. 95% CIs for HRs were obtained using non-parametric bootstrapping with 500 replicates, repeating the entire CCW procedure at each replicate to capture overall estimation uncertainty.

Emulated trial 2: EVR discontinuation vs. EVR continuation

To assess the role of EVR, we evaluated the effect of EVR discontinuation on the DNM risk among patients who initiated EVR. EVR could be discontinued during the grace period after initiation, and a lag time was applied to account for the interval between EVR withdrawal and outcome occurrence. Day 0 was defined as the EVR initiation date. In ETT-2, the control arm corresponded to continuing EVR beyond the grace period with standard background immunosuppression, whereas the intervention arm included patients who discontinued EVR within 3 years of initiation.

Emulated trial 3: Discontinuation of TAC after EVR initiation

To determine whether the effects observed in ETT-1 and ETT-2 were confounded by the use of TAC, ETT-3 evaluated the impact of TAC discontinuation among patients who initiated EVR. Day 0 was defined as the EVR initiation date. The control arm corresponded to continuing TAC after EVR initiation, while the intervention arm comprised patients who discontinued TAC within the 3-year grace period. Both strategies could include MMF according to routine practice.

Sensitivity analyses for DNM occurrence and overall survival

The ETTs assessed site-specific associations between EVR initiation and individual cancer incidence. Sensitivity analyses examined alternative grace periods (1–4 years) and evaluated the impact of varying grace periods and lag times on ETT-1 estimates for DNM-free and overall survival. Additional analyses for ETT-1 restricted follow-up to the pre-2015 period to account for temporal changes in liver transplantation practice, including the introduction of direct-acting antivirals for hepatitis C infection. Analyses restricted to patients transplanted for HCC were also performed for ETT-1, given that approximately 44% of the cohort had HCC and 55% of everolimus-treated patients had a history of HCC. Alternative gap definitions (30, 60, and 180 days) were also evaluated for treatment discontinuation in ETT-2 to account for short interruptions, dose adjustments, or temporary holds of immunosuppressive therapy that may have been incompletely captured by dispensing data.

All statistical analyses used R statistical software (version 2023.12.0) with the following packages: ggplot2, ggalluvial, GGally, survival, survminer, parallel, boot, splines, dplyr, tableone, tidyr, questionr, forestmodel, WeightIt, ggplot, and cobalt.

Results

Demographic and clinical profiles of liver transplant recipients

The study population included 7,981 patients who underwent LT for decompensated cirrhosis (e.g., portal hypertensive bleeding, ascites, or hepatic encephalopathy), primary liver tumors, or both between January 1, 2009, and January 1, 2020. No exclusion criteria were applied. The cohort was 77.5% male, with a median age of 57 years (IQR 50–62). The leading indications for LT were alcohol-related liver disease (68.0%), HCV (28.3%), and HBV (11.5%). HCC was present in 44.0% of the recipients (Table 1).

Arterial hypertension was present in 71.0% of patients, diabetes mellitus in 34.0% of patients, and 32.0% had a history of tobacco use. The initial post-LT immunosuppressive regimen included TAC in 90.5% and MMF in 91.5% of cases.

Incidence of DNM and survival

The cumulative incidence of DNM was 15.1% at 5 years and 25.7% at 10 years post-transplantation (Fig. 2). The most frequent malignancies at 10 years were head and neck cancer (5.0%), skin cancer (5.0%), myeloid neoplasms (4.7%) and bladder cancer (4.5%). Other sites included prostate (2.0%), lymphoma (1.9%), colorectal (1.7%) and gynecological (0.7%) cancers.

Ten-year survival following DNM onset depended on the cancer type (Fig. 2). Head and neck (24.4%), lung (27.0%), and bladder (30.1%) cancers had the lowest survival rates. Skin (64.8%), prostate (70.4%), and gynecological (72.4%) cancers had the highest survival rates.

Emulated trial 1: EVR initiation vs*. no EVR*

During follow-up, 2,256 patients (28.3%) received EVR, 1,988 of whom (24.9% of the total cohort) initiated EVR at least 2 years before DNM occurrence or censoring (Fig. 1). Among patients treated with EVR within this grace period, concomitant immunosuppression included tacrolimus (n = 966; 48.9%), MMF (n = 486; 24.5%), and cyclosporine (n = 98; 4.9%).

After applying the clone–censor–weight approach, EVR initiation was associated with a lower 10-year risk of DNM (HR 0.84; 95% CI, 0.79–0.88). Based on the 10-year risk difference in the CCW-weighted pseudo-population (median RD –0.065), the NNT to prevent one DNM case was 16 (IQR, 10–23). The E-value for the point estimate was 1.67, indicating that an unmeasured confounder would need to be associated with both EVR initiation and DNM risk by a risk ratio of at least this magnitude (above and beyond measured covariates) to fully account for the observed association (Table S7).

Ten-year overall survival rates were similar in both arms. The HR for mortality was 1.00 (95% CI 0.96–1.04) (Fig. 3).

Emulated trial 2: EVR discontinuation *vs*. continuation

ETT-2 included 1,988 patients who initiated EVR. Time zero was defined as the date of EVR initiation. The discontinuation of EVR within a 3-year grace period was compared with continued EVR use beyond this period, applying a 2-year lag. Overall, 967 patients (48.6%) discontinued EVR.

EVR discontinuation was associated with a significantly higher risk of DNM, with a risk difference of 0.0505 (95% CI 0.0341–0.0765) and an HR of 1.34 (95% CI 1.21–1.45) (Fig. 4), corresponding to a NNH of 20 (95% CI 13–29). The E-value was 1.84 (lower bound 1.56), suggesting moderate robustness to unmeasured confounding (Table S7).

Table 1. Baseline characteristics of the study population prior to emulation of the target trial.

Variable	Total (N = 7,981)	Control arm (n = 5,993)	EVR arm (n = 1,988)
Demographics			
Male sex	6,185 (77.5%)	4,872 (81.3%)	1,683 (84.7%)
Age at LT (years)	57 [50, 62]	56 [49, 62]	58 [53, 63]
Patients transplanted in Paris and suburbs	2,737 (34.3)	2,236 (36.0)	501 (28.4)
Years of unemployment	8.4 [6.9, 9.8]	8.4 [6.9, 9.8]	8.4 [7.1, 10.0]
Annual income (euros)	17,887 [16,708, 20,226]	17,887 [16,708, 20,315]	17,922 [16,804, 19,683]
Cirrhosis-related complications			
Ascites	4,564 (57.2%)	3,675 (57.1%)	889 (57.4%)
Hepatic encephalopathy	2,187 (27.4%)	1,789 (27.8%)	398 (25.7%)
Sepsis	1,818 (22.8%)	1,491 (23.2%)	327 (21.1%)
TIPS	461 (5.8%)	349 (5.4%)	112 (7.2%)
Variceal bleeding	2,872 (36.0%)	2,280 (35.4%)	592 (38.2%)
Liver disease etiology			
Alcohol-related cirrhosis	5,431 (68.0%)	4,259 (66.2%)	1,172 (75.6%)
HCC	3,542 (44.0%)	2,681 (41.7%)	861 (55.5%)
Primary biliary cholangitis	482 (6.0%)	429 (6.7%)	53 (3.4%)
Autoimmune	330 (4.1%)	284 (4.4%)	46 (3.0%)
Biliary cirrhosis	274 (3.4%)	238 (3.7%)	36 (2.3%)
HCV	2,260 (28.3%)	1,835 (28.5%)	425 (27.4%)
HBV	921 (11.5%)	754 (11.7%)	167 (10.8%)
HDV	217 (2.7%)	184 (2.9%)	33 (2.1%)
Risk factors and comorbidities			
Alcohol use	5,431 (68.0%)	4,259 (66.2%)	1,172 (75.6%)
Tobacco consumption	2,541 (32.0%)	2,040 (31.7%)	501 (32.3%)
Diabetes	2,719 (34.0%)	2,126 (33.1%)	593 (38.3%)
Arterial hypertension	5,643 (71.0%)	4,488 (69.8%)	1,155 (74.5%)
Stroke	222 (2.8%)	159 (2.5%)	63 (4.1%)
Cardiovascular events	1,969 (25.0%)	1,524 (23.7%)	445 (28.7%)
Sleep apnea	447 (5.6%)	354 (5.5%)	93 (6.0%)
Renal failure	1,199 (15.0%)	884 (14.2%)	315 (17.9%)
Psychiatric disorders	791 (9.9%)	651 (10.1%)	140 (9.1%)
IBD	287 (3.6%)	251 (3.9%)	36 (2.3%)
HIV	195 (2.4%)	175 (2.7%)	20 (1.3%)
Cancer before LT	385 (4.8%)	283 (4.4%)	102 (6.6%)

EVR, everolimus; HCC, hepatocellular carcinoma; IBD, inflammatory bowel disease; LT, liver transplantation; TIPS, transjugular intrahepatic portosystemic shunt.
Among the 7,981 eligible liver transplant recipients, 1,988 received EVR at least 2 years before DNM occurrence or the end of follow-up.
Quantitative variables are represented as medians [Q1, Q3], and categorical variables are expressed as numbers (%).

Emulated trial 3: Discontinuation of TAC after EVR initiation

Among the 1,988 patients who initiated EVR, 1,626 (81.8%) discontinued TAC within the 3-year grace period and a 2-year lag-time. The 10-year HR for DNM occurrence was similar across the different arms (HR 1.03; 95% CI 0.83–1.21) (Fig. 5). The corresponding risk difference was 0.1258 (95% CI 0.1159–0.1388) yielding an NNH of 8 (95% CI 7–9). The E-value for the point estimate was 1.16 (lower bound 1.00), indicating that a weak unmeasured confounder would be sufficient to move the confidence interval to include the null.

Site-specific analysis ETT

Site-specific analyses were performed using the same grace periods and lag times applied in ETT-1. EVR initiation was associated with a reduced incidence of bladder (HR 0.58; 95% CI 0.52–0.61), hematological (HR 0.96; 95% CI 0.95–0.98), lung (HR 0.90; 95% CI 0.72–0.96), prostate (HR 0.64; 95% CI 0.59–0.73), skin (HR 0.92; 95% CI 0.88–0.94), and lymphoma (HR 0.77; 95% CI 0.73–0.81) malignancies. No effect was observed among head and neck (HR 1.04; 95% CI 0.99–1.11), colorectal (HR 1.05; 95% CI 1.04–1.11), or gynecological (HR 1.09; 95% CI 0.86–1.33) cancers (Fig. S4).

Sensitivity analysis

Varying the grace periods and lag times changed the number of treated patients, potentially influencing any previously observed associations. For each ETT, using a 10-year follow-up period, we tested six combinations: a grace period of 1–4 years and lag times of 1–3 years, excluding combinations in which the lag time equaled the grace period. In ETT-1, all six combinations showed a protective effect of EVR on DNM risk and no effect on overall survival. In ETT-2, an increased DNM risk was observed for all but one combination (grace period = 4 years, lag time = 3 years), where the number of treated patients was small because of the prolonged lag time (Fig. 6). In ETT-3, all combinations yielded similar results for 10-year DNM incidence and overall survival. We also evaluated lag time = 0. In ETT-1, the association was attenuated (Fig. S4), suggesting some degree of reverse causality when no lag was applied (EVR was prescribed because a DNM had been detected in an outpatient setting, and the DNM was coded later in the electronic health records). In ETT-2, removing the lag time had no effect, likely because DNM detection did not prompt EVR discontinuation.

The weighting phase of the CCW approach is critical. The weighting of our covariates was verified using a paired t-test, as shown in `Fig. S4.

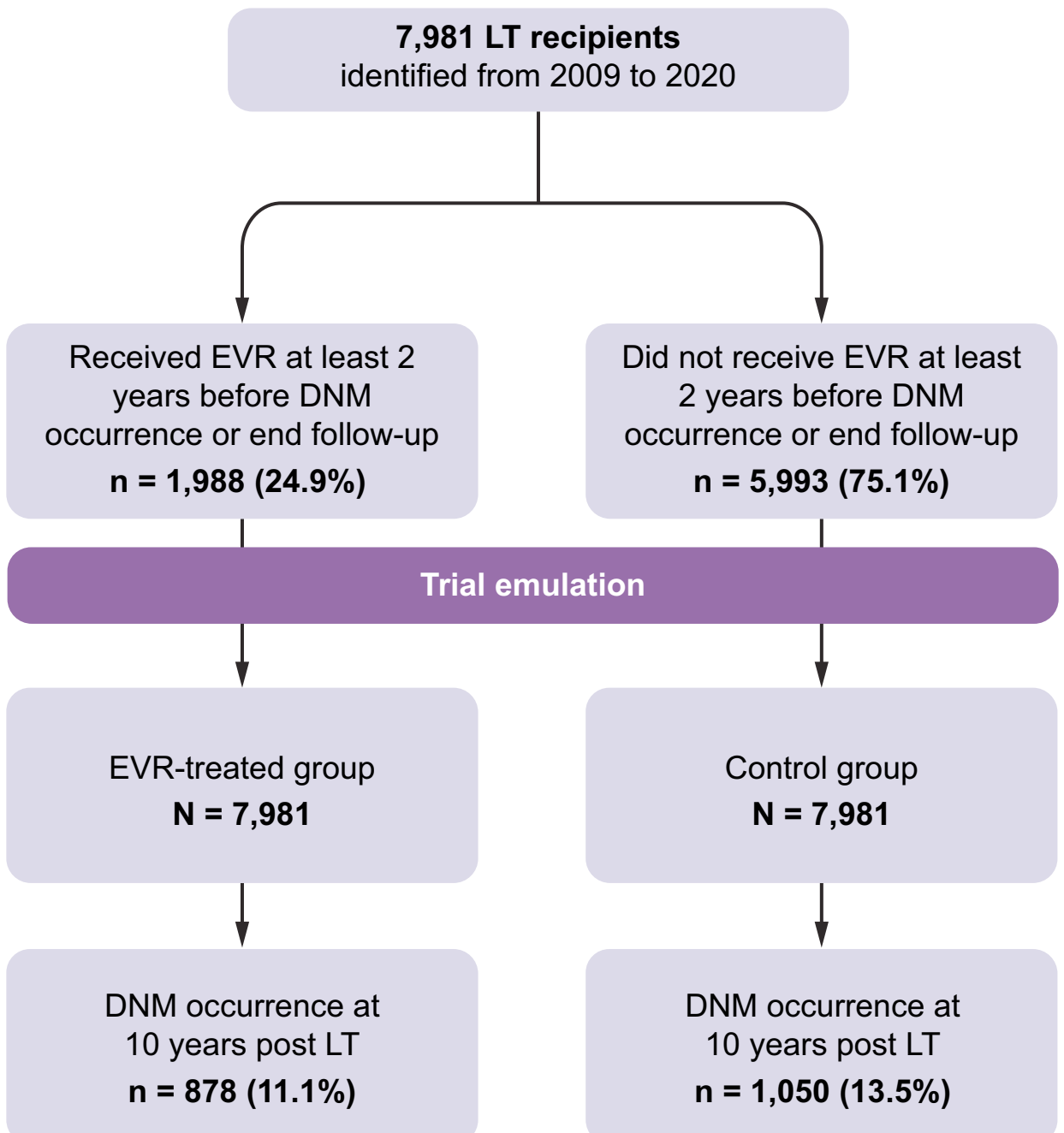

Fig. 1. Study flowchart. Among 7,981 liver transplant recipients, 2,256 patients (28.3%) received EVR during follow-up. Of these, 1,988 (24.9% of the total cohort) initiated EVR at least two years before DNM occurrence or censoring. DNM, *de novo* malignancies; EVR, everolimus.

Restricting analyses to the pre-2015 period yielded results consistent with the primary analysis for both DNM-free survival (HR 0.85, 95% CI 0.81–0.88) and overall survival (HR 1.04, 95% CI 0.92–1.10) (Fig. S6).

Analyses restricted to patients transplanted for HCC showed similar results, with an HR of 0.87 (95% CI, 0.72–0.90) for DNM occurrence and an HR of 0.99 (95% CI, 0.98–1.01) for overall survival (Fig. S7).

Sensitivity analyses using alternative gap definitions for treatment discontinuation (30, 60, and 180 days) produced estimates consistent with the main findings, with HRs for DNM occurrence ranging from 1.40 to 1.60 and for overall survival from 1.41 to 2.03 (Table S8).

Discussion

This study used emulated target trials to evaluate the association between EVR use and the risk of *de novo* malignancy after LT. EVR initiation was associated with a lower probability of DNM, whereas EVR discontinuation was associated with an increased risk of DNMs, and no association was observed with a concomitant discontinuation of tacrolimus.

The antitumor properties of mTOR inhibitors are established at doses higher than those used post-transplantation. At lower post-LT doses, EVR likely exerts its effects through immunomodulatory mechanisms rather than direct cytotoxicity. EVR did not confer uniform protection across DNM types. Reduced incidences were observed in bladder, lung, hematological, prostate, skin, and lymphoid cancers but not head and neck, colorectal, or gynecological cancers, suggesting tumor-specific differences in immune sensitivity.

Nearly half of the patients who initiated EVR discontinued this treatment during follow-up. Although the SNDS did not capture the reasons for discontinuation, one potential explanation concerns EVR-related adverse effects and immunosuppression minimization in stable recipients. The ETT-2 trial showed that continued EVR use was associated with a lower risk of DNM.

Tacrolimus discontinuation among EVR initiators was not associated with a detectable change in the DNM risk. However, this finding should be interpreted with caution. In clinical practice, EVR initiation is typically accompanied by tacrolimus minimization, and detailed pharmacokinetic data on tacrolimus exposure were not available in the SNDS database. The literature on liver transplantation indicates that the oncologic risk is more strongly related to the intensity and timing of calcineurin inhibitor exposure – particularly high tacrolimus levels soon after transplantation and cumulative exposure during the first post-transplant year – rather than to prescription status alone.[44,45] Consequently, the present analysis cannot exclude the possibility that the observed association between EVR exposure and lower DNM incidence partly reflected reduced calcineurin inhibitor exposure rather than a direct antineoplastic effect of EVR itself. In addition, most EVR-treated patients (81.8%) discontinued tacrolimus within the grace period, which reduced the contrast between continuation and discontinuation strategies and limited statistical power to detect differences between these groups. Taken together, these findings suggest that sustained EVR exposure may be associated with a lower probability of DNM after liver transplantation, but the relative contribution of EVR itself *vs.* concomitant calcineurin inhibitor minimization could not be determined in the absence of detailed exposure data.

While EVR initiation significantly reduced the risk of DNMs, this benefit did not translate into improved 10-year overall survival. This outcome is not necessarily discordant, given several contributing factors. Firstly, post-liver transplant mortality is multifactorial, with malignancies accounting for a limited proportion of deaths (approximately 30% in published series[46]), thereby limiting the overall impact of a moderate reduction in DNM incidence (HR 0.84). Moreover, overall survival is a non-specific endpoint that may lack the power to detect an indirect effect from a moderate reduction in DNM, particularly because only one-quarter of our cohort developed DNM. Secondly, the cancer types most effectively reduced by EVR (skin, prostate, hematologic, and lymphoid cancers) are generally associated with relatively favorable survival rates (53–72%). Conversely, the rates of cancers with the poorest prognosis, such as head and neck (24.4%), lung (27%), and bladder (30.1%), were either not reduced or only modestly affected by EVR. Finally, nearly half of the patients initiating EVR discontinued this treatment during follow-up, which likely attenuated any potential long-term survival benefits. Our inability to specifically assess cancer-related mortality, due to the inconsistent reporting of causes of death, further limited our understanding of this relationship.

The 10-year cumulative incidence of DNM (25.7%) was consistent with the findings of previous French studies, particularly regarding head and neck and skin cancers. The lower incidence of skin cancer in our cohort likely reflects stricter inclusion criteria that only captured invasive cancers requiring

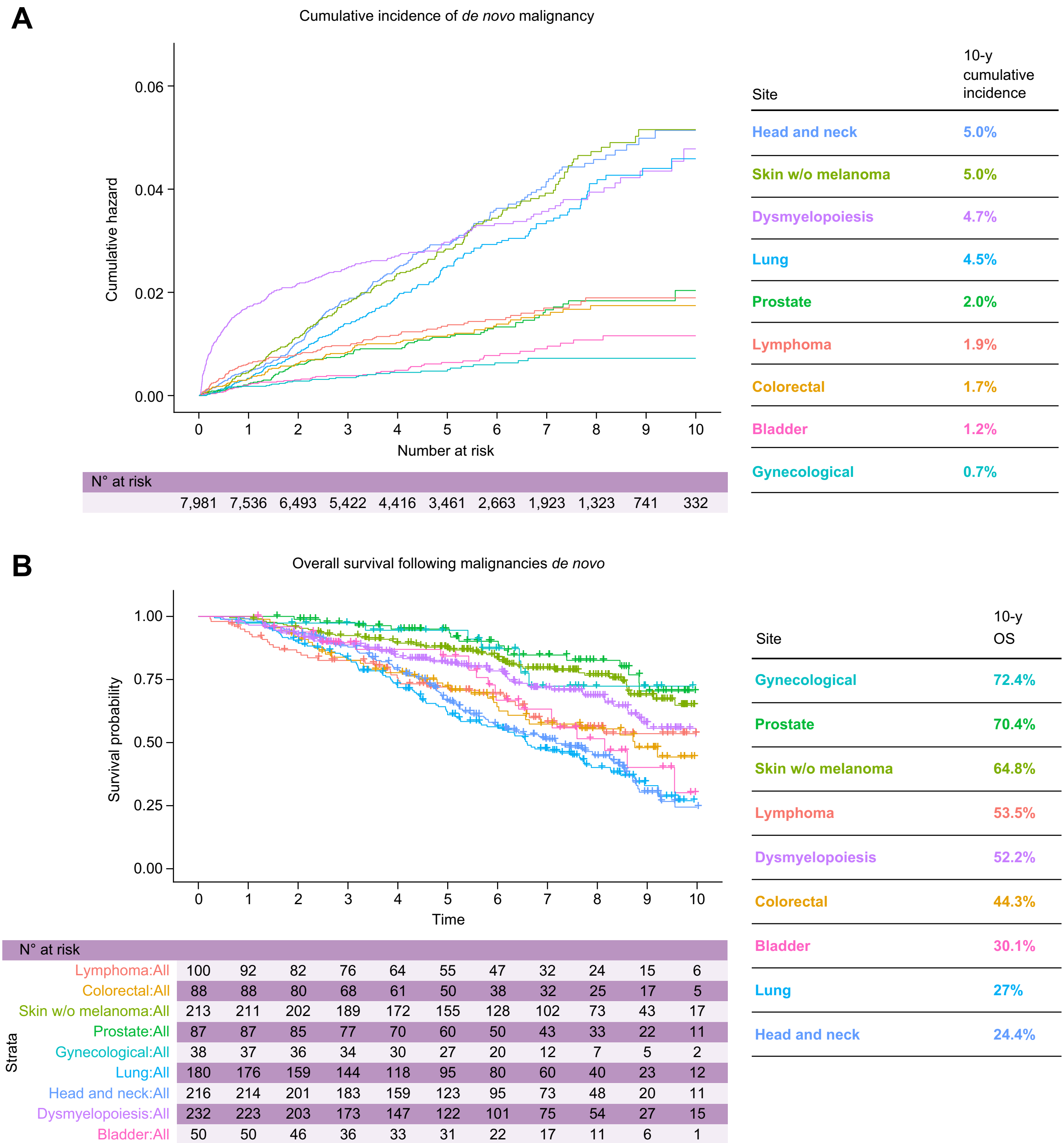

Fig. 2. Cumulative incidence of DNM after liver transplantation and 10-year overall survival following DNM. Five- and ten-year cumulative incidence estimates of DNM were stratified by cancer site. Head and neck, non-melanoma skin, myeloid neoplasms, and bladder cancers were the most frequent malignancies found at 10 years (A). Kaplan–Meier estimates of 10-year overall survival after DNM diagnosis are shown by cancer site; head and neck, lung, and bladder cancers exhibited the lowest survival rates (B). DNM, *de novo* malignancies; EVR, everolimus.

hospitalization. The relatively high incidence of hematological malignancies could be explained by coding practices in the administrative databases, whereby chronic hematological conditions – often diagnosed and managed in an outpatient setting – are recorded during hospitalizations related to liver transplantation or its follow-up. Survival rates after DNM diagnosis were also in line with findings in the literature, underscoring the long-term burden of malignancies after LT.[47–53]

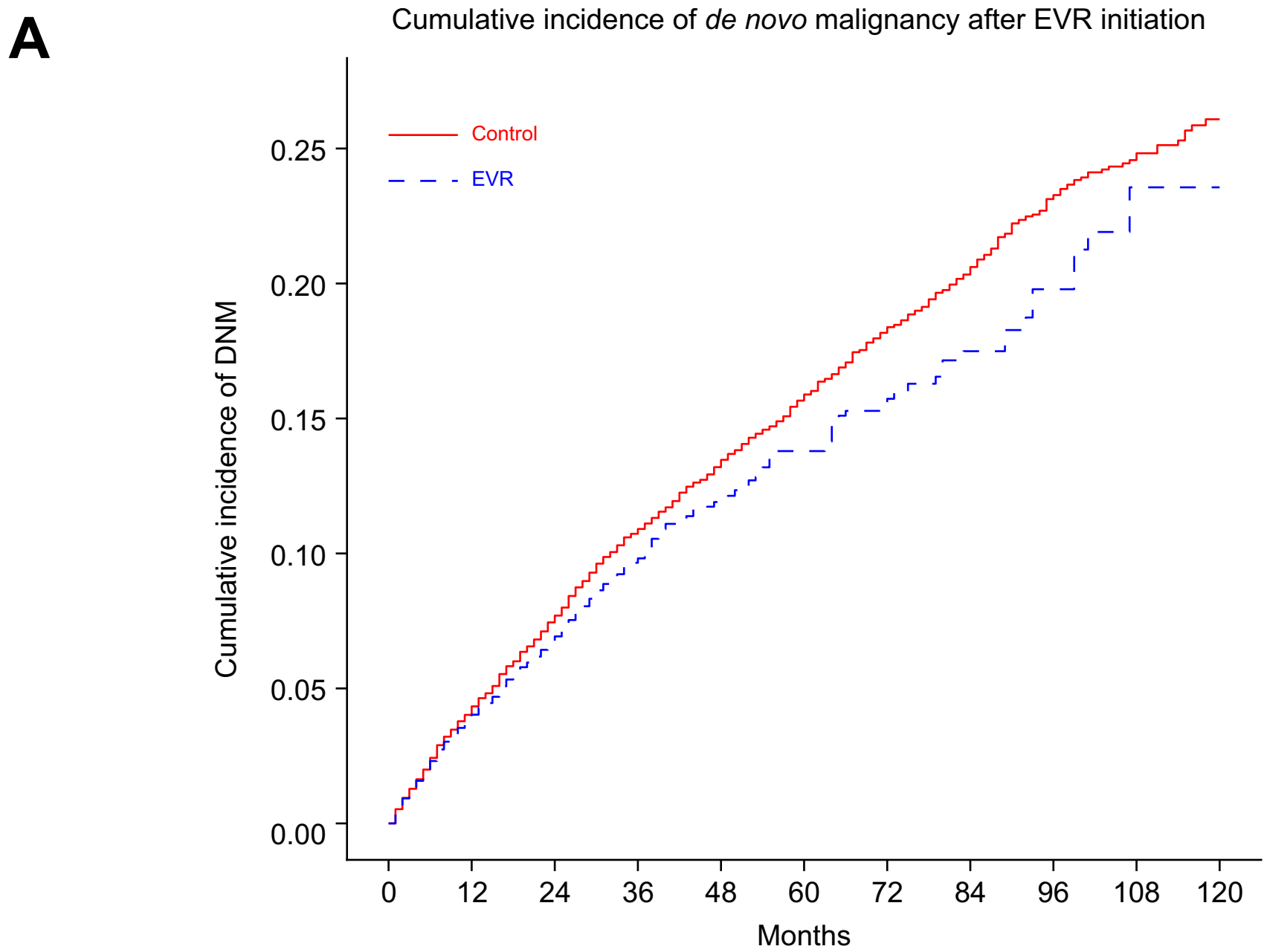

Control	7,981	7,525	6,391	5,255	4,317	3,440	2,704	2,030	1,457	849	403
EVR	7,981	7,556	6,550	5,466	4,028	3,107	2,156	1,289	757	403	218
Time (y)	0	1	2	3	4	5	6	7	8	9	10

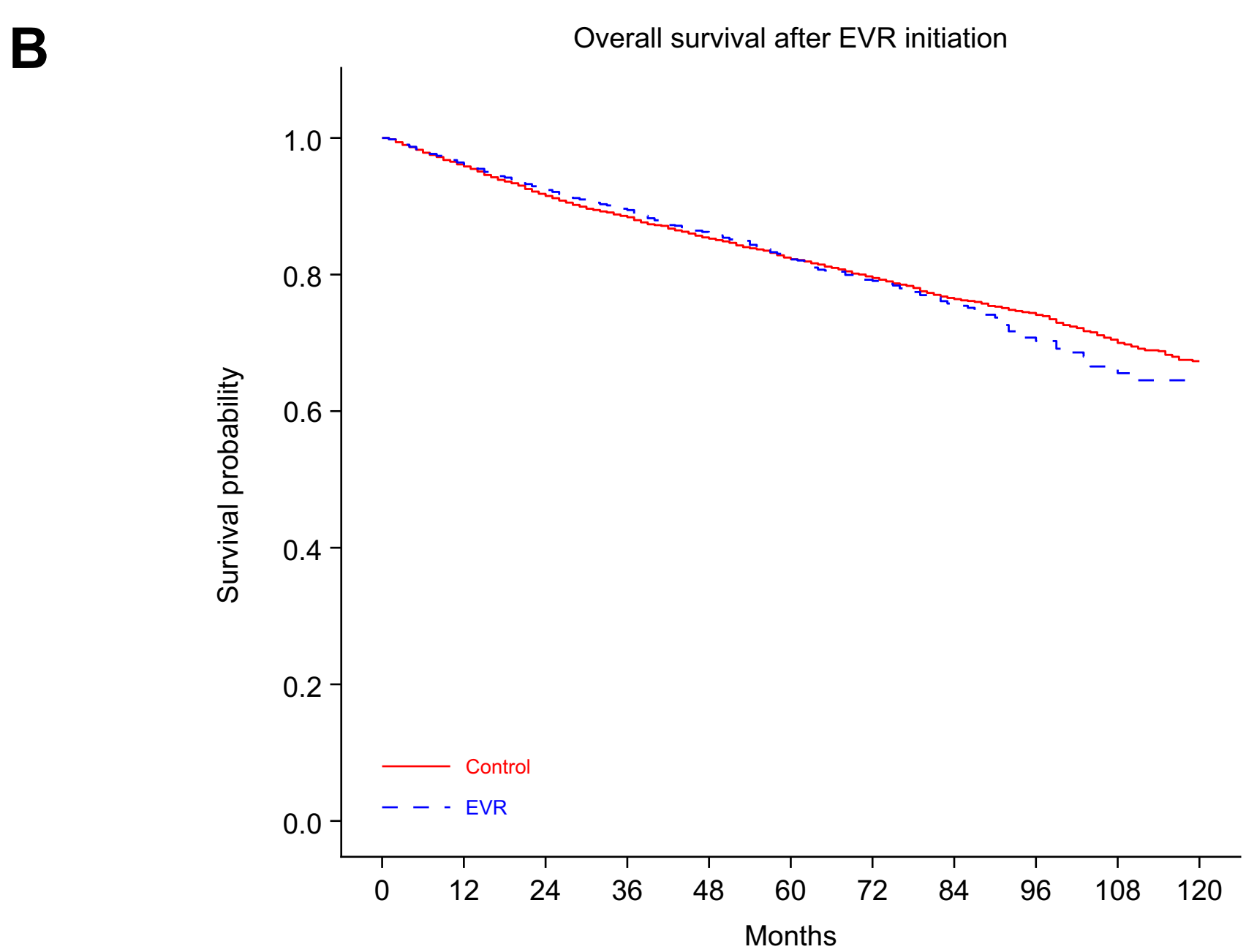

Control	7,981	7,525	6,391	5,255	4,317	3,440	2,704	2,030	1,457	849	403
EVR	7,981	7,556	6,550	5,466	4,028	3,107	2,156	1,289	757	403	218
Time (y)	0	1	2	3	4	5	6	7	8	9	10

Fig. 3. Cumulative probability of DNM and overall survival in emulated target trial 1 (EVR initiation *vs.* No EVR). Cumulative probability of DNM occurrence (A) and overall survival (B) over 10 years, comparing patients who initiated EVR *vs.* those who did not. Estimates were based on a 3-year grace period and a 2-year lag time. A total of 1,988 patients who initiated EVR within this framework were included in the treatment arm. Nelson–Aalen and Kaplan–Meier curves were weighted using the cloning–censoring–weighting method. EVR initiation was associated with a reduced risk of DNM (HR 0.84; 95% CI 0.79–0.88). Ten-year overall survival was similar between the arms (HR 1.00; 95% CI 0.96–1.04). DNM, *de novo* malignancies; EVR, everolimus; HR, hazard ratio.

A

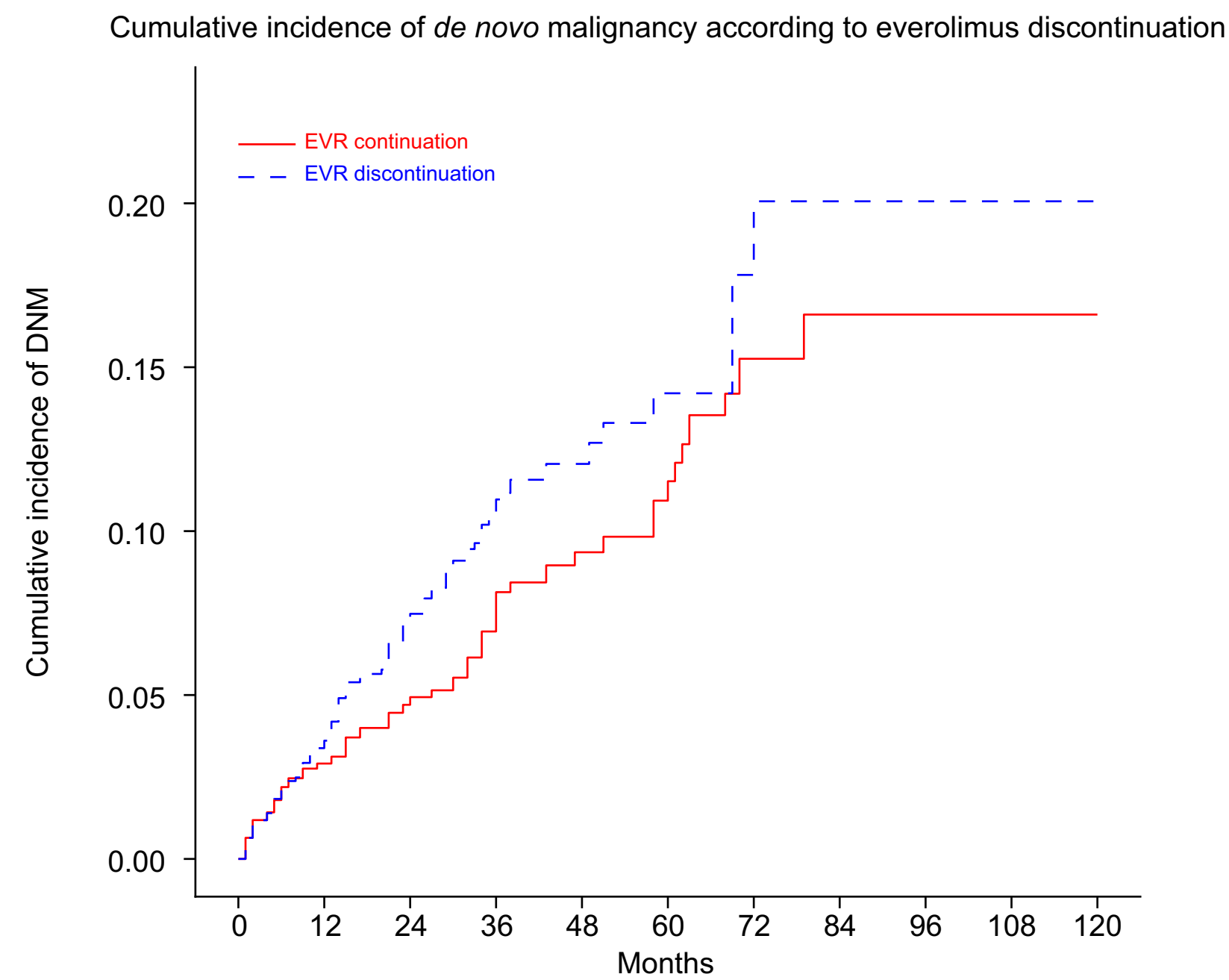

EVR continuation	1,988	1,531	1,345	1,214	945	651	392	195	86	41	15
EVR discontinuation	1,988	1,591	1,187	895	584	335	172	89	49	27	4
Time (y)	0	1	2	3	4	5	6	7	8	9	10

B

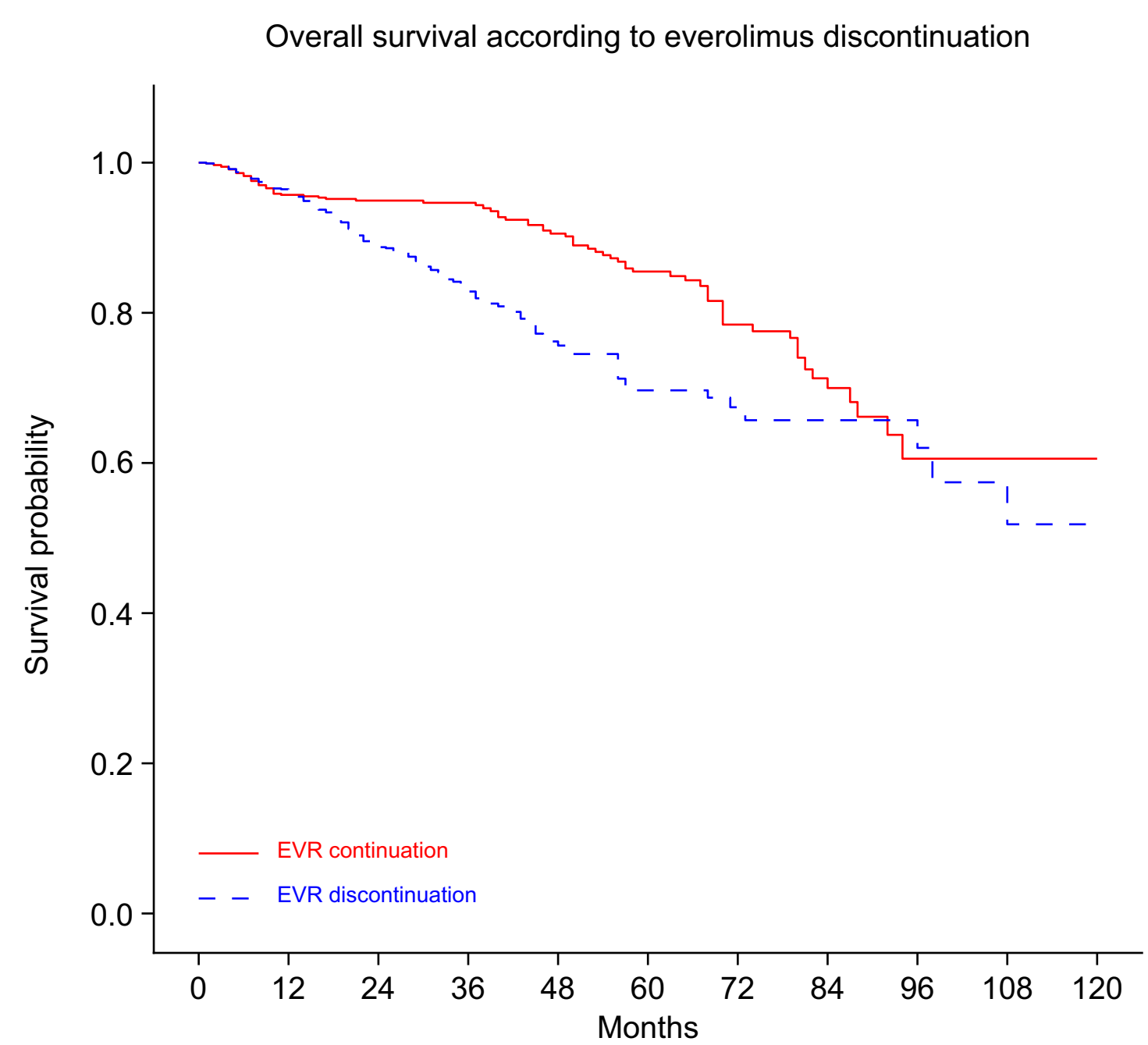

EVR continuation	1,988	1,531	1,345	1,214	945	651	392	195	86	41	15
EVR discontinuation	1,988	1,591	1,187	895	584	335	172	89	49	27	4
Time (y)	0	1	2	3	4	5	6	7	8	9	10

Fig. 4. Cumulative probability of DNM and overall survival in emulated target trial 2 (EVR continuation *vs.* discontinuation). Cumulative probability of DNM occurrence (A), and 10-year overall survival among patients who continued EVR *vs.* those who discontinued it after initiation (n = 1,988). Analyses were based on a 3-year grace period and a 2-year lag time. A total of 675 patients were included in the EVR discontinuation arm. Nelson–Aalen and Kaplan–Meier curves were weighted using the cloning–censoring–weighting method. EVR discontinuation was associated with an increased risk of DNM (HR 1.34; 95% CI 1.21–1.45) and showed no significant effect on overall survival (HR 1.00; 95% CI 0.88–1.13). DNM, *de novo* malignancies; EVR, everolimus; HR, hazard ratio.

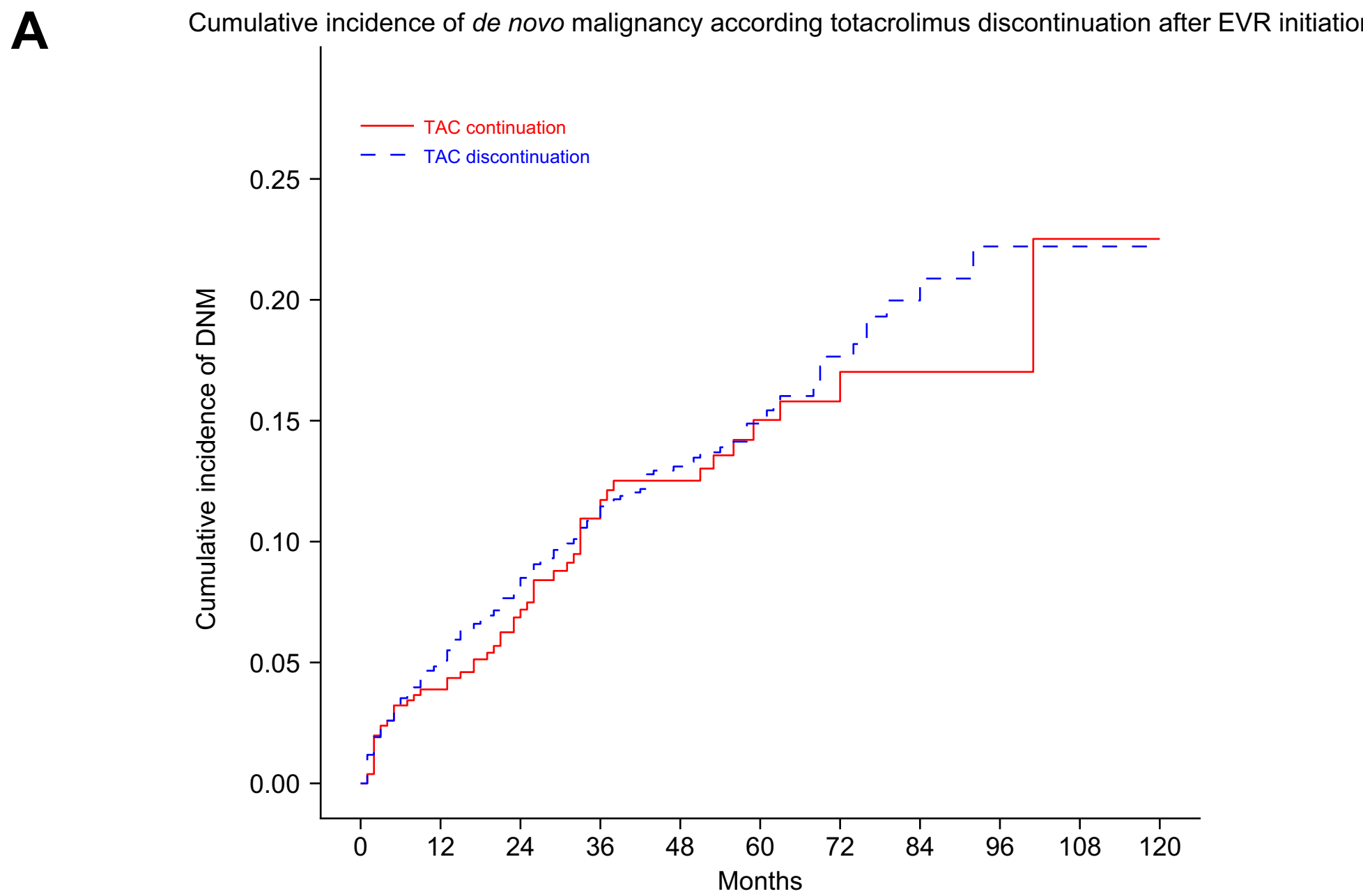

TAC continuation	1,988	1,687	1,339	1,021	739	434	222	121	53	21	14
TAC discontinuation	1,988	1,340	1,106	897	718	540	344	233	131	57	0
Time (y)	0	1	2	3	4	5	6	7	8	9	10

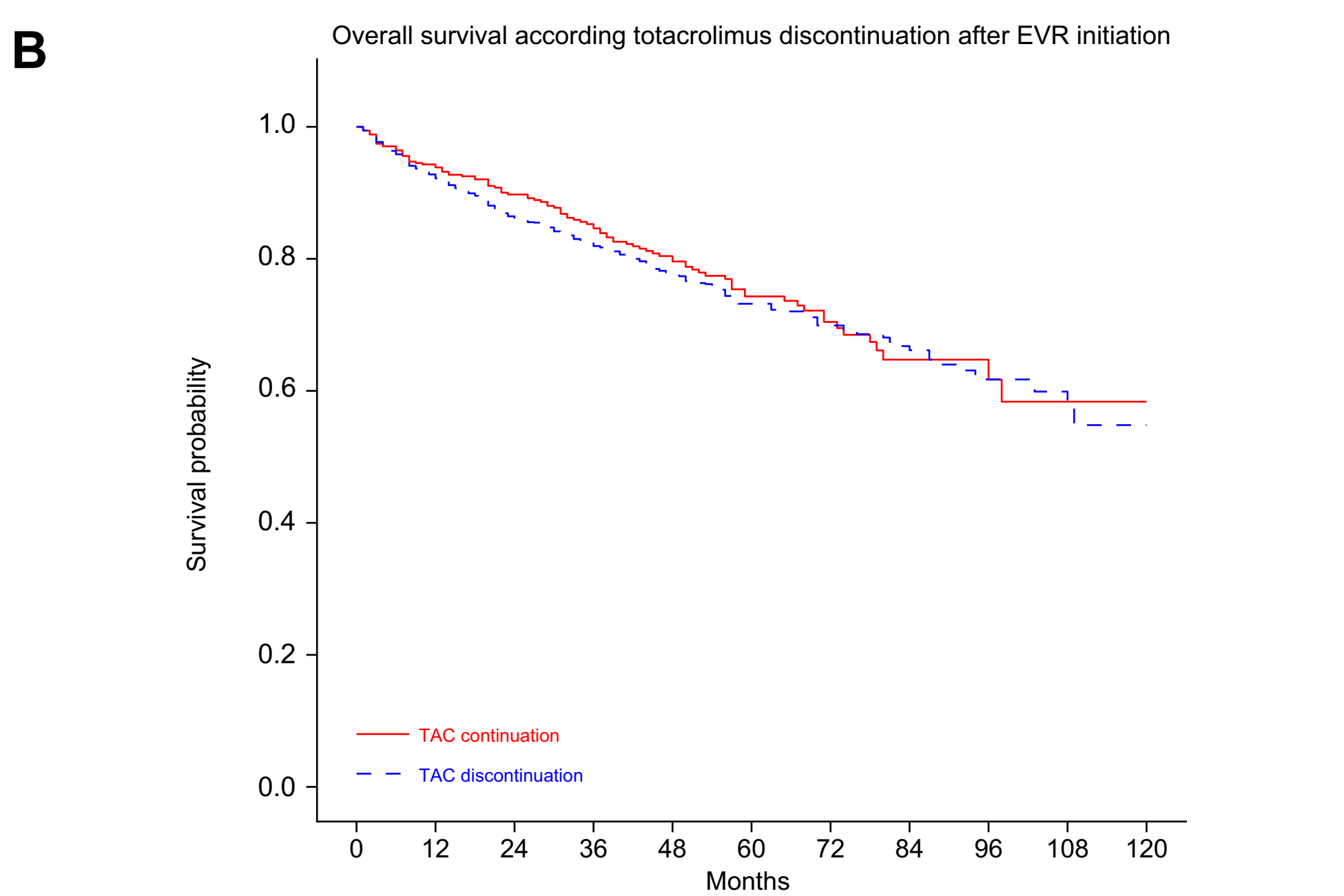

TAC continuation	1,988	1,687	1,339	1,021	739	434	222	121	53	21	14
TAC discontinuation	1,988	1,340	1,106	897	718	540	344	233	131	57	0
Time (y)	0	1	2	3	4	5	6	7	8	9	10

Fig. 5. Cumulative probability of DNM and overall survival in emulated target trial 3 (discontinuation of tacrolimus after EVR initiation). Cumulative probability of DNM occurrence (A) and overall survival (B) over 10 years among EVR initiators (n = 1,988), comparing patients who continued TAC *vs.* those who discontinued it. Analyses used a 3-year grace period and a 2-year lag time. A total of 1,626 patients were included in the TAC discontinuation arm. Nelson–Aalen and Kaplan–Meier curves were weighted using the cloning–censoring–weighting method. The 10-year risk of DNM was similar between the arms (HR 1.03; 95% CI 0.83–1.21), as was overall survival (HR 1.00; 95% CI 0.92–1.07). DNM, *de novo* malignancies; EVR, everolimus; HR, hazard ratio; TAC, tacrolimus.

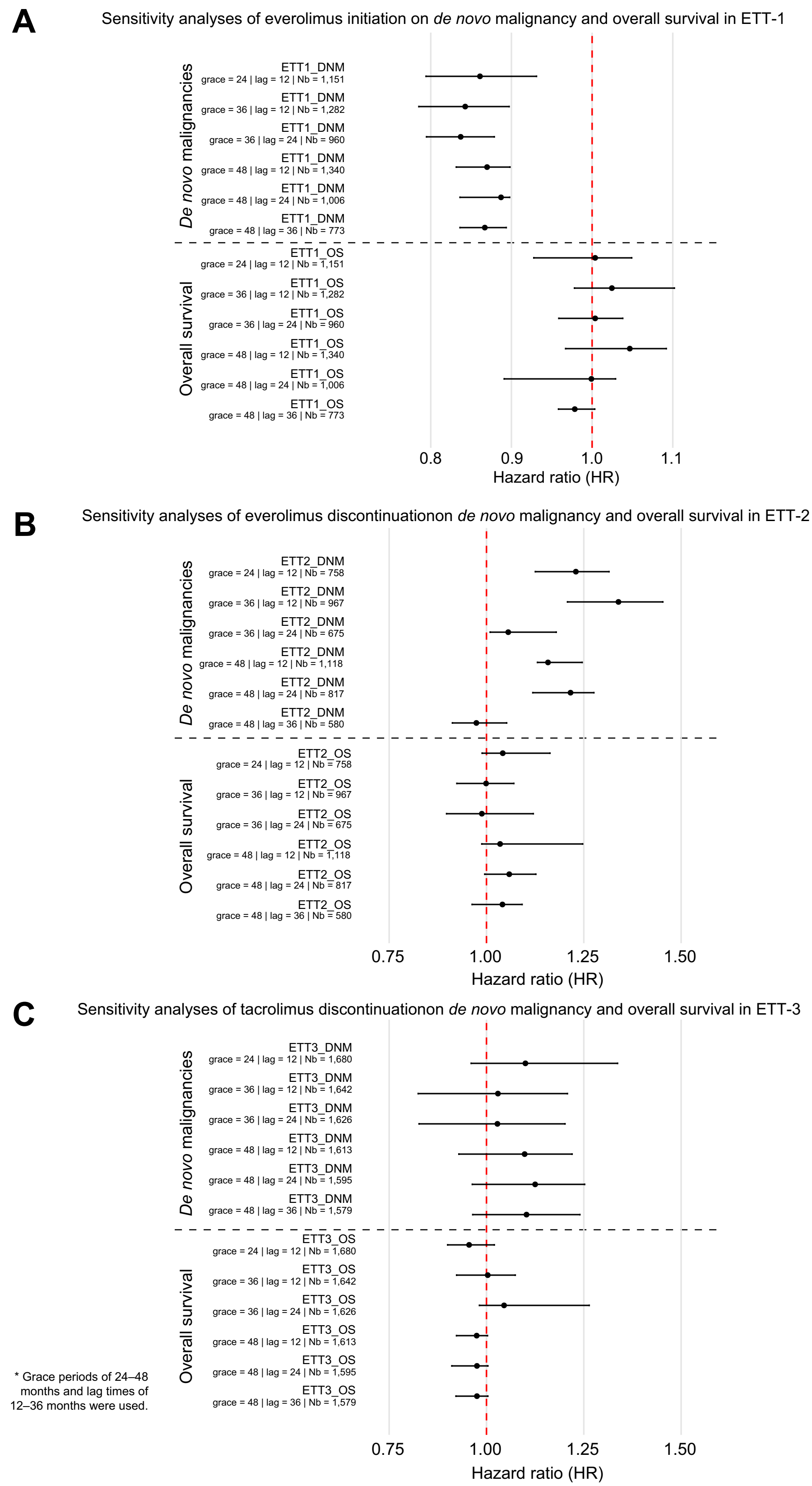

Fig. 6. Sensitivity analysis across different grace period and lag time scenarios. Six parameter combinations were evaluated for each emulated target trial, varying the grace period (24, 36, or 48 months) and lag time (12, 24, or 36 months), with the constraint that grace ≠ lag time. All analyses used a 10-year follow-up period. Across all combinations, ETT-1 showed a consistent protective effect of EVR on DNM and no effect on overall survival (A). ETT-2 consistently showed an increased DNM risk associated with EVR discontinuation, except for the combination of grace = 48 and lag = 36 months (B). For ETT-3, all parameter combinations yielded similar results for DNM and overall survival, indicating no measurable influence of TAC (C). DNM, *de novo* malignancies; ETT, emulated target trial; EVR, everolimus; TAC, tacrolimus.

We applied a CCW framework that relied on three assumptions: positivity (non-zero probability of EVR treatment), consistency (well-defined exposure), and exchangeability (addressed through CCW). Each emulated trial demonstrated adequate covariate balance, and the findings were robust across parameter variations. The SNDS database offers a comprehensive, longitudinal follow-up of the French population, enabling an accurate assessment of drug exposure, comorbidities, and outcomes. Its completeness ensures strong external validity, consistent with international recommendations for real-world evidence in drug evaluation.[51,52]

This study had some limitations. Only cancers requiring hospitalization were captured, potentially missing outpatient diagnoses, although the use of a lag period helped to mitigate this risk. In the SNDS, outpatient procedures recorded in the DCIR enable the identification of dermatologic excisions and oncologic treatments performed in ambulatory settings, although diagnostic codes do not always specify the underlying malignancy. Consequently, malignancies commonly diagnosed and treated in outpatient settings (particularly skin cancers) may not have been fully captured. Histological subtypes and TNM staging were unavailable. Cause of death coding was inconsistent, and this prevented any determination of cancer-specific mortality. Donor–recipient linkages were not available, limiting any evaluation of donor-related factors. Information on induction therapy, the treatment of acute rejection, or exposure to lymphocyte-depleting agents (known determinants of post-transplant lymphoproliferative disorders) was not available in the SNDS database. As with any studies using administrative claims data, misclassification was possible, although prior validations supported the accuracy of the SNDS in terms of identifying cancer.[53] Medication adherence could not be confirmed, potentially underestimating the effects of initiation or discontinuation. Unmeasured confounding within the grace period could have weakened causal interpretation, although such confounders would affect both drug initiation and discontinuation decisions.

In conclusion, *de novo* malignancies represent a major long-term challenge after liver transplantation, underscoring the importance of sustained cancer surveillance, particularly for tumors amenable to early detection. Using an emulated target trial framework applied to a nationwide cohort, this study suggests that sustained exposure to EVR is associated with a lower incidence of DNMs. These findings support immunosuppressive strategies that incorporate calcineurin inhibitor minimization, including EVR-based regimens, as a strategy to potentially reduce the long-term oncologic risk after liver transplantation.

Affiliations

[1]*INSERM, Université Paris-Saclay, UMR-S 1193, Villejuif, France;* [2]*AP-HP Hôpital Paul-Brousse, Centre Hépato-Biliaire, Villejuif, France;* [3]*Université Paris-Saclay, Inserm, Physiopathogénèse et Traitement des Maladies du Foie, Villejuif, France;* [4]*France FHU Hepatinov, Villejuif, France;* [5]*Department of Public Health, University of Copenhagen, Denmark;* [6]*Clinical Research Institute, Montpellier University, France*

Abbreviations

CCMP, common classification of medical procedures; CCW, cloning-censoring-weighting; CNI, calcineurin inhibitors; DCIR, Datamart de Consommation Inter-Régimes (National Health Insurance Reimbursement Database); DNMs, *de novo* malignancies; ETT, emulated target trials; EVR, everolimus; HCC, hepatocellular carcinoma; LT, liver transplantation; mTOR, mammalian target of rapamycin; MMF, mycophenolate mofetil; NNH, number needed to harm; NNT, number needed to treat; PMSI, Programme de médicalisation des systèmes d'information (French Hospital Discharge Database); SNDS, French National Health Data System (Système National de Données de Santé); TAC, tacrolimus.

Financial support

The authors declare that no financial support was provided for this study or compilation of this manuscript.

Conflict of interest

The authors declare no conflicts of interest.

Please refer to the accompanying ICMJE disclosure forms for further details.

Authors' contributions

Ilias Kounis, Tri-Long Nguyen, Paul Landais and Cyrille Feray contributed to the conception and design of this study and to preparation and finalization of the manuscript. Ilias Kounis, Tri-Long Nguyen, Paul Landais and Cyrille Feray performed the statistical analysis. Christophe Desterke, Nathalie Goutte, Paul Landais and Cyrille Feray participated in data collection. All authors critically reviewed the manuscript for important intellectual content and approved the final draft for submission.

Data availability

According to French regulations on data protection, the authors cannot publicly release data from the French National Health Data System (*Système National de Données de Santé* - SNDS). However, any person or structure, public or private, for profit or non-profit, can access SNDS data following authorization from the French Data Protection Office (Commission *Nationale de l'Informatique et des Libertés*) to carry out a study or an evaluation of public interest.

Ethical registration

The project was approved by the French National Commission for Information and Liberties (CNIL) N°919274.

Prior presentations

These findings were presented at the International Liver Transplantation Society (ILTS) Congress in Singapore, May 2025, and at the European Association for the Study of the Liver (EASL) Congress in Milan, June 2024.

Supplementary data

Supplementary data to this article can be found online at https://doi.org/10.1016/j.jhepr.2026.101934.

References

[1] Engels EA, Pfeiffer RM, Fraumeni Jr JF, et al. Spectrum of cancer risk among US solid organ transplant recipients. JAMA 2011;306:1891–1901.

[2] Colmenero J, Tabrizian P, Bhangui P, et al. De novo malignancy after liver transplantation: risk assessment, prevention, and management-guidelines from the ILTS-SETH Consensus Conference. Transplantation 2022;106:e30–e45.

[3] Altieri M, Sérée O, Lobbedez T, et al. Risk factors of de novo malignancies after liver transplantation: a French national study on 11004 adult patients. Clin Res Hepatol Gastroenterol 2021;45:101514.

[4] Park B, Yoon J, Choi D, et al. De novo cancer incidence after kidney and liver transplantation: results from a nationwide population based data. Sci Rep 2019;9:17202.

[5] Poudel S, Gupta S, Saigal S. Basics and art of immunosuppression in liver transplantation. J Clin Exp Hepatol 2024;14:101345.

[6] Rodríguez-Perálvarez M, Germani G, Darius T, et al. Tacrolimus trough levels, rejection and renal impairment in liver transplantation: a systematic review and meta-analysis. Am J Transpl 2012;12:2797–2814.

[7] Ojo AO, Held PJ, Port FK, et al. Chronic renal failure after transplantation of a nonrenal organ. N Engl J Med 2003;349:931–940.
[8] Kulbat A, Richter K, Stefura T, et al. Systematic review of calcineurin inhibitors and incidence of skin malignancies after kidney transplantation in adult patients: a study of 309,551 cases. Curr Oncol 2023;30:5727–5737.
[9] Beckebaum S, Cicinnati VR, Radtke A, et al. Calcineurin inhibitors in liver transplantation - still champions or threatened by serious competitors? Liver Int 2013;33:656–665.
[10] Saliba F, Dharancy S, Salamé E, et al. Time to conversion to an everolimus-based regimen: renal outcomes in liver transplant recipients from the EVEROLIVER Registry. Liver Transpl 2020;26:1465–1476.
[11] Masetti M, Montalti R, Rompianesi G, et al. Early withdrawal of calcineurin inhibitors and everolimus monotherapy in de novo liver transplant recipients preserves renal function. Am J Transpl 2010;10:2252–2262.
[12] Berger SP, Sommerer C, Witzke O, et al. Two-year outcomes in de novo renal transplant recipients receiving everolimus-facilitated calcineurin inhibitor reduction regimen from the TRANSFORM study. Am J Transpl 2019;19:3018–3034.
[13] Fischer L, Klempnauer J, Beckebaum S, et al. A randomized, controlled study to assess the conversion from calcineurin-inhibitors to everolimus after liver transplantation–PROTECT. Am J Transpl 2012;12:1855–1865.
[14] Motzer RJ, Escudier B, Oudard S, et al. Efficacy of everolimus in advanced renal cell carcinoma: a double-blind, randomised, placebo-controlled phase III trial. Lancet 2008;372:449–456.
[15] Baselga J, Campone M, Piccart M, et al. Everolimus in postmenopausal hormone-receptor-positive advanced breast cancer. N Engl J Med 2012;366:520–529.
[16] Yao JC, Shah MH, Ito T, et al. Everolimus for advanced pancreatic neuroendocrine tumors. N Engl J Med 2011;364:514–523.
[17] Samuel D, De Martin E, Berg T, et al. European Association for the Study of the Liver. EASL clinical practice guidelines on liver transplantation. J Hepatol 2024;81:1040–1086.
[18] Arena C, Bizzoca ME, Caponio VCA, et al. Everolimus therapy and side effects: a systematic review and meta analysis. Int J Oncol 2021;59:54.
[19] Liang W, Wang D, Ling X, et al. Sirolimus-based immunosuppression in liver transplantation for hepatocellular carcinoma: a meta-analysis. Liver Transpl 2012;18:62–69.
[20] Menon KV, Hakeem AR, Heaton ND. Meta-analysis: recurrence and survival following the use of sirolimus in liver transplantation for hepatocellular carcinoma. Aliment Pharmacol Ther 2013;37:411–419.
[21] Cholongitas E, Mamou C, Rodríguez-Castro KI, et al. Mammalian target of rapamycin inhibitors are associated with lower rates of hepatocellular carcinoma recurrence after liver transplantation: a systematic review. Transpl Int 2014;27:1039–1049.
[22] Zhang ZH, Li LX, Li P, et al. Sirolimus in liver transplant recipients with hepatocellular carcinoma: an updated meta-analysis. J Invest Surg 2019;32:632–641.
[23] Grigg SE, Sarri GL, Gow PJ, et al. Systematic review with meta-analysis: sirolimus- or everolimus-based immunosuppression following liver transplantation for hepatocellular carcinoma. Aliment Pharmacol Ther 2019;49:1260–1273.
[24] Yan X, Huang S, Yang Y, et al. Sirolimus or everolimus improves survival after liver transplantation for hepatocellular carcinoma: a systematic review and meta-analysis. Liver Transpl 2022;28:1063–1077.
[25] Jang SC, Kim GA, Lim YS, et al. Association between everolimus combination therapy and cancer risk after liver transplantation: a nationwide population-based quasi-cohort study. Am J Transpl 2025;25:1285–1295.
[26] Geissler EK, Schnitzbauer AA, Zülke C, et al. Sirolimus use in liver transplant recipients with hepatocellular carcinoma: a randomized, multicenter, open-label phase 3 trial. Transplantation 2016;100:116–125.
[27] Rodríguez-Perálvarez M, Colmenero J, González A, et al. Cumulative exposure to tacrolimus and incidence of cancer after liver transplantation. Am J Transpl 2022;22:1671–1682.
[28] Knoll GA, Kokolo MB, Mallick R, et al. Effect of sirolimus on malignancy and survival after kidney transplantation: systematic review and meta-analysis of individual patient data. BMJ 2014;349:g6679.
[29] Yanik EL, Siddiqui K, Engels EA. Sirolimus effects on cancer incidence after kidney transplantation: a meta-analysis. Cancer Med 2015;4:1448–1459.
[30] Sedgwick P. Convenience sampling. BMJ 2013;347:f6304.
[31] Hernán MA, Sauer BC, Hernández-Díaz S, et al. Specifying a target trial prevents immortal time bias and other self-inflicted injuries in observational analyses. J Clin Epidemiol 2016;79:70–75.
[32] Hernán MA, Robins JM. Using big data to emulate a target trial when a randomized trial is not available. Am J Epidemiol 2016;183:758–764.
[33] Hernán MA. How to estimate the effect of treatment duration on survival outcomes using observational data. BMJ 2018;360:k182.
[34] Gaber CE, Ghazarian AA, Strassle PD, et al. De-mystifying the clone-censor-weight method for causal research using observational data: a primer for cancer researchers. Cancer Med 2024;13:e70461.
[35] Dumas E, Jochum F, Coussy F, et al. Explaining the relationships between age, endocrine therapy persistence, and risk of recurrence in hormone receptor-positive early breast cancer: a nationwide cohort study. J Clin Oncol 2025;43:1863–1874.
[36] Chapitre Ier. Système national des données de santé (Articles L1461-1 à L1461-7) - Légifrance.. Available from: https://www.legifrance.gouv.fr/codes/section_lc/LEGITEXT000006072665/LEGISCTA000031923877/.
[37] Bezin J, Duong M, Lassalle R, et al. The national healthcare system claims databases in France, SNIIRAM and EGB: powerful tools for pharmacoepidemiology. Pharmacoepidemiol Drug Saf 2017;26:954–962.
[38] Tuppin P, Rudant J, Constantinou P, et al. Value of a national administrative database to guide public decisions: from the système national d'information interrégimes de l'Assurance Maladie (SNIIRAM) to the système national des données de santé (SNDS) in France. Rev Epidemiol Sante Publique 2017;65:S149–S167.
[39] International Classification of Diseases (ICD). Available from: https://www.who.int/standards/classifications/classification-of-diseases.
[40] Métropole - Indice de défavorisation sociale (FDep) à l'échelle de l'IRIS - Inserm. ATLASANTE. Available from:: https://catalogue.atlasante.fr/geonetwork/srv/api/records/8ba3a966-fda5-4147-ba45-f6f9fd899c09.
[41] Maringe C, Benitez Majano S, Exarchakou A, et al. Reflection on modern methods: trial emulation in the presence of immortal-time bias. Assessing the benefit of major surgery for elderly lung cancer patients using observational data. Int J Epidemiol 2020;49:1719–1729.
[42] VanderWeele TJ, Ding P. Sensitivity analysis in observational research: introducing the E-Value. Ann Intern Med 2017;167:268–274.
[43] Altman DG, Andersen PK. Calculating the number needed to treat for trials where the outcome is time to an event. BMJ 1999;319:1492–1495.
[44] Rodríguez-Perálvarez M, Tsochatzis E, Naveas MC, et al. Reduced exposure to calcineurin inhibitors early after liver transplantation prevents recurrence of hepatocellular carcinoma. J Hepatol 2013;59:1193–1199.
[45] Carenco C, Assenat E, Faure S, et al. Tacrolimus and the risk of solid cancers after liver transplant: a dose effect relationship. Am J Transpl 2015;15:678–686.
[46] Watt KD, Pedersen RA, Kremers WK, et al. Evolution of causes and risk factors for mortality post-liver transplant: results of the NIDDK long-term follow-up study. Am J Transpl 2010;10:1420–1427.
[47] Lucidi C, Biolato M, Lai Q, et al. Cumulative incidence of solid and hematological de novo malignancy after liver transplantation in a multicentre cohort. Ann Hepatol 2021;24:100309.
[48] Duvoux C, Delacroix I, Richardet JP, et al. Increased incidence of oropharyngeal squamous cell carcinomas after liver transplantation for alcoholic cirrhosis. Transplantation 1999;67:418–421.
[49] Mithoefer AB, Supran S, Freeman RB. Risk factors associated with the development of skin cancer after liver transplantation. Liver Transpl 2002;8:939–944.
[50] Nordin A, Åberg F, Pukkala E, et al. Decreasing incidence of cancer after liver transplantation-A Nordic population-based study over 3 decades. Am J Transpl 2018;18:952–963.
[51] Finkenstedt A, Graziadei IW, Oberaigner W, et al. Extensive surveillance promotes early diagnosis and improved survival of de novo malignancies in liver transplant recipients. Am J Transpl 2009;9:2355–2361.
[52] Kauffman HM, Cherikh WS, Cheng Y, et al. Maintenance immunosuppression with target-of-rapamycin inhibitors is associated with a reduced incidence of de novo malignancies. Transplantation 2005;80:883–889.
[53] Ajrouche A, Estellat C, De Rycke Y, et al. Evaluation of algorithms to identify incident cancer cases by using French health administrative databases. Pharmacoepidemiol Drug Saf 2017;26:935–944.

Keywords: mTOR inhibitor; Everolimus; EVR; liver transplantation; immunosuppression; *de novo* malignancies.
Received 30 January 2026; received in revised form 15 June 2026; accepted 21 June 2026; Available online xxx

Journal of Hepatology, Volume *8*

Supplemental information

Everolimus and *de novo* malignancies after liver transplantation: A nationwide emulated trial using real-world data

Ilias Kounis, Tri-Long Nguyen, Christophe Desterke, Nathalie Goutte, Didier Samuel, Daniel Azoulay, Eric Vibert, Faouzi Saliba, Audrey Coilly, Paul Landais, and Cyrille Féray

Everolimus and *de novo* malignancies after liver transplantation: A nationwide emulated trial using real-world data

Ilias Kounis, Tri-Long Nguyen, Christophe Desterke, Nathalie Goutte, Didier Samuel, Daniel Azoulay, Eric Vibert, Faouzi Saliba, Audrey Coilly, Paul Landais, Cyrille Féray

Table of contents

Supplementary figure legends.....2
Supplementary table legends.....3
Supplementary figures.....4
Supplementary tables.....8

Supplementary figure legends

Fig. S1. Illustration of patient trajectories and their corresponding cloned treatment strategies in a target trial emulation of Everolimus (EVR) in terms of preventing *de novo* malignancies after liver transplantation. Each real patient contributed two clones at baseline, randomly assigned to either the "EVR strategy" or the "Control strategy". The grace period (vertical dashed blue line) represents the window allowed for EVR initiation. Patients were followed from transplantation until *de novo* malignancy (DNM), administrative censoring, loss to follow-up, or death (all collapsed into censoring). As an illustration, we display nine representative scenarios: Scenarios 1A–1C: EVR initiated within the grace period. The Control clones are censored at the time of EVR initiation in the real patient (protocol deviation). Scenarios 2A–2D: No EVR initiated during the grace period. The EVR clones are censored at the end of the grace period. Scenarios 3A–3B: Late initiation of EVR (after the grace period). The Treated clones are censored at EVR initiation.

Fig. S2. Directed acyclic graph of the causal structure between Everolimus treatment and *de novo* malignancy or overall survival. Age and sex, comorbidities and lifestyle factors (tobacco and alcohol use), socioeconomic status, underlying liver disease and liver function before transplantation, baseline kidney function, and pre-transplant malignancy were considered baseline confounders of the association between Everolimus treatment and *de novo* malignancy or overall survival. Everolimus may also influence the recurrence of post-transplant chronic kidney disease and hepatocellular carcinoma (HCC), which are considered post-transplant mediators and were therefore not included in the adjustment set.

Fig. S3: Covariate balance before and after inverse probability weighting. Absolute standardized mean differences (SMDs) are shown for baseline characteristics before (red circles) and after (blue triangles) inverse probability treatment weighting (IPTW). Vertical dashed lines indicate SMD thresholds of 0.1, below which covariates are considered to be well balanced between treatment arms. After weighting, all covariates achieved adequate balance, supporting a successful reduction of measured confounding.

Fig. S4: Site-Specific Cumulative Incidence of *De Novo* Malignancies. Cumulative incidence curves for DNM by cancer site, comparing patients who initiated EVR versus those who did not. The analysis was based on a 3-year grace period and 2-year lag time. Site-specific curves highlight the differences in EVR-associated risk across tumor types, including bladder, hematologic, lung, prostate, skin, lymphoma, head and neck, colorectal, and gynecological cancers.

Fig. S5: Sensitivity analysis using zero lag time for ETT1 and ETT2.

Fig. S6: Cumulative incidence of de novo malignancy after everolimus initiation for patients transplanted before end of 2015 with 3-year grace period and 2-year lag time

Fig. S7: Cumulative incidence of de novo malignancy after everolimus initiation for patients transplanted for HCC with 3-year grace period and 2-year lag time.

Supplementary table legends

Table S1: Algorithm used to identify liver transplant recipients from the French national healthcare database.

Table S2: List of ICD-10 codes used to define cancer diagnoses for DNM classification.

Table S3: CCMP procedure codes used to confirm the diagnosis of DNM.

Table S4: Summary of protocol components of the emulated target trials.

Table S5: Multivariate Generalized Additive Model (GAM). This approach estimates adjusted effects for each covariate on the hazard of death or retransplantation. This table reports the estimated coefficients (Estimate), standard errors (SE), z-values and corresponding p-values. Variables with $p < 0.05$ were considered to be statistically significant.

Table S6: Covariate balance before and after inverse probability of censoring weighting.

Table S7: Effects of treatments in trials ETT-1 to ETT-3: risk difference (RD), hazard ratio (HR), number needed to treat/harm (NNT/NNH), and E-value.

Table S8: Sensitivity analyses of ETT-2 using alternative dispensing-gap definitions (30, 60, and 180 days) to define treatment discontinuation

Supplementary figures

Supplementary Figure 1

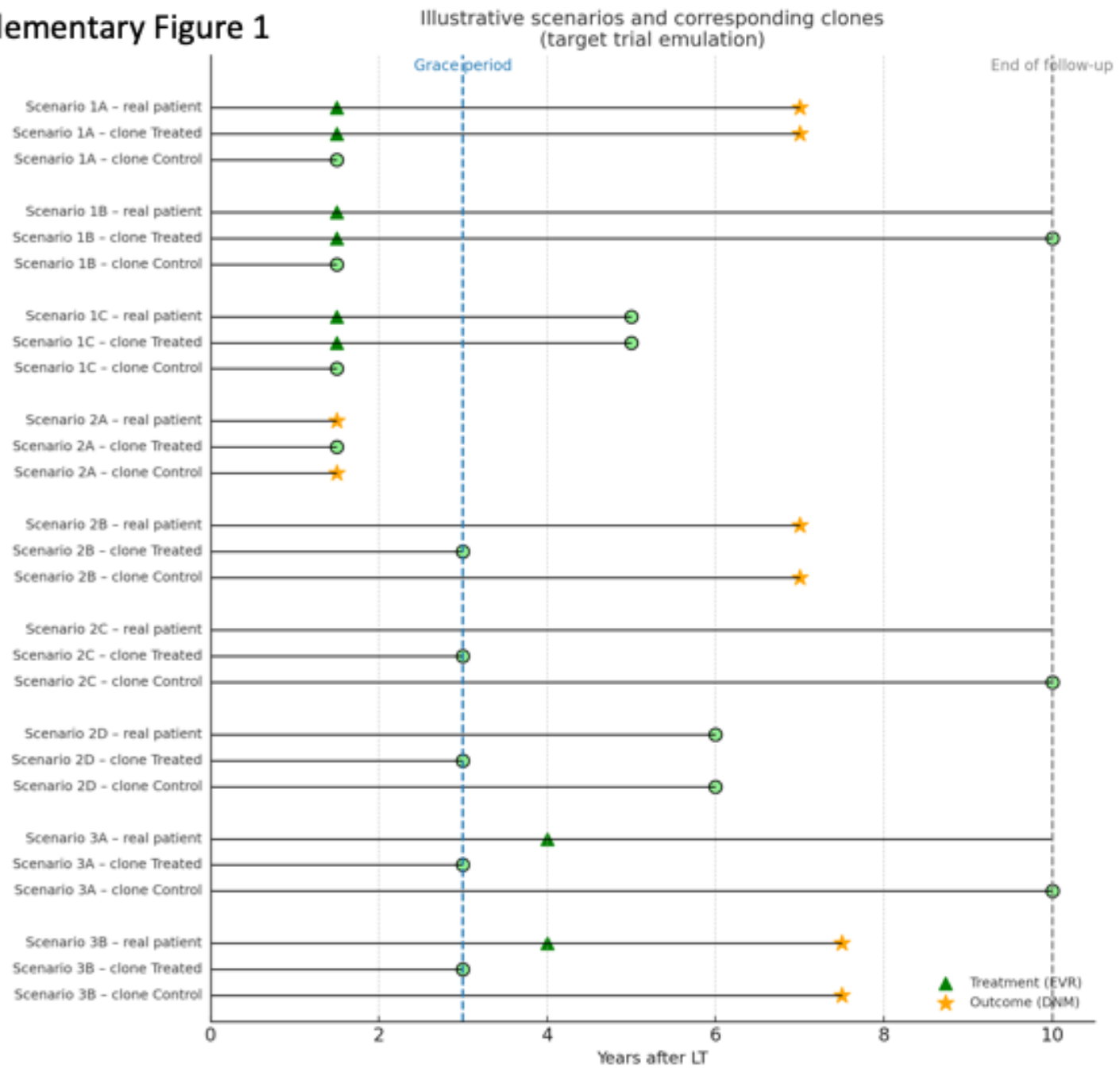

Hypothetical analysis dataset
for the target trial

Scenario	Arm	δ (event)	T* (years)
1A	Treated	1	7.0
1A	Control	0	1.5
1B	Treated	0	10.0
1B	Control	0	1.5
1C	Treated	0	5.0
1C	Control	0	1.5
2A	Treated	0	1.5
2A	Control	1	1.5
2B	Treated	0	3.0
2B	Control	1	7.0
2C	Treated	0	3.0
2C	Control	0	10.0
2D	Treated	0	3.0
2D	Control	0	6.0
3A	Treated	0	3.0
3A	Control	0	10.0
3B	Treated	0	3.0
3B	Control	1	7.5

Supplementary Figure 2

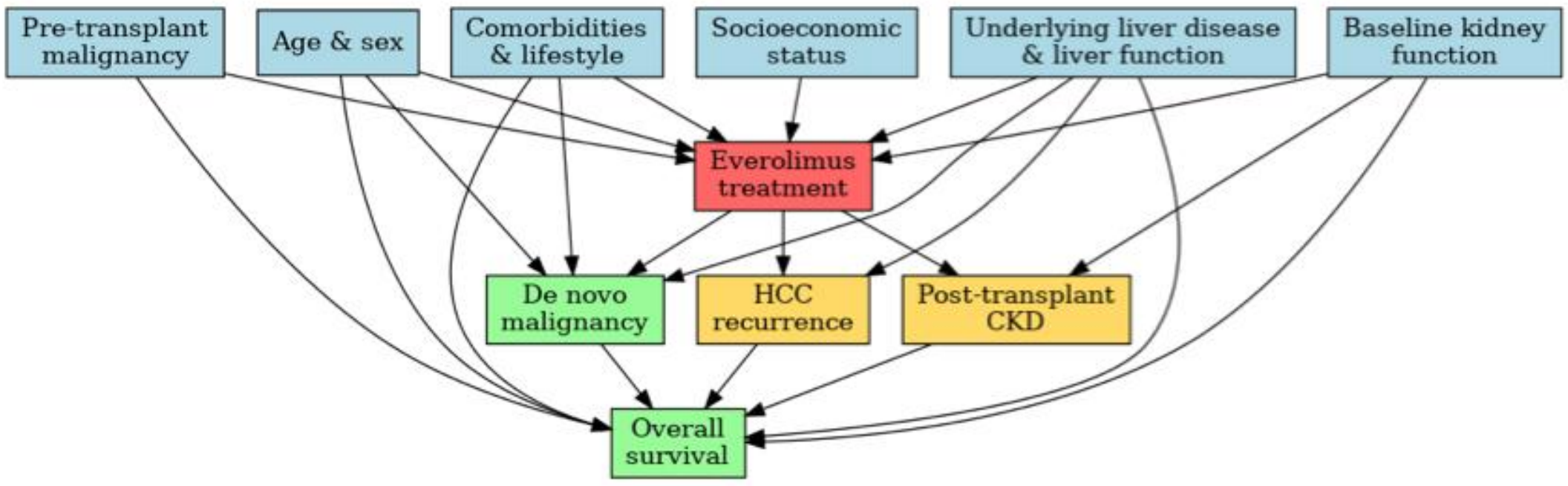

Supplementary Figure 3

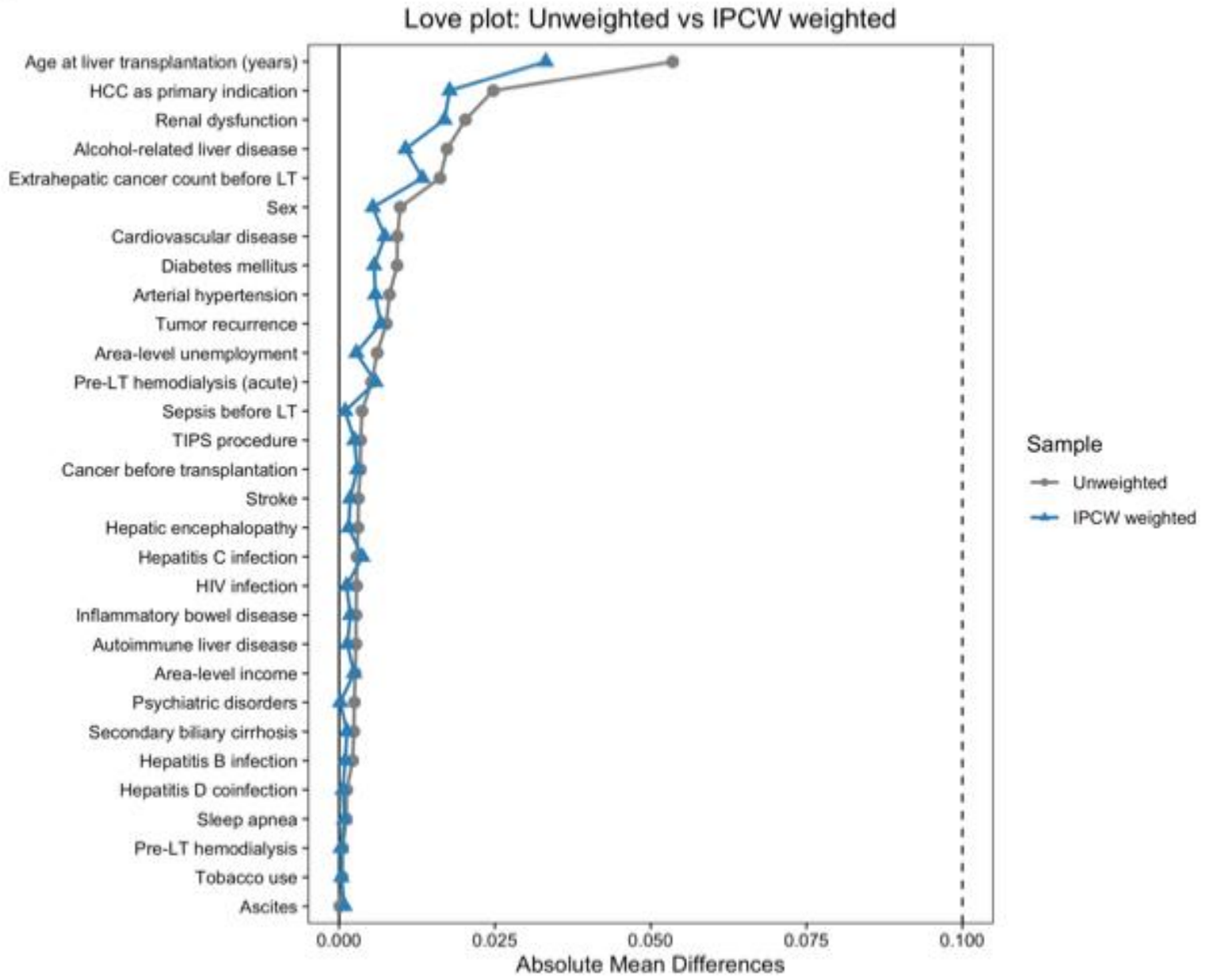

Supplementary Figure 4

Site-Specific Cumulative Incidence of *De Novo* Malignancies

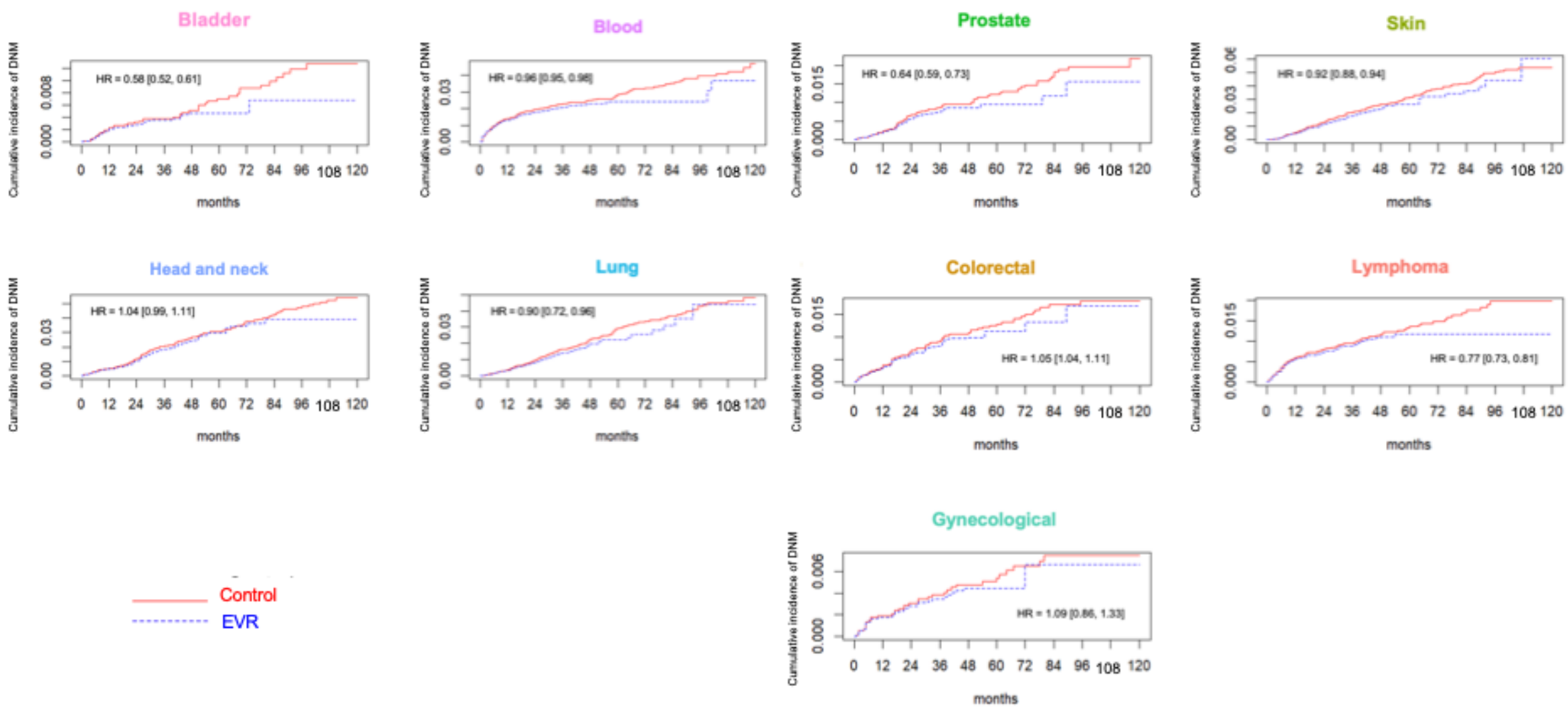

Supplementary Figure 5

Cumulative incidence of de novo malignancy after everolimus initiation using different grace periods (lag time = 0).

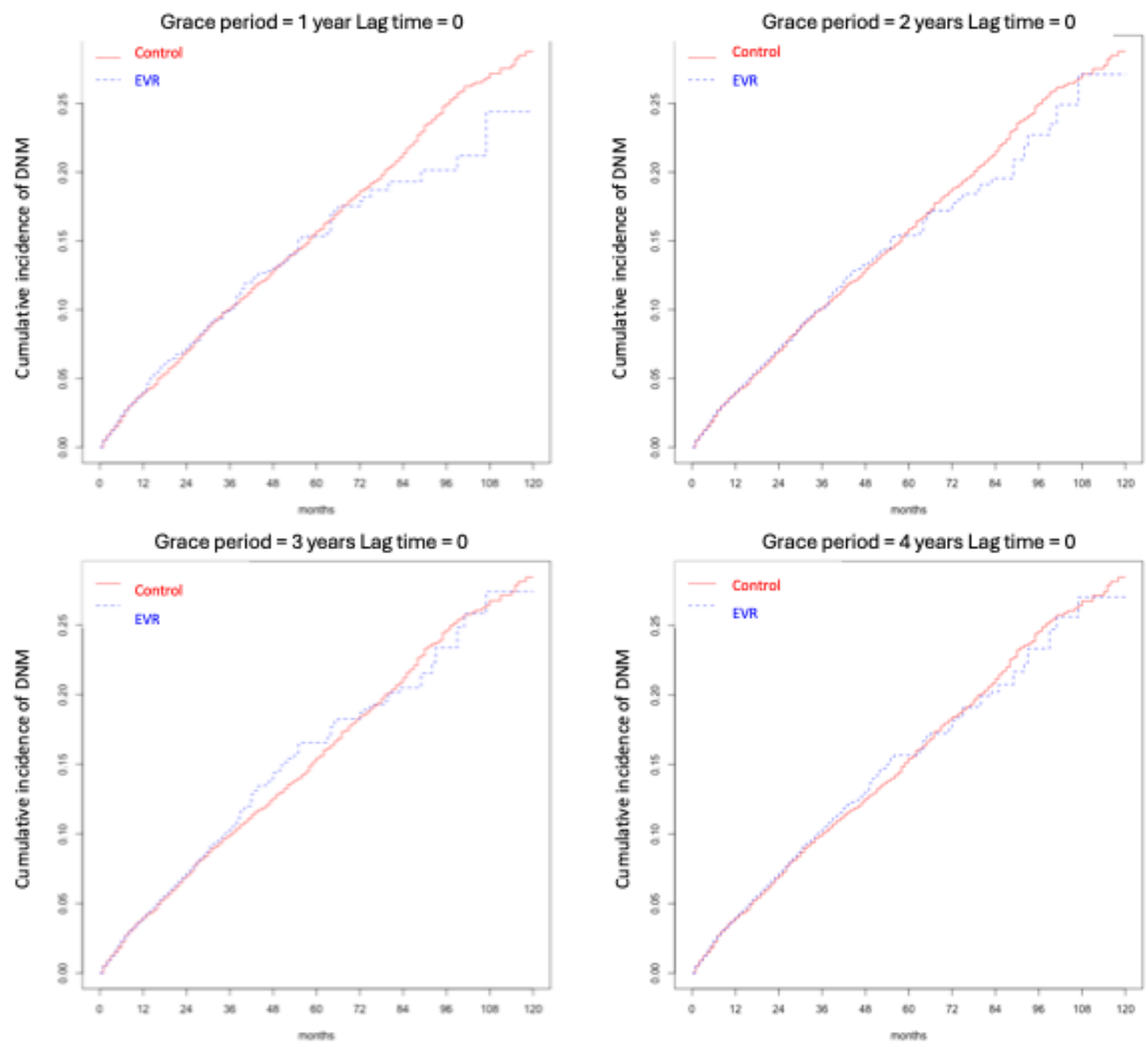

Supplementary Figure 5

Cumulative incidence of de novo malignancy after everolimus discontinuation using different grace periods (lag time = 0).

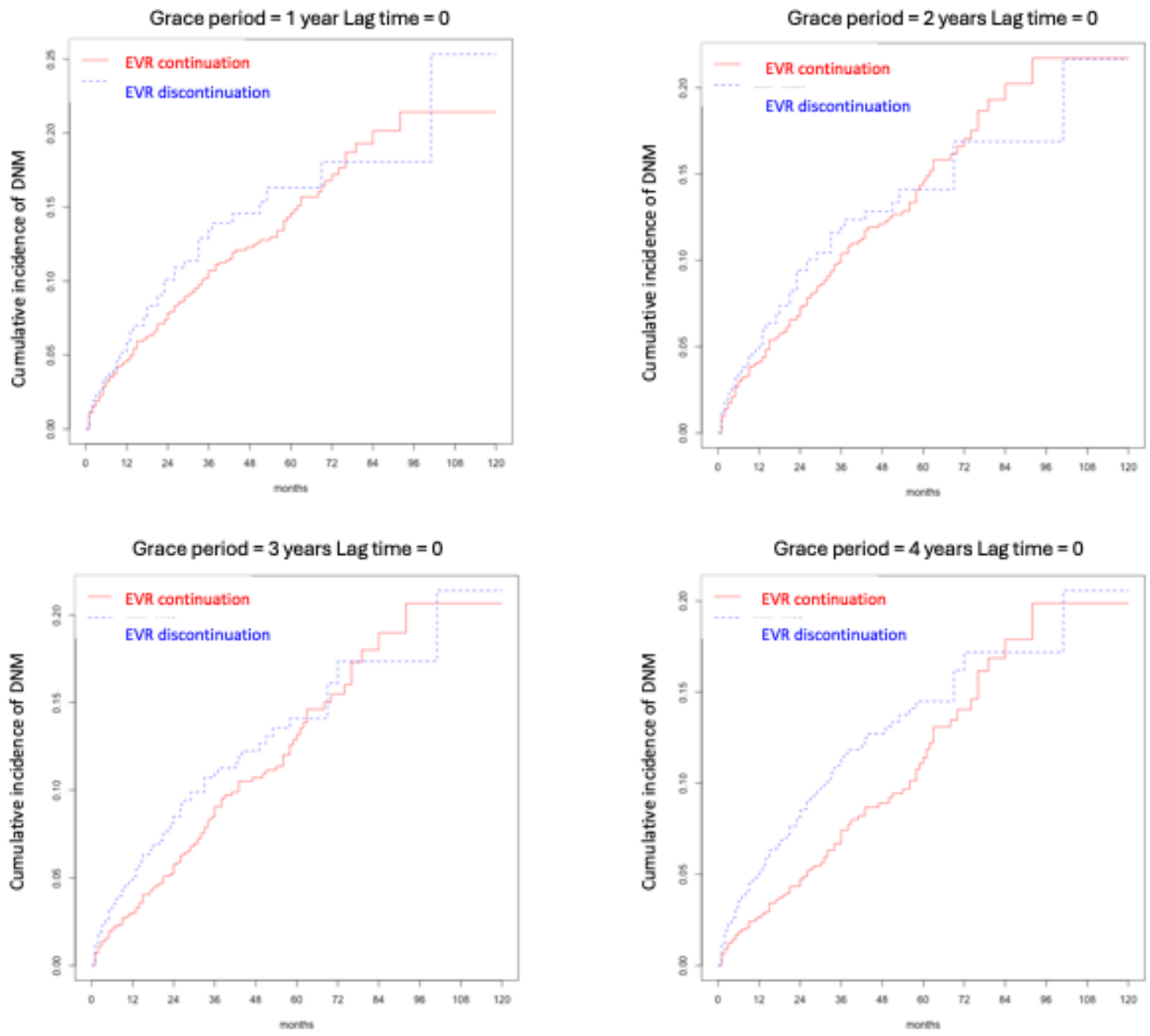

Supplementary Figure 6

Cumulative incidence of de novo malignancy after everolimus initiation for patients transplanted before end of 2015 with 3-year grace period and 2-year lag time

DNM occurrence
HR: 0.85 (0.81-0.88)

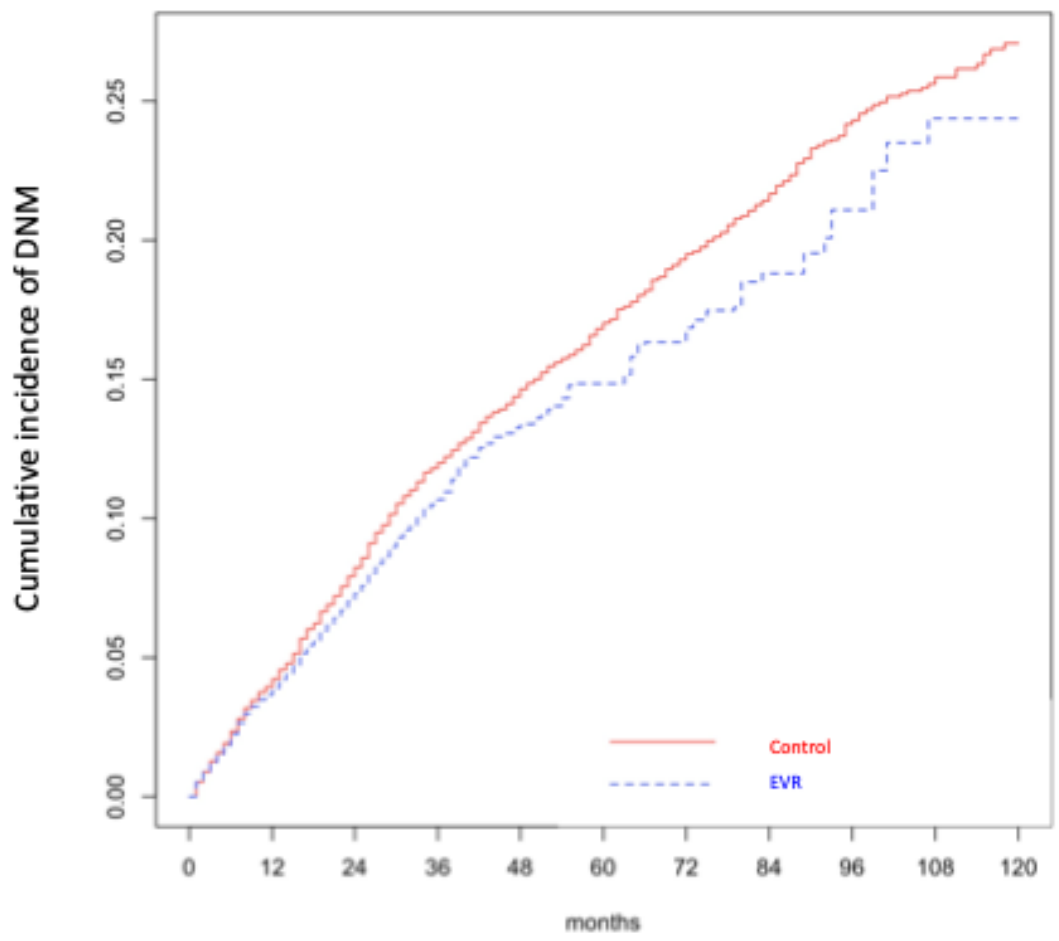

Overall survival
HR: 1.04 (0.92-1.10)

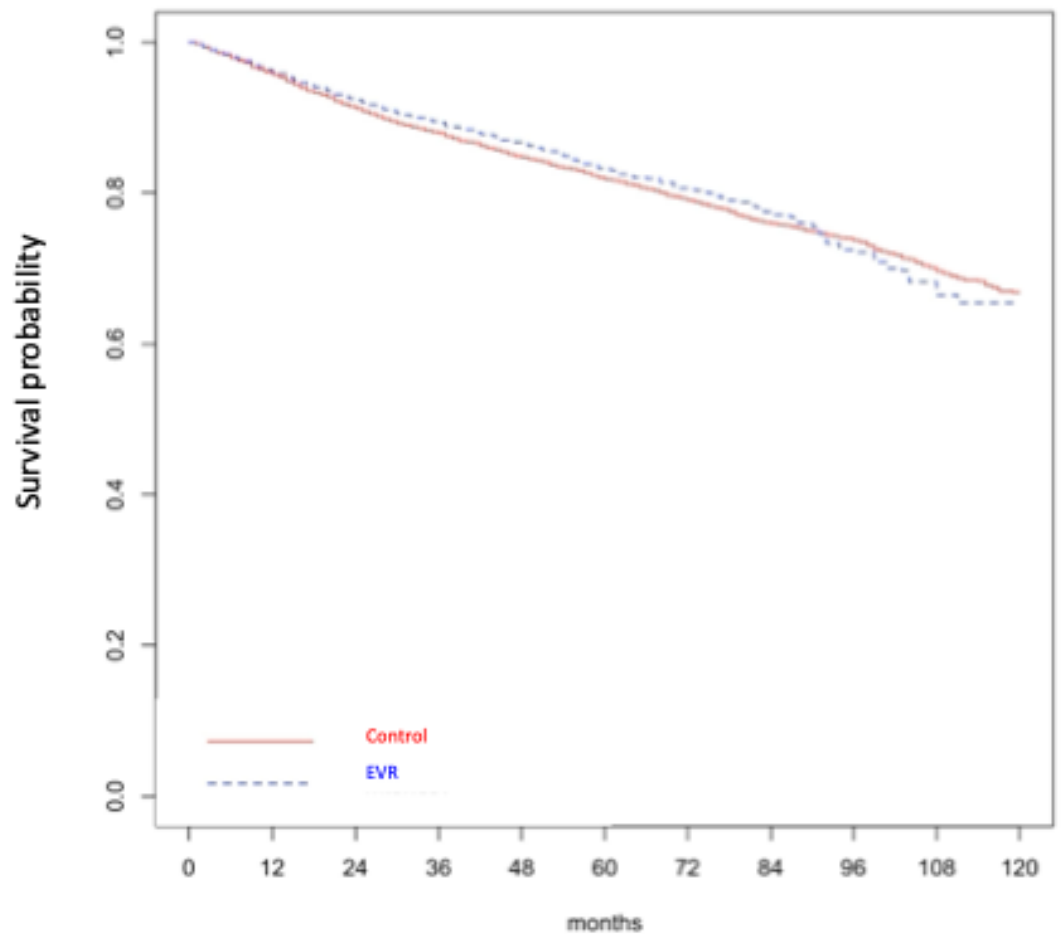

Supplementary Figure 7

Cumulative incidence of de novo malignancy after everolimus initiation for patients transplanted for HCC with 3-year grace period and 2-year lag time

DNM occurrence
HR: 0.87 (0.72-0.90)

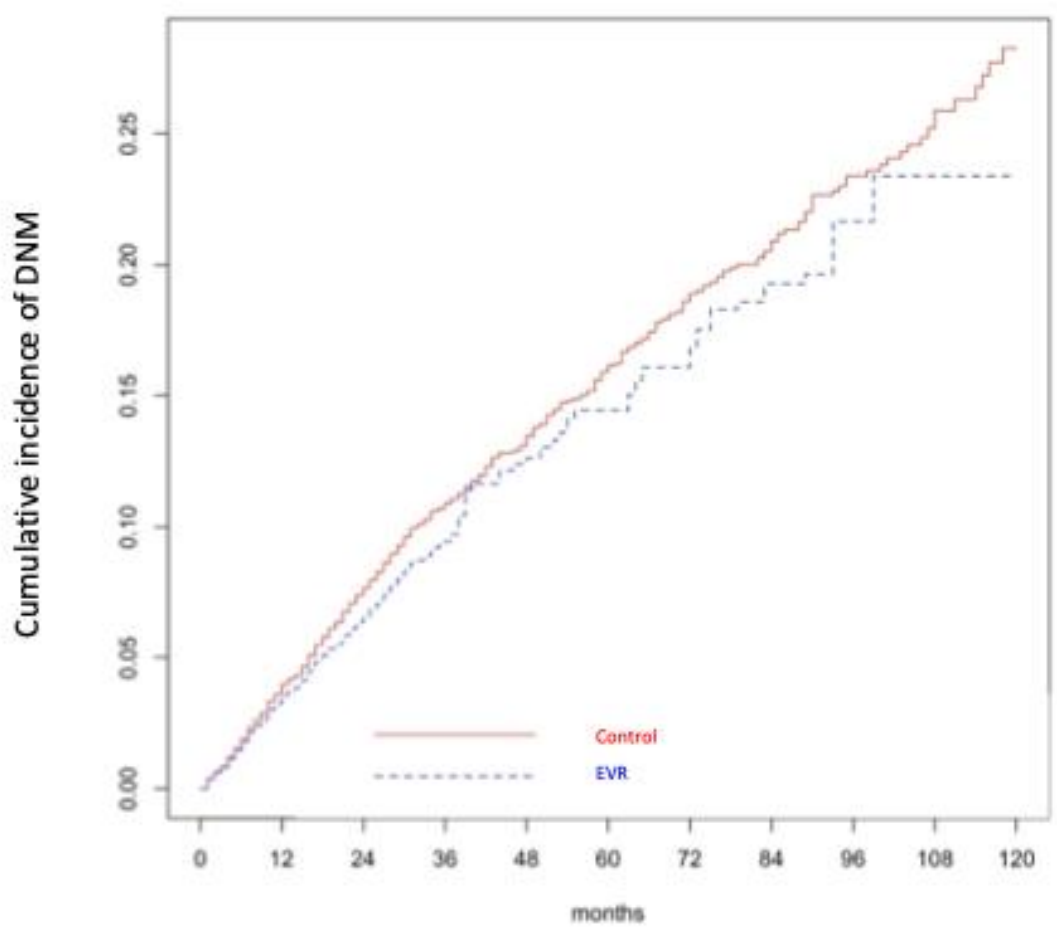

Overall survival
HR: 0.99 (0.98-1.01)

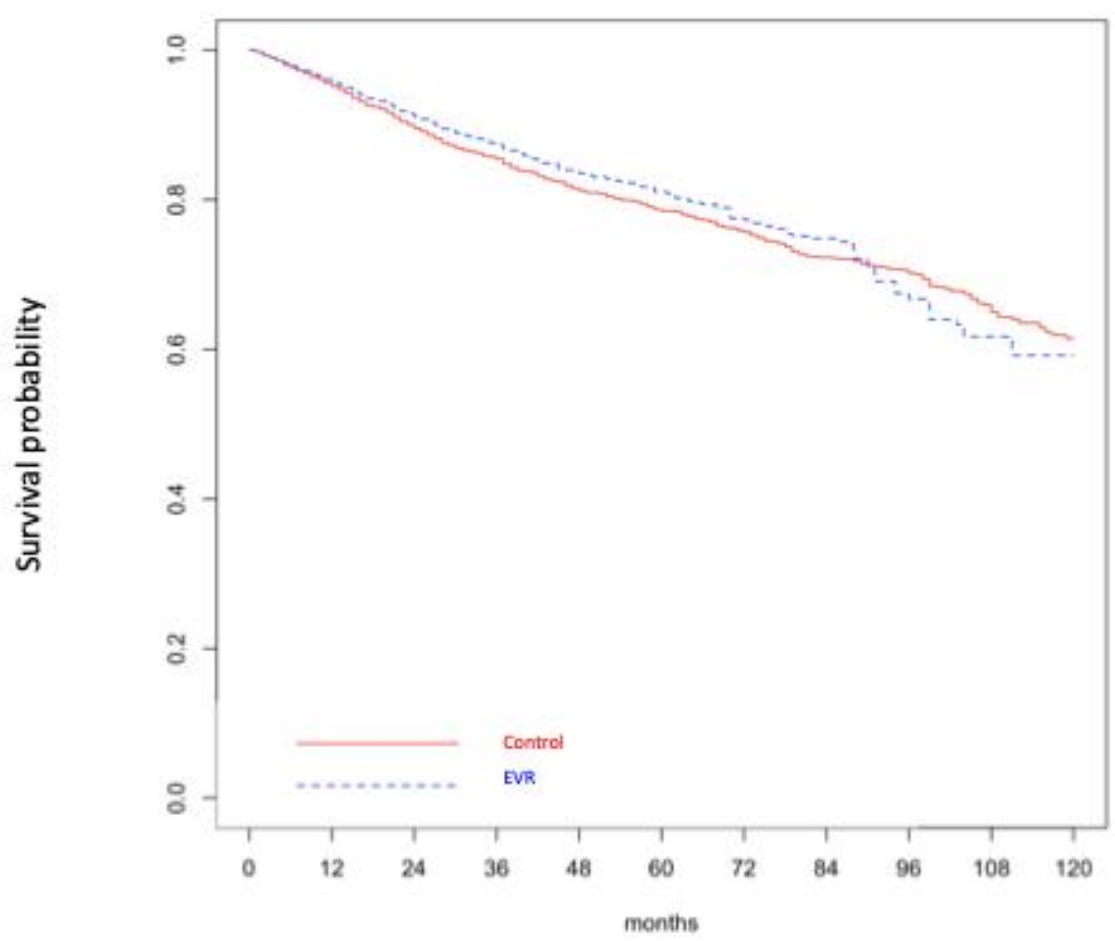

Supplementary tables

Table S1: Algorithm used to identify liver transplant recipients from the French national healthcare database.

Liver transplantation	
**CIM codes**	
Z944	Liver transplant [Orthotopic liver transplant]
(T864)	*(Liver transplant failure and rejection)*
**CCMP codes**	
HLEA002	Reduced liver transplantation
HLEA001	Total liver transplantation
HLQX008	Histopathological examination for oncological purposes of total hepatectomy specimens
**ATC codes**	
Reimbursement at least 3 months after hospital discharge after LT of one of the following:<br>L04AD02: Tacrolimus<br>L04AD01: Cyclosporine<br>L04AA06: Mycophenolic acid<br>L04AA18: Everolimus<br>And/or in association with one of the following:<br>L04AH01: Sirolimus<br>L04AX01: Azathioprine<br>H02AB06: Prednisolone<br>H02AB07: Prednisone	

Table S2: List of ICD-10 codes used to define cancer diagnoses for DNM classification.

ICD-10 Code	ICD label	Number of patients
**C22**	Hepatocellular carcinoma	3730
**C78**	Secondary malignant tumor of the lung	256
**C78**	Secondary malignant tumor of the liver and intrahepatic bile ducts	252
**C79**	Secondary malignant tumor of bone and bone marrow	240
**C34**	Malignant bronchus or lung tumor, unspecified	158
**C78**	Secondary malignant tumor of the retroperitoneum and peritoneum	156
**C44**	Malignant tumor of the skin of the face, other and unspecified parts	153
**C61**	Malignant prostate tumor	131
**C34**	Malignant tumor of the upper lobe, bronchi or lung	109
**C77**	Secondary and unspecified malignant tumor of the lymph nodes of the head,	92
**D46**	Refractory anemia, unspecified	84
**D47**	Monoclonal gammopathy of undetermined significance [GMSI]	82
**C77**	Secondary and unspecified malignant tumor of the intra-abdominal lymph nodes	76
**C78**	Secondary malignant tumor of the pleura	68
**C83**	Diffuse large B cell lymphoma	67
**C18**	Malignant colon tumor, unspecified	66
**C64**	Malignant tumor of the kidney, except the renal pelvis	64
**C67**	Malignant tumor of the bladder, unspecified	63
**C85**	Non-Hodgkin lymphoma, unspecified	55
**C34**	Malignant tumor of the lower lobe, bronchi or lung	51
**C32**	Malignant tumor of the larynx, unspecified	48
**C12**	Malignant tumor of the pyriform sinus	45
**D46**	Myelodysplastic syndrome, unspecified	45
**C20**	Malignant tumor of the rectum	44
**C15**	Malignant tumor of the esophagus, unspecified	43
**C50**	Malignant breast tumor, unspecified	43
**C01**	Malignant tumor of the base of the tongue	39
**C79**	Secondary malignant tumor of the brain and cerebral meninges	33
**C44**	Malignant tumor of the skin of the ear and external auditory canal	31
**C18**	Malignant tumor of the ascending colon	31
**C77**	Secondary and unspecified malignant tumor of the intrathoracic lymph nodes	27
**C10**	Malignant tumor of the oropharynx, unspecified	26
**C25**	Malignant tumor of the pancreas, unspecified	25
**C44**	Malignant tumor of the skin of the scalp and neck	24

**C44**	Malignant tumor of the skin of the upper limb, including the shoulder	24
**C79**	Secondary malignant tumor of other specific sites	24
**C02**	Malignant tumor of the tongue, unspecified	23
**C18**	Malignant tumor of the sigmoid colon	23
**C16**	Malignant stomach tumor, unspecified	22
**C85**	B-cell lymphomas, unspecified	22
**C32**	Malignant glottis tumor	22
**C32**	Malignant tumor of the supraglottic level	22
**C09**	Malignant tumor of the tonsil, unspecified	20
**D45**	Polycythemia vera	20
**C44**	Malignant skin tumor, unspecified	19
**C15**	Malignant tumor of the lower third of the esophagus	19
**C79**	Secondary malignant tumor of the adrenal gland	18
**C13**	Malignant tumor of the hypopharynx, unspecified	18
**C04**	Malignant tumor of the anterior floor of the mouth	18
**C44**	Malignant tumor of the skin of the trunk	16
**C25**	Malignant tumor of the pancreatic head	16
**C34**	Malignant tumor with contiguous locations of the bronchi and lung	16
**C10**	Malignant tumor of the lateral wall of the oropharynx	16
**C67**	Malignant tumor of the lateral wall of the bladder	16
**C26**	Malignant tumor of poorly defined sites of the digestive system	16
**C04**	Malignant tumor of the floor of the mouth, unspecified	15
**C02**	Malignant tumor of the point and lateral border of the tongue	15
**D47**	Myeloproliferative disease (chronic)	15
**C90**	Multiple myeloma	15
**C73**	Malignant thyroid tumor	15
**C15**	Malignant tumor of the middle third of the esophagus	14
**C83**	Small B cell lymphoma	13
**C83**	Lymphoma (diffuse) non-follicular, unspecified	13
**D46**	Other myelodysplastic syndromes	13
**C80**	Malignant tumor of unspecified primary site, as described	13
**C17**	Malignant tumor of the small intestine, unspecified	13
**C44**	Malignant tumor of the skin of the eyelid, including the canthus	12
**C10**	Malignant tumor with contiguous locations of the oropharynx	12
**C18**	Malignant tumor of the descending colon	12
**C80**	Malignant tumor of unspecified site	12
**C77**	Secondary, unspecified malignant tumor of a lymph node, unspecified	11
**C15**	Malignant tumor of the thoracic esophagus	11
**C32**	Malignant tumor with contiguous locations of the larynx	11
**C92**	Acute myeloblastic leukemia [AML]	11
**C78**	Secondary malignant tumor of the mediastinum	10
**C44**	Malignant tumor of the cutaneous surface of the lip	10

**D00**	Carcinoma in situ of the lip, oral cavity and pharynx	10
**C10**	Malignant tumor of the glossoepiglottic groove	10
**C18**	Malignant tumor of the cecum	10
**C50**	Malignant tumor of the supero-outer quadrant of the breast	10
**D47**	Essential (hemorrhagic) thrombocytopenia	10
**C79**	Secondary malignant tumor of the skin	9
**C44**	Malignant tumor of the skin of the lower limb, including the hip	9
**C83**	Other forms of diffuse non-Hodgkin lymphoma	9
**C34**	Malignant tumor of the main bronchus	9
**C15**	Malignant tumor of the cervical esophagus	9
**C09**	Malignant tumor with contiguous locations of the tonsil	9
**C07**	Malignant tumor of the parotid gland	9
**C04**	Malignant tumor of the lateral floor of the mouth	9
**C16**	Malignant tumor of the pyloric antrum	8
**C82**	Follicular lymphoma, unspecified	8
**C05**	Malignant tumor of the soft palate	8
**C19**	Malignant tumor of the rectosigmoid junction	8
**C18**	Malignant tumor of the transverse colon	8
**C18**	Malignant tumor of the left angle of the colon	8
**C71**	Malignant brain tumor, unspecified	8
**D46**	Refractory non-sideroblastic anemia, as specified	8
**D06**	Carcinoma in situ of the cervix, unspecified	7
**C53**	Malignant tumor of the cervix, unspecified	7
**C16**	Malignant tumor of the cardia	7
**C16**	Malignant tumor of the stomach body	7
**C25**	Malignant tumor of the endocrine pancreas	7
**C79**	Secondary malignant tumor of parts of the nervous system, other and unspecified	7
**C14**	Malignant tumor with contiguous locations of the lip, oral cavity and	7
**C21**	Malignant anal canal tumor	7
**C62**	Malignant tumor of a testicle, unspecified	6
**C46**	Kaposi sarcoma of the skin	6
**C38**	Malignant tumor of the pleura	6
**C56**	Malignant tumor of the ovary	6
**C85**	Other specific types of non-Hodgkin lymphoma	6
**C15**	Malignant tumor of the upper third of the esophagus	6
**C13**	Malignant tumor with contiguous locations of the hypopharynx	6
**C10**	Malignant tumor of the anterior surface of the epiglottis	6
**C09**	Malignant tumor of the tonsil pillar (anterior) (posterior)	6
**C06**	Malignant tumor of the mouth, unspecified	6
**C21**	Malignant tumor of the anus, unspecified	6
**D06**	Carcinoma in situ of the endocervix	5
**C53**	Malignant tumor of the endocervix	5
**C24**	Malignant tumor of the ampulla of Vater	5

**C78**	Secondary malignant tumor of the digestive organs, other and unspecified	5
**C83**	Burkitt lymphoma	5
**C81**	Hodgkin's lymphoma, unspecified	5
**C34**	Malignant tumor of the middle lobe, bronchi or lung	5
**C14**	Malignant tumor of the pharynx, unspecified	5
**C13**	Malignant tumor of the posterior wall of the hypopharynx	5
**C10**	Malignant tumor of the posterior wall of the oropharynx	5
**C05**	Malignant tumor of the palate, unspecified	5
**C04**	Malignant tumor with contiguous locations in the floor of the mouth	5
**C02**	Malignant tumor with contiguous locations of the tongue	5
**C18**	Malignant tumor of the right angle of the colon	5
**C91**	B-cell chronic lymphocytic leukemia	5
**C79**	Secondary malignant tumor of the bladder and urinary organs, other and not p	5
**C16**	Malignant tumor of the greater curvature of the stomach, unspecified	4
**C25**	Malignant tumor of the tail of the pancreas	4
**C25**	Malignant tumor of the pancreatic duct	4
**C84**	T-cell lymphomas, other and unspecified	4
**C41**	Malignant tumor of the bones of the skull and face	4
**C41**	Malignant tumor of the mandible	4
**C15**	Malignant tumor of the abdominal esophagus	4
**C15**	Malignant tumor with contiguous locations of the esophagus	4
**C13**	Malignant tumor of the aryepiglottic fold (hypopharyngeal side)	4
**C11**	Malignant tumor of the nasopharynx, unspecified	4
**C09**	Malignant tumor of the tonsillar fossa	4
**C02**	Malignant tumor of the dorsal surface of the tongue	4
**C02**	Malignant tumor of the anterior two-thirds of the tongue, unspecified	4
**C18**	Malignant tumor of the appendix	4
**C50**	Malignant tumor of the central part of the breast	4
**D46**	Refractory sideroblastic anemia	4
**D46**	Refractory anemia with excess blasts [AREB]	4
**C95**	Leukemia, unspecified	4
**C88**	Waldenstrom macroglobulinemia	4
**C67**	Malignant tumor with contiguous locations of the bladder	4
**C54**	Malignant endometrial tumor	3
**C62**	Malignant tumor of the descended testicle	3
**C16**	Malignant fundus tumor	3
**C84**	Mycosis fungoides	3
**C43**	Malignant melanoma of the face, other and unspecified parts	3
**C43**	Malignant melanoma of the lower limb, including the hip	3
**C43**	Melanoma (malignant) of the skin, unspecified	3

**C45**	Pleural mesothelioma	3
**C25**	Malignant tumor of the body of the pancreas	3
**C77**	Secondary and unspecified malignant tumor of the intrapelvic lymph nodes	3
**C88**	B-cell marginal zone lymphoma of mu-associated lymphoid tissues	3
**C82**	Follicular lymphoma, grade II	3
**C06**	Malignant tumor of the retromolar region	3
**C03**	Malignant tumor of the upper gum	3
**C03**	Malignant tumor of the lower gum	3
**C03**	Malignant tumor of the gum, unspecified	3
**C02**	Malignant tumor of a lingual tonsil	3
**C18**	Malignant tumor with contiguous locations of the colon	3
**C50**	Malignant tumor of the upper inner quadrant of the breast	3
**C50**	Malignant tumor with contiguous locations of the breast	3
**C71**	Malignant tumor of the brain, except lobes and ventricles	3
**D47**	Osteomyelofibrosis	3
**C95**	Acute unspecified cell leukemia	3
**C92**	Chronic myeloid leukemia [CML] ABL-BCR positive	3
**C67**	Malignant tumor of the dome of the bladder	3
**C74**	Malignant adrenal tumor, unspecified	3
**C66**	Malignant tumor of the ureter	3
**C49**	Malignant tumor of the connective tissue and other soft tissues of the upper limb	3
**C46**	Multiple organ Kaposi sarcoma	3
**C45**	Mesothelioma of the peritoneum	3
**C17**	Malignant tumor of the duodenum	3
**C44**	Malignant tumor with contiguous locations of the skin	2
**C25**	Malignant tumor of other parts of the pancreas	2
**C78**	Secondary malignant tumor of the respiratory organs, other and unspecified	2
**C78**	Secondary malignant tumor of the small intestine	2
**C77**	Secondary and unspecified malignant tumor of the inguinal lymph nodes	2
**C77**	Secondary and unspecified malignant tumor of the lymph nodes of the m sites	2
**D47**	Other specified tumors with unpredictable and unknown evolution of lymphoid tissues	2
**C84**	Peripheral T-cell lymphoma, not elsewhere classified	2
**C84**	Cutaneous T-cell lymphoma, unspecified	2
**C83**	Mantle cell lymphoma	2
**C82**	Follicular lymphoma, grade I	2
**C82**	Follicular lymphoma, grade III, unspecified	2
**B21**	HIV disease causes other malignant tumors of lymphoid tissues, hematologic	2

**C00**	Malignant tumor of the lower lip, (free edge)	2
**C79**	Secondary malignant tumor of the kidney and renal pelvis	2
**H60**	Malignant external otitis	2
**C76**	Malignant tumor of ill-defined location of the head, face and neck	2
**C32**	Malignant tumor of the subglottic level	2
**C11**	Malignant tumor of the upper wall of the nasopharynx	2
**C11**	Malignant tumor of the posterior wall of the nasopharynx	2
**C11**	Malignant tumor with contiguous locations of the nasopharynx	2
**C06**	Malignant tumor of the cheek mucosa	2
**C05**	Malignant tumor of the uvula	2
**C78**	Secondary malignant tumor of the large intestine and rectum	2
**C21**	Malignant tumor with contiguous locations of the rectum, anus and anal canal	2
**C71**	Malignant frontal lobe tumor	2
**C70**	Malignant tumor of the cerebral meninges	2
**D47**	Chronic eosinophilic leukemia [hypereosinophilic syndrome]	2
**D46**	Refractory anemia with dysplasia of multiple cell lines	2
**C90**	Extramedullary plasmacytoma	2
**C67**	Malignant trigone tumor of the bladder	2
**C67**	Malignant tumor of the anterior wall of the bladder	2
**C76**	Malignant tumor of ill-defined location of the pelvis	2
**C67**	Malignant tumor of the ureteral orifice	2
**C49**	Malignant tumor of the connective tissue and other soft tissues of the head, f	2
**C49**	Malignant tumor of the connective tissue and other soft tissues of the lower limb	2
**C49**	Malignant tumor with contiguous locations of connective tissue and other tissues	2
**C48**	Malignant tumor with contiguous locations of the retroperitoneum and peritoneum	2
**C46**	Soft tissue Kaposi sarcoma	2
**C46**	Kaposi's sarcoma of lymph nodes	2
**C46**	Kaposi's sarcoma, unspecified	2
**C41**	Malignant tumor of the spine	2
**C41**	Malignant tumor of the ribs, sternum and clavicle	2
**C41**	Malignant tumor of the bones of the pelvis, sacrum and coccyx	2
**C17**	Malignant tumor of the jejunum	2
**C17**	Malignant tumor of the ileum	2
**B21**	HIV disease causing a malignant tumor, unspecified	2
**D06**	Carcinoma in situ of other parts of the cervix	1
**C57**	Malignant tumor of the adnexa of the uterus, unspecified	1
**C54**	Malignant tumor with contiguous locations of the body of the uterus	1
**C53**	Malignant tumor of the ectocervix	1

**C53**	Malignant tumor with contiguous locations of the cervix of the uterus	1
**C63**	Malignant spermatic cord tumor	1
**C62**	Malignant tumor of the ectopic testicle	1
**D00**	Carcinoma in situ of the stomach	1
**C16**	Malignant tumor of the lesser curvature of the stomach, unspecified	1
**C16**	Malignant tumor with contiguous locations of the stomach	1
**D04**	Carcinoma in situ of the skin of the trunk	1
**C43**	Malignant melanoma of the ear and external auditory canal	1
**C43**	Malignant melanoma of the scalp and neck	1
**C43**	Malignant melanoma of the trunk	1
**C25**	Malignant tumor with contiguous locations of the pancreas	1
**C77**	Secondary, unspecified malignant tumor of the lymph nodes of the armpit	1
**C91**	Adult T-cell lymphoma/leukemia (HTLV-1 associated)	1
**C86**	T-cell (intestinal) lymphoma, enteropathic type	1
**C86**	Angioimmunoblastic T-cell lymphoma	1
**C85**	Mediastinal (thymic) large B-cell lymphoma	1
**C84**	ALK-positive anaplastic large cell lymphoma	1
**C83**	Immunoblastic lymphoma (diffuse)	1
**C83**	Lymphoblastic lymphoma (diffuse)	1
**C83**	Undifferentiated lymphoma (diffuse)	1
**C82**	Follicular lymphoma, grade IIIa	1
**C82**	Follicular lymphoma, grade IIIb	1
**C82**	Other forms of follicular lymphoma	1
**C81**	Hodgkin's lymphoma, predominantly lymphocytic, nodular form	1
**C81**	Hodgkin's lymphoma (classic), rich in lymphocytes	1
**B21**	HIV disease causes Burkitt lymphoma	1
**B21**	HIV disease causes other non-Hodgkin's lymphomas	1
**C00**	Malignant tumor of the corner of the lips	1
**C38**	Malignant tumor of the heart	1
**C69**	Malignant tumor of the orbit	1
**C69**	Malignant tumor with contiguous locations of the eye and its adnexa	1
**C32**	Malignant tumor of the laryngeal cartilage	1
**C31**	Malignant tumor with contiguous locations in the sinuses of the face	1
**C31**	Malignant tumor of the facial sinuses, unspecified	1
**C11**	Malignant tumor of the lateral wall of the nasopharynx	1
**C08**	Malignant tumor of the submandibular gland	1
**C06**	Malignant tumor of the vestibule of the mouth	1
**C06**	Malignant tumor with contiguous locations of the mouth, other parts and not pre	1
**C05**	Malignant tumor of the palatal vault	1

**C05**	Malignant tumor with contiguous locations on the palate	1
**D01**	Carcinoma in situ of the rectosigmoid junction	1
**D05**	Other carcinomas in situ of the breast	1
**C50**	Malignant tumor of the lower outer quadrant of the breast	1
**C50**	Malignant tumor of the axillary extension of the breast	1
**C50**	Malignant tumor of the nipple and areola	1
**C71**	Malignant tumor of the occipital lobe	1
**C71**	Malignant tumor with contiguous locations of the brain	1
**C70**	Malignant tumor of the meninges, unspecified	1
**C94**	Acute erythroid leukemia	1
**C94**	Acute panmyelosis with myelofibrosis	1
**C94**	Myelodysplastic and myeloproliferative disease, not elsewhere classified	1
**C93**	Acute monoblastic/monocytic leukemia	1
**C92**	Other myeloid leukemias	1
**C92**	Acute myeloid leukemia with dysplasia of several cell lines	1
**C92**	Myeloid leukemia, unspecified	1
**C91**	Hairy cell leukemia	1
**C91**	Other lymphoid leukemias	1
**C91**	Lymphocytic leukemia, unspecified	1
**C90**	Solitary plasmacytoma	1
**C88**	Other malignant immunoproliferative diseases	1
**C88**	Immunoproliferative (malignant) disease, unspecified	1
**C86**	Primary cutaneous lymphoproliferative syndrome with CD30-positive T cells	1
**C68**	Malignant tumor of the urethra	1
**C67**	Malignant tumor of the posterior wall of the bladder	1
**C96**	Multifocal and multisystem Langerhans cell histiocytosis (disseminated	1
**C96**	Multifocal and unisystemic Langerhans cell histiocytosis	1
**C96**	Unifocal Langerhans cell histiocytosis	1
**C96**	Histiocytic sarcoma	1
**C76**	Malignant tumor of ill-defined location of the thorax	1
**C76**	Malignant tumor of ill-defined location of the abdomen	1
**C76**	Malignant tumor of poorly defined site of a lower limb	1
**C74**	Malignant tumor of the adrenal medulla	1
**C72**	Malignant tumor with contiguous locations of the brain and other parts of the brain	1
**C72**	Malignant tumor of the (central) nervous system, unspecified	1
**C69**	Malignant choroidal tumor	1
**C69**	Malignant tumor of the lacrimal gland and lacrimal duct	1
**C68**	Malignant tumor of a urinary organ, unspecified	1
**C60**	Malignant tumor of the foreskin	1
**C60**	Malignant glans tumor	1
**C60**	Malignant tumor of the penis, unspecified	1

**C57**	Malignant tumor with contiguous locations of the female genital organs	1
**C57**	Malignant tumor of a female genital organ, unspecified	1
**C52**	Malignant tumor of the vagina	1
**C51**	Malignant tumor of the vulva, unspecified	1
**C49**	Malignant tumor of the connective tissue and other soft tissues of the trunk, unspecified	1
**C49**	Malignant tumor of connective tissue and other soft tissues, unspecified	1
**C48**	Malignant tumor of the retroperitoneum	1
**C48**	Malignant peritoneal tumor, unspecified	1
**C46**	Pulmonary Kaposi sarcoma	1
**C46**	Kaposi's sarcoma of other locations	1
**C45**	Mesothelioma, unspecified	1
**C41**	Malignant tumor with contiguous locations of bones and articular cartilage	1
**C40**	Malignant tumor of the scapula and long bones of the upper limb	1
**C40**	Malignant tumor of the short bones of the upper limb	1
**C40**	Malignant tumor of the bones and articular cartilage of a limb, unspecified	1
**C39**	Malignant tumor of the upper respiratory tract, part unspecified	1
**C39**	Malignant tumor with contiguous locations of the respiratory and intrathorax organs	1
**C38**	Malignant tumor of the posterior mediastinum	1
**C37**	Malignant tumor of the thymus	1
**C26**	Malignant tumor of the spleen	1
**C17**	Malignant tumor with contiguous locations of the small intestine	1
**B21**	HIV disease causing Kaposi's sarcoma	1

Table S3: CCMP procedure codes used to confirm the diagnosis of DNM

Code	Procedure	Number of patients
**HHFE006**	Rectocolic mucosectomy session, by endoscopy	441
**HHFE004**	Excision of a polyp of more than 1cm in diameter or of 4 or more polyps of the colon	440
**ZZMP015**	Creation of a personalized [individualized] restraint system for radiothesis	245
**ZZML002**	Control of the dose of external irradiation received [In vivo dosimetry]	222
**FCQX006**	Pathological examination with an oncological purpose of a group's surgical specimen	207
**HHQX004**	Pathological examination for oncological purposes of endoscopic surgical specimen	201
**ZZMK018**	Preparation for external irradiation with identification by CT scan, dosimetry	170
**ZZNL063**	External irradiation session by linear accelerator of equal or greater power	170
**YYYY128**	Supplement for setting up a multi-leaf collimator	157
**HLQX005**	Pathological examination for oncological purposes of specimens from several hepatectomies	156
**FCQX010**	Pathological examination for oncological purposes of 2 or 3 excised specimen	146
**PDFA001**	Excision of fascial and/or subfascial lesion of soft tissues	133
**ZZML001**	Control of the centering of external irradiation fields	132
**ZZML003**	Control of the centering of external irradiation fields at the center	122
**QZFA036**	Excision of superficial lesion of the skin by excision of a cutaneous area	118
**QZQX004**	Pathological examination for oncological purposes of a specimen of skin excision	111
**ZZNL051**	External irradiation session by dedicated machine producing photons	91
**ZZNL054**	External irradiation session by linear accelerator with intensity modulation	89
**ZZMP016**	Preparation for external irradiation in stereotaxic conditions without synchronization	87
**ZZNL060**	External irradiation session in stereotaxic conditions by dedicated professional machine	87
**HFQX005**	Pathological examination for oncological purposes of endoscopic surgical specimen	85
**JDQE001**	Urethrovesical fibroscopy	84
**ZZNL065**	External irradiation session by linear accelerator of equal or greater power	84

**QZFA004**	Excision of superficial lesion of the skin	74
**HLNC003**	Destruction of hepatic tumor with radiofrequency current	67
**YYYY026**	Supplement for mediastinal lymph node dissection during pneumonectomy	65
**HGFA005**	Single segmental resection of the small intestine for obstruction, by laparotomy	62
**JGHJ001**	Prostate biopsy, transrectally with ultrasound guidance	60
**FCQX012**	Pathological examination for oncological purposes of surgical specimens weighing 4 to 6 g	59
**JDFE001**	Resection of 4 or more bladder tumors, by endoscopy	58
**GFFA013**	Pulmonary lobectomy, by thoracotomy	53
**HLNA007**	Destruction of hepatic tumor with radiofrequency current, by laparotomy	50
**QZQX032**	Pathological examination for oncological purposes of several surgical specimens	50
**QZQX005**	Pathological examination for oncological purposes of skin and/or excision specimen	49
**17 FCFA**	Lymphonodal dissection of the hilum of the lung and the mediastinum, during an intervention	48
**JDFE002**	Resection of 1 to 3 bladder tumors, by endoscopy	45
**GFFC002**	Non-anatomic partial excision of the lung, by thoracoscopy	44
**ZZNL059**	External irradiation session in stereotaxic conditions by dedicated professional machine	44
**GFQX004**	Pathological examination for oncological purposes of a excised specimen from lobectomy	42
**HHFA009**	Right colectomy with restoration of continuity, by laparotomy	41
**HGQX005**	Pathological examination for oncological purposes of endoscopic surgical specimen	40
**HHFA026**	Right colectomy without restoration of continuity, by laparotomy	37
**JKFA031**	Conization of the cervix	37
**LBFA023**	Excision of lesion of the maxillary bone and/or body of the mandible	37
**QZEA031**	Total skin autograft on a surface location less than 10 cm_	37
**YYYY166**	Supplement for quality ballistic controls in p-imaging radiotherapy	37
**GFQX006**	Pathological examination for oncological purposes of pulmonary excision specimen	36
**HMQX004**	Pathological examination for oncological purposes of duct excision specimen	36
**HHQX008**	Pathological examination for oncological purposes of endoscopic surgical specimen	35
**HPQX005**	Pathological examination for oncological purposes of a tumor excision specimen	35

**HHQX006**	Pathological examination for oncological purposes of partial colectomy specimen	34
**QZFA007**	Excision of subcutaneous suprafascial lesion from 3 cm to 10 cm long axis	34
**HHFA021**	Total colectomy with preservation of the rectum, without restoration of continuity	33
**KCFA005**	Total thyroidectomy, by cervicotomy	32
**ZZNL050**	External irradiation session by linear accelerator with intensity modulation	32
**GFFA009**	Pulmonary lobectomy, by thoracotomy with preparation by thoracoscopy	29
**JGFC001**	Total vesiculoprostatectomy, by laroscopy	29
**HAQX017**	Pathological examination for oncological purposes of glossectomy specimen	27
**16 FCFA**	Unilateral partial cervical lymph node dissection, by cervicotomy	27
**GDFE004**	Excision of lesion or peeling of the mucosa of the glottic plane, by laryngoscopy	27
**FCQX011**	Pathological examination for oncological purposes of surgical specimens from 7 groups	25
**GFFA017**	Single non-anatomic partial excision of the lung, by thoracotomy	24
**JAQX005**	Pathological examination for oncological purposes of partial nephrectomy specimen	23
**QZFA002**	Excision of a subcutaneous suprafascial lesion of less than 3 cm in long axis	23
**JGFA006**	Total vesiculoprostatectomy, by laparotomy	22
**LAFA021**	Anterior ethmoidectomy, by endoscopy	22
**HNFA007**	Cephalic duodenopancreatectomy, by laparotomy	21
**HPFA004**	Resection of the greater omentum, by laparotomy	21
**GFFA029**	Single or multiple lung segmentectomy, by thoracotomy	20
**JKQX007**	Pathological examination for oncological purposes of cervical conization specimen	20
**JGQX004**	Pathological examination for oncological purposes of a vesiculoprostatectomy specimen	19
**JAFC005**	Partial nephrectomy, by laposcopy or retroperitoneoscopy	19
**LFFA001**	Bilateral total lumbar or lumbosacral laminarthrectomy, via posterior approach	19
**ZZMK020**	Resumption of preparation for external irradiation with identification by CT scan	19
**HKFA008**	Destruction and/or excision of non-tumorous superficial lesion of the anus	18
**KEFA002**	Partial or total adrenalectomy, by direct approach	18
**EPPP002**	Clearing obstruction of implantable site or tunneled central venous line by	17

**HGQX008**	Pathological examination for oncological purposes of segmental excision specimen	17
**KEQX004**	Pathological examination for oncological purposes of adrenalectomy specimen	16
**HEQX005**	Pathological examination for oncological purposes of endoscopic surgical specimen	16
**YYYY047**	Irradiation of less than 300 cm_ in fixed fields by accelerator by fraction of	16
**HHFA014**	Left colectomy without release of the left colic angle, without restoration	15
**HHFA006**	Left colectomy with release of the left colic angle, with restoration	15
**JAFA008**	Partial nephrectomy with dissection of the vascular pedicle, by lobectomy	15
**HHFA018**	Transverse colectomy, by laparotomy	14
**ZZNL052**	External irradiation session in stereotaxic conditions by machine producing	14
**GDFE003**	Laryngeal cordectomy, by direct laryngoscopy with laser	13
**HDFA002**	Lateral oropharyngectomy with tonsillectomy, via intraoral approach	13
**GFFA021**	Multiple non-anatomic partial excisions of the lung, by thoracotomy	13
**FAFA014**	Tonsillectomy by dissection	13
**GAFA005**	Transfixing excision of lesion of the skin of the nose and/or the nasal mucosa	13
**HFFA006**	Partial lower gastrectomy with gastrojejunal anastomosis, by laparotomy	12
**HAFA023**	Partial glossectomy of mobile tongue, via intraoral approach	12
**JHFA005**	Orchiectomy, via inguinal approach	12
**YYYY152**	Irradiation of less than 300 cm_ in fixed fields by accelerator by fraction of	12
**HFQX004**	Pathological examination for oncological purposes of gastrectomy specimen	11
**HAQX005**	Pathological examination for oncological purposes of excised specimen of lip	11
**HAQX004**	Pathological examination for oncological purposes of pelvimandibulectomy piece	11
**HCQX004**	Pathological examination for oncological purposes of gland excision specimen	11
**JGQX005**	Pathological examination for oncological purposes of a specimen of vesiculo-prostato-cystectomy	11
**JAQX004**	Pathological examination for oncological purposes of total nephrectomy specimen	11
**QEQX004**	Pathological examination for oncological purposes of a mastectomy specimen	11

**JKFA026**	Total hysterectomy, vaginal approach	11
**AZQX005**	Pathological examination for oncological purposes of a tumor excision specimen	11
**GGQX004**	Pathological examination for oncological purposes of a tumor excision specimen	11
**HAFA006**	Mobile tongue pelviglossectomy, via intraoral approach	11
**PAQX004**	Pathological examination for oncological purposes of a tumor excision specimen	11
**YYYY049**	Irradiation of less than 300 cm_ in fixed fields by accelerator	11
**HHFA024**	Left colectomy with release of the left colic angle, without restoration	10
**GCQX004**	Pathological examination for oncological purposes of cavity excised specimen	10
**GDQX008**	Pathological examination for oncological purposes of laryngectomy specimen	10
**LAFA018**	Bilateral total ethmoidectomy with sphenoidotomy, by endoscopy	10
**FFQX005**	Pathological examination for oncological purposes of splenectomy specimen	10
**JKFA028**	Total hysterectomy with unilateral or bilateral adnexectomy, by laparotomy	10
**JHFA017**	Orchiectomy, via scrotal approach	10
**KCFA008**	Unilateral lobo-isthmectomy of the thyroid gland, by cervicotomy	10
**AAFA002**	Excision of intraparenchymal brain tumor, by craniotomy	9
**HHFA002**	Left colectomy with release of the left colic angle, with restoration	9
**HCFA008**	Partial parotidectomy with dissection and preservation of the facial nerve	9
**GFQX009**	Pathological examination for oncological purposes of pulmonary excision specimen	9
**FAFA015**	Tonsillectomy by dissection, with adenoidectomy	9
**HAFA007**	Partial oral pelvectomy, via intraoral approach	9
**PDQX005**	Pathological examination for oncological purposes of a tumor excision specimen	9
**ZZNL062**	External irradiation session by linear accelerator of equal or greater power	9
**YYYY197**	Irradiation of less than 300 cm_ in fixed fields by accelerator	9
**HHFA008**	Right colectomy with restoration of continuity, by laparoscopy	8
**HHFA030**	Total coloproctectomy without restoration of continuity, by laparotomy	8
**GDQX005**	Pathological examination for oncological purposes of laryngectomy specimen	8

**GDNE003**	Destruction of laryngeal lesion by direct laryngoscopy with laser	8
**HCFA009**	Total parotidectomy with dissection and preservation of the facial nerve	8
**HDFA005**	Total pharyngolaryngectomy	8
**KCNL003**	Internal irradiation of the thyroid gland by administration of iodine 131 at a dose	8
**27 FCFA**	Complete unilateral cervical lymph node dissection, extended to the muscles	8
**LAFA022**	Bilateral total ethmoidectomy, by endoscopy	8
**GHFA004**	Excision of mediastinal tumor, by thoracotomy	7
**ZAQA001**	Exploration of the neck, by cervicotomy	7
**28 FCFA**	Excision of lymph node [node] of the neck for therapeutic purposes, by cervicotomy	7
**GDQX004**	Pathological examination for oncological purposes of laryngeal cordectomy specimen	7
**GCFD001**	Excision of nasopharynx lesion, nasally and/or orally	7
**HAND004**	Destruction of lesion of the mucous membrane of the mouth or oropharynx	7
**HAFA004**	Interrupting pelvimandibulectomy, via cervicofacial approach	7
**JGNL001**	Prostate brachytherapy by permanent insertion of iodine 125	7
**KCQX004**	Pathological examination for oncological purposes of subthyroidectomy specimen	7
**JKND002**	Destruction of lesion of the cervix, vagina, vulva, perineum	7
**JDNE001**	Destruction of bladder lesion, by endoscopy	7
**ZZNL058**	External irradiation session in stereotaxic conditions by machine producing	7
**HKQX005**	Pathological examination for oncological purposes of an excision specimen of the anus canal	6
**HHQX007**	Pathological examination for oncological purposes of appendectomy specimen	6
**HHFA022**	Total colectomy with preservation of the rectum, with ileorectal anastomosis	6
**HJFA002**	Rectosigmoid resection with infraperitoneal colorectal anastomosis	6
**HJFD005**	Resection of the rectal mucosa with anterior hemicircumferential plication	6
**JJFA004**	Salpingoovariectomy [Annexectomy], by laparotomy	6
**HNQX006**	Pathological examination for oncological purposes of a specimen of duodenopancreatectomy	6
**KCQX005**	Pathological examination for oncological purposes of lobectomy specimen	6
**BZQX004**	Pathological examination for oncological purposes of an excerpted organ specimen	6
**FEFF002**	Collection of blood hematopoietic stem cells by cytapheresis	6

**LAFA024**	Unilateral total ethmoidectomy, by endoscopy	6
**LAFA017**	Unilateral total ethmoidectomy with sphenoidotomy, by endoscopy	6
**JAFC006**	Unilateral total nephrectomy, by laparoscopic or retroperitoneoscopy	6
**JAFA023**	Unilateral total nephrectomy, by laparotomy	6
**JAFA009**	Total nephrectomy extended to the renal compartment, by laparotomy or lumbosacral approach	6
**YYYY048**	Irradiation of less than 300 cm_ in fixed fields by accelerator	6
**AFLB003**	Intrathecal injection session of anticancer pharmacological agent	5
**HHQX010**	Pathological examination for oncological purposes of total colectomy specimen	5
**HHFA017**	Left colectomy without release of the left colic angle, with restoration	5
**HHFA010**	Left colectomy without release of the left colic angle, with restoration	5
**HHFA005**	Total colectomy with preservation of the rectum, without restoration of continuity	5
**HHFA031**	Total coloproctectomy with ileoanal anastomosis, by laparotomy	5
**HJFA004**	Rectosigmoid resection with infraperitoneal colorectal anastomosis,	5
**HJFA006**	Rectosigmoid resection by laparotomy, with coloanal anastomosis	5
**GDNE002**	Destruction of laryngeal lesion, by direct laryngoscopy without laser	5
**GDFA005**	Total laryngectomy	5
**HDQX007**	Pathological examination for oncological purposes of a pharyngolaryngectal specimen	5
**HDFA019**	Oropharyngectomy by mandibulotomy	5
**QEQX008**	Pathological examination for oncological purposes of a mastectomy specimen	5
**KDQX005**	Pathological examination for oncological purposes of parathyroidectomy specimen	5
**JKQX005**	Pathological examination for oncological purposes of hysterectomy specimen	5
**JDFA008**	Total cystectomy with transintestinal cutaneous ureterostomy by loop	5
**JHFA008**	Orchidectomy extended to the spermatic cord [Orchiepididymectomy], by inward approach	5
**KEFC002**	Partial or total adrenalectomy, by laparoscopy or retroperitoneoscopy	5
**ZZNL053**	External irradiation session by linear accelerator of equal or greater power	5

**YYYY211**	Irradiation of less than 300 cm_ in fixed fields by accelerator by fraction	5
**YYYY459**	Irradiation of 300 cm_ and more in fixed fields by accelerator per fraction	5
**YYYY109**	Supplement for using custom and focused caches	5
**HHQX011**	Pathological examination for oncological purposes of coloprotectectomy specimen	4
**HJQX004**	Pathological examination for oncological purposes of rectosigmoidectomy	4
**HFFA005**	Total gastrectomy with restoration of continuity, by laparotomy	4
**FAQX004**	Pathological examination for oncological purposes of tonsillectomy specimen	4
**LAFA019**	Bilateral total ethmoidectomy with nasal septoplasty and sphenoidotomy	4
**GDFA008**	Extended supraglottic laryngectomy at the base of the tongue	4
**HCFA011**	Submandibular gland excision	4
**HDFA008**	Lateral oropharyngectomy, by cervicotomy	4
**HDFA009**	Posterior oropharyngectomy, via intraoral approach	4
**HDFA016**	Lateral pharyngectomy, by cervicotomy	4
**JJFC009**	Bilateral oophorectomy, by laparoscopy	4
**HNFA013**	Left pancreatectomy with splenectomy	4
**JGNJ900**	Destruction of prostate lesions by high intensity focused ultrasound,	4
**JHQX005**	Pathological examination for oncological purposes of total orchiectomy specimen	4
**KCNL004**	Internal irradiation of the thyroid gland by administration of iodine 131 at a dose	4
**LAFA015**	Unilateral total ethmoidectomy with nasal septoplasty and sphenoidotomy	4
**HPFC002**	Resection of the greater omentum, by laparoscopy	4
**JHFA006**	Orchiectomy with testicular prosthesis placement, via inguinal approach	4
**LAFA012**	Shaping resection of the middle level of the face, via intraoral approach	4
**YYYY056**	Irradiation in cyclotherapy by accelerator by fraction of 2 Grays	4
**ZANL001**	External intracranial irradiation in stereotaxic conditions with frightening frame	3
**HHFA004**	Total colectomy with preservation of the rectum, with ileorectal anastomosis	3
**HHFA028**	Total coloproctectomy with ileoanal anastomosis, by laparoscopy or laparotitis	3
**HJFC023**	Secondary proctectomy by laparoscopy with ileoanal anastomosis	3

**JFQX004**	Pathological examination for oncological purposes of total pelvectomy specimen	3
**HFQX008**	Pathological examination for oncological purposes of total gastrectomy specimen	3
**FBQX004**	Pathological examination for oncological purposes of a thymus excision specimen	3
**HEQX004**	Pathological examination for oncological purposes of ophagectomy specimen	3
**GDFE007**	Laryngeal cordectomy, by direct laryngoscopy without laser	3
**HEQX008**	Pathological examination for oncological purposes of ophagopharyngeal specimen	3
**HAND005**	Destruction of lesion of the mucous membrane of the mouth or oropharynx	3
**HDFA011**	Oropharyngectomy with interruptive partial mandibulectomy, via cervical approach	3
**GFQX008**	Pathological examination for oncological purposes of pneumonectomy specimen	3
**GFQX005**	Pathological examination for oncological purposes of a lobectomy specimen	3
**GFFA024**	Pneumonectomy, by thoracotomy	3
**JKND003**	Destruction of lesion of the cervix by colposcopy, with laser	3
**JKFC005**	Total hysterectomy, by laparoscopy	3
**JKFC003**	Total hysterectomy with unilateral or bilateral adnexectomy, by fluoroscopy	3
**JKNL002**	Intracavitary uterovaginal brachytherapy at pulsed dose rate, with dosimetry	3
**JDQX005**	Pathological examination for oncological purposes of cystectomy specimen	3
**JDFA025**	Total vesiculo-prostato-cystectomy with transintestinal cutaneous ureterostomy	3
**FAFA006**	Tonsillectomy by dissection, with adenoidectomy and unilateral myringotomy o	3
**LAFA023**	Unilateral anterior ethmoidectomy with nasal septoplasty, by endoscopy	3
**HAFA017**	Interrupting pelvi-glosso-mandibulectomy, via cervicofacial approach	3
**JAFA024**	Partial nephrectomy without dissection of the vascular pedicle, by lombotomy or	3
**KCFA001**	Unilateral lobectomy of the thyroid gland, by cervicotomy	3
**ZZMP012**	Preparation for intracranial irradiation in stereotaxic conditions on the back	3
**ZZNL048**	External irradiation session by linear accelerator of equal or greater power	3
**YYYY151**	Supplement for quality ballistic controls in radiotherapy by gammagraph	3

**HFFA003**	Partial upper gastrectomy [upper polar] with restoration of the	2
**HFFA002**	Partial lower gastrectomy with gastroduodenal anastomosis, by laparotomy	2
**HFFC002**	Lower partial gastrectomy with gastrojejunal anastomosis, by laparoscopic	2
**JJQX004**	Pathological examination for oncological purposes of oophorectomy specimen	2
**JJFC008**	Unilateral oophorectomy, by laparoscopy	2
**HNQX007**	Pathological examination for oncological purposes of pancreatectomy specimen	2
**HNFA005**	Excision of pancreatic tumor, by laparotomy	2
**GFQX010**	Pathological examination for oncological purposes of pulmonary excision specimen	2
**GFFA004**	Pulmonary lobectomy with resection-anastomosis or bronchus re-implantation	2
**GFFA027**	Pulmonary lobectomy with resection of the chest wall, by thoracotomy	2
**GFFA022**	Pulmonary lobectomy with resection of organ and/or neighboring structure,	2
**JKFA025**	Total hysterectomy with anterior or posterior colpoperineorrhaphy	2
**JKFA002**	Total hysterectomy with anterior and posterior colpoperineorrhaphy	2
**JDFA023**	Total vesiculo-prostato-cystectomy with cutaneous ureterostomy, by laparotomy	2
**LAFA016**	Unilateral total ethmoidectomy with nasal septoplasty, by endoscopy	2
**LAFA020**	Bilateral total ethmoidectomy with nasal septoplasty, by endoscopy	2
**GDFA006**	Vertical partial laryngectomy	2
**GDFA016**	Anterior frontal laryngectomy with epiglottoplasty	2
**GDFA013**	Hemilaryngopharyngectomy	2
**GDFA018**	Total laryngectomy extended to the base of the tongue	2
**GGLB001**	Intrapleural injection session of anticancer pharmacological agent	2
**HNQX004**	Pathological examination for oncological purposes of papil specimen	2
**HAND002**	Destruction of lesion of the mucous membrane of the mouth or oropharynx	2
**HAND001**	Destruction of lesion of the mucous membrane of the mouth or oropharynx from 2 cm to 4	2
**HCFA001**	Submandibular gland excision enlarged	2
**HCFA004**	Partial parotidectomy without facial nerve dissection	2
**HDNE001**	Destruction of hypopharyngeal lesion with laser, by endoscopy	2

**HJND001**	Destruction of rectal lesion, anally	2
**HJFA012**	Secondary proctectomy by laparotomy with trans ileoanal anastomosis	2
**HPLB003**	Intraperitoneal administration of anticancer pharmacological agent with hyper	2
**JDQX004**	Pathological examination for oncological purposes of total cystectomy specimen	2
**JHQX008**	Pathological examination for oncological purposes of partial amputation specimen	2
**JAFA032**	Total nephroureterectomy, by direct approach	2
**JAFA016**	Binephrectomy, by laparotomy	2
**JAFA029**	Total nephrectomy extended to the renal compartment with adrenalectomy, by laparotomy	2
**JDFA001**	Total cystectomy with cutaneous ureterostomy, by laparotomy	2
**JKFA020**	Total colpohysterectomy extended to parameters, by laparoscopy and by vaginal approach	2
**LBFA005**	Total maxillectomy without reconstruction	2
**LHFH001**	Vertebral bone tumorectomy, transcutaneously with CT guidance	2
**PAFH001**	Osteoarticular tumorectomy of a limb, transcutaneously with sc guidance	2
**ZZNL061**	External irradiation session using a machine with a power of less than 5 megavolts	2
**ZZNL045**	External proton irradiation session	2
**YYYY050**	Irradiation of less than 300 cm_ in fixed fields by accelerator	2
**YYYY244**	Irradiation of less than 300 cm_ in fixed fields by accelerator	2
**YYYY304**	Irradiation of 300 cm_ and more in fixed fields by accelerator	2
**YYYY337**	Irradiation of 300 cm_ and more in fixed fields by accelerator	2
**YYYY492**	Irradiation of 300 cm_ and more in fixed fields by accelerator	2
**YYYY055**	Irradiation in cyclotherapy by accelerator in fractions of 20 centiGrays	2
**HJFA001**	Rectocolic resection with retrorectal colic lowering by laparotomy	1
**HELE001**	Installation of intra-esophageal guide for brachytherapy	1
**LAFA014**	Mastoidectomy or total atticomastoidectomy	1
**JJQX005**	Pathological examination for carcinological purposes of several ovariects	1
**JJFA003**	Unilateral oophorectomy, by laparotomy	1
**JJFA005**	Bilateral oophorectomy, by laparotomy	1
**HNFA008**	Left pancreatectomy with preservation of the spleen, by laparotomy	1
**HNFA004**	Total duodenopancreatectomy with splenectomy	1
**GENE006**	Destruction of lesion of the tracheobronchial tree by cryotherapy or thermocoagulation	1

**GFFA008**	Upper lung lobectomy with side resection and plexus release	1
**GFFA019**	Excision of remaining lung lobe, by thoracotomy	1
**GFFA007**	Pneumonectomy with resection of organ and/or neighboring structure, by thoracotomy	1
**JGND002**	Prostate cryotherapy	1
**FENL001**	Internal irradiation for polycythemia, by intravenous injection of phosphorus	1
**ZZMP001**	Preparing for total body irradiation	1
**JKFC002**	Subtotal hysterectomy, by laparoscopy	1
**JKFA024**	Subtotal hysterectomy, by laparotomy	1
**JKFA005**	Total hysterectomy with unilateral or bilateral annexectomy, via vaginal approach	1
**JLNL002**	High dose rate intracavitary vaginal brachytherapy with two-dimensional dosimetry	1
**JDFA021**	Total vesiculo-prostato-cystectomy with replacement enterocystoplasty	1
**BKQX004**	Pathological examination for oncological purposes of exenteration part of the orbit	1
**DZQX005**	Pathological examination for oncological purposes of a tumor excision specimen	1
**FAFA010**	Tonsillectomy by dissection, with adenoidectomy and bilateral aerator placement	1
**GCQX005**	Pathological examination for oncological purposes of rhinoplasty specimen	1
**GBFA008**	Bilateral anterior ethmoidectomy with nasal septoplasty, by endoscopy	1
**GDFA010**	Arytenoidectomy or arytenoidopexy, by thyrotomy	1
**GDFA014**	Supraglottic laryngectomy	1
**GDFA011**	Supracricoid laryngectomy with crico-hyoido-epiglottopexy	1
**GHFA003**	Excision of mediastinal tumor with resection of organ and/or voila structure	1
**HAFA001**	Basic partial glossectomy, by cervicotomy	1
**HAFA003**	Total mobile tongue glossectomy, via cervicofacial approach	1
**HAFA027**	Mobile tongue pelviglossectomy, via cervicofacial approach	1
**HAFA033**	Non-interrupting pelvimandibulectomy, via intraoral approach	1
**HAFA024**	Non-interrupting pelvimandibulectomy, via cervicofacial approach	1
**HAFA002**	Non-interrupting pelvi-glosso-mandibulectomy, via intraoral approach	1
**HAFA026**	Non-interrupting pelvi-glosso-mandibulectomy, via cervicofacial approach	1
**HCFA005**	Total parotidectomy with excision of the parapharyngeal extension	1

**HDNE002**	Destruction of hypopharyngeal lesion without laser, by endoscopy	1
**HDFA004**	Oropharyngectomy with non-interrupting partial mandibulectomy	1
**HDFA010**	Total circular pharyngolaryngectomy	1
**HHFA029**	Total coloproctectomy without restoration of continuity, by laparoscopic or laparotomy	1
**HLNN900**	Destruction of hepatic tumor by radiofrequency, transcutaneously	1
**JEQX005**	Pathological examination for oncological purposes of partial ureterectomy	1
**JHQX007**	Pathological examination for oncological purposes of total amputation specimen	1
**JKQX006**	Pathological examination for oncological purposes of trachelectomy specimen	1
**JMQX005**	Pathological examination for oncological purposes of specimen of vulvectomy	1
**JAFA030**	Partial nephrectomy without dissection of the vascular pedicle, by laparotomy	1
**JAFA019**	Partial nephrectomy with dissection of the vascular pedicle, by laparotomy	1
**JAFA002**	Total nephrectomy, by lombotomy	1
**JAFC001**	Total nephroureterectomy, by laparoscopic route or retroperitoneoscopy	1
**JAFC019**	Total nephrectomy extended to the renal compartment, by laparoscopy or retroperitoneoscopy	1
**JDFA011**	Partial cystectomy, by laparotomy	1
**JDFA005**	Total cystectomy, by laparotomy	1
**JDFA016**	Total cystectomy with replacement enterocystoplasty	1
**JKND004**	Destruction of lesion of the cervix by colposcopy, without laser	1
**JKFA027**	Total colpohysterectomy extended to parameters, by laparotomy	1
**JMFA004**	Total vulvectomy with bilateral inguinal and iliac lymph node dissection	1
**JFFA003**	Anterior pelvectomy with transintestinal cutaneous ureterostomy by loop	1
**KCFA007**	Total thyroidectomy, by cervicothoracotomy	1
**KCMA001**	Secondary totalization of thyroidectomy, by cervicotomy	1
**LBFA006**	Upper maxillectomy without reconstruction	1
**LBFA018**	Shaping resection of the mandible, via intraoral approach	1
**PZQX004**	Pathological examination for oncological purposes of partial amputation specimen	1
**QEQX005**	Pathological examination for oncological purposes of several mastectomy specimens	1

**QEQX007**	Pathological examination for oncological purposes of several mastectomy specimens	1
**QZFA033**	Excision of superficial lesion of the skin by excision of a skin area	1
**ZZMK019**	Resumption of preparation for external irradiation without dosimetry, with simulation	1
**ZZNL064**	External irradiation session by linear accelerator of equal or greater power	1
**ZZNL055**	External irradiation in stereotaxic conditions by dedicated machine	1
**ZZNL018**	Deep interstitial brachytherapy with pulsed dose rate, with tri dosimetry	1
**ZZNL003**	Interstitial brachytherapy with pulsed dose rate after vector placement	1
**ZZMP004**	Manufacturing of a personalized applicator for brachytherapy	1
**ZZMP003**	Manufacturing of personalized lead protection for brachytherapy	1
**YYYY307**	Irradiation of 300 cm_ and more in fixed fields by accelerator per fraction of 20 centigrays for beams ranging from 5 to 7.9 MeV.	1
**YYYY347**	Irradiation of 300 cm_ and more in fixed fields by accelerator per fraction of 1.4 grays for beams ranging from 0.5 to 4.9 MeV.	1
**YYYY387**	Irradiation of 300 cm_ and more in fixed fields by accelerator per fraction of 2 grays for beams ranging from 5 to 7.9 MeV.	1
**YYYY481**	Irradiation of 300 cm_ and more in fixed fields by accelerator per fraction of 12 centigrays for beams ranging from 17 to 24.9 MeV.	1
**YYYY316**	Irradiation of 300 cm_ and more in fixed fields by accelerator per fraction of 1.2 grays for beams ranging from 17 to 24.9 MeV.	1
**YYYY360**	Irradiation of 300 cm_ or more in fixed fields by accelerator per fraction of 12 grays for beams more than 25 MeV.	1
**YYYY451**	Irradiation of 300 cm_ or more in fixed fields by accelerator per fraction of 0.9 grays for beams more than 25 MeV.	1
**YYYY267**	Irradiation in cyclotherapy by accelerator in fractions of 12 centiGrays	1

Table S4: Summary of protocol components of the emulated target trials.

Protocol Component	Description of Emulation
**Research questions**	Is EVR treatment after LT associated with a lower occurrence of DNM?<br>Is TAC discontinuation in an EVR initiator involved in the occurrence of DNM?
**Outcomes**	DNM occurrence<br>Overall survival
**Eligibility criteria**	Discharged after LT<br>Older than 18 years<br>First liver transplantation between 01/01/2009 and 01/01/2020
**Exclusions**	No exclusion criteria
**Treatment strategies**	ETT1: EVR initiation or not<br>ETT2: EVR discontinuation or not in EVR initiator<br>ETT3: TAC discontinuation or not in EVR initiator
**Treatment assignment**	Emulated randomization
**Follow up time**	ETT1: 10 years after LT<br><br>ETT2 or ETT3: 10 years after initiation
**Grace periods**	ETT1: 3 years after LT<br><br>ETT2 or ETT3: 3 years after EVR initiation
**Estimand**	Hazard ratio
**Analysis plan**	Emulation of target trial: Analysis conducted on the cloned data set assuming random treatment assignment and with a censoring indicator when there is a protocol deviation within the grace period; selection bias accounted for by using inverse probability of artificial censoring weights.<br><br>Type of primary analysis and statistical model: HR approach
**Adjustment variables**	Based on Cox regression analysis for DNM occurrence: age at transplant, sex, smoking status, alcohol-related liver disease, diabetes before transplant, HCC as transplant indication, recurrence of HCC post-transplant, pre-transplant creatinine, HCV, income level, chronic kidney disease, local unemployment rate, pre-transplant ascites, hepatic encephalopathy, and transplant center

**Sensitivity analyses**	Site-specific analysis<br><br>Role of grace period and lag-time

Table S5: Multivariate Generalized Additive Model (GAM).

To validate the associations suggested by the IPCW-RF, a parametric generalized additive model (GAM) was fitted. This approach estimates adjusted effects for each covariate on the hazard of death or retransplantation. This table reports the estimated coefficients (Estimate), standard errors (SE), z-values and corresponding p-values. Variables with p <0.05 were considered to be statistically significant.

Variable	OR (95% CI)	z-value	p-value
**Demographic**			
**Female sex**	0.87 (0.76–0.99)	−2.09	0.037
**Socioeconomic factors**			
**Area-level unemployment**	1.03 (1.01–1.05)	2.77	0.006
**Area-level income**	1.00 (1.00–1.00)	−0.10	0.919
**Pre-transplant comorbidities**			
**Tobacco use**	1.10 (0.99–1.24)	1.72	0.085
**Alcohol-related liver disease**	1.10 (0.96–1.26)	1.34	0.180
**Cardiovascular disease**	1.11 (0.98–1.25)	1.65	0.098
**Diabetes mellitus**	0.93 (0.83–1.03)	−1.36	0.175
**Arterial hypertension**	1.00 (0.88–1.14)	0.02	0.983
**Stroke**	1.33 (1.01–1.76)	2.00	0.046
**Sleep apnea**	1.00 (0.81–1.24)	0.03	0.977
**Psychiatric disorders**	0.93 (0.80–1.09)	−0.90	0.368
**HIV infection**	0.65 (0.44–0.96)	−2.15	0.032
**Pre-LT renal failure**	0.57 (0.37–0.89)	−2.45	0.014
**Liver disease severity before transplantation**			
**Ascites**	0.96 (0.85–1.08)	−0.68	0.498
**Hepatic encephalopathy**	0.90 (0.80–1.02)	−1.59	0.113
**Sepsis before LT**	0.97 (0.86–1.10)	−0.45	0.656
**TIPS procedure**	1.18 (0.96–1.46)	1.57	0.116
**Acute hemodialysis before LT**	1.04 (0.93–1.16)	0.67	0.504
**Underlying liver disease**			
**HCC as primary indication**	1.47 (1.30–1.66)	6.18	<0.001
**Hepatitis C infection**	0.88 (0.78–1.00)	−1.95	0.052
**Hepatitis B infection**	0.85 (0.72–1.01)	−1.86	0.063
**Autoimmune liver disease**	1.10 (0.82–1.47)	0.62	0.534
**Secondary biliary cirrhosis**	0.77 (0.54–1.09)	−1.47	0.142
**Cancer history**			

**Extrahepatic cancer before LT**	1.16 (0.72–1.86)	0.61	0.539
**Cancer before transplantation**	1.30 (0.72–2.34)	0.88	0.377
**Post-transplant events**			
**HCC recurrence**	4.25 (3.38–5.33)	12.47	<0.001
**Renal dysfunction after LT**	1.34 (1.20–1.49)	5.28	<0.001

Table S6: Covariate balance before and after inverse probability of censoring weighting.

**Covariate**	**No-EVR strategy (Unweighted)**	**EVR strategy (Unweighted)**	**No-EVR strategy (IPCW-weighted)**	**EVR strategy (IPCW-weighted)**	**Absolute SMD**
**Baseline covariates**					
**Age at liver transplantation (years)**	53.01	53.63	53.25	53.63	0.033
**Extrahepatic cancer count before LT**	0.052	0.056	0.052	0.056	0.013
**Area-level income**	17587	17572	17586	17572	0.002
**Area-level unemployment**	8.094	8.112	8.104	8.112	0.003
**Sex**	0.233	0.223	0.229	0.223	0.005
**Tobacco use**	0.318	0.318	0.318	0.318	<0.001
**Alcohol-related liver disease**	0.663	0.680	0.669	0.680	0.011
**Cardiovascular disease**	0.237	0.246	0.239	0.246	0.007
**Diabetes mellitus**	0.331	0.340	0.335	0.340	0.006
**Arterial hypertension**	0.699	0.707	0.701	0.707	0.006
**Stroke**	0.025	0.028	0.026	0.028	0.002
**Sleep apnea**	0.055	0.056	0.055	0.056	0.001
**Psychiatric disorders**	0.102	0.099	0.099	0.099	<0.001
**Inflammatory bowel disease**	0.039	0.036	0.038	0.036	0.002
**HIV infection**	0.027	0.024	0.026	0.024	0.001
**Pre-LT renal failure**	0.016	0.016	0.016	0.016	<0.001
**Cancer before transplantation**	0.045	0.048	0.045	0.048	0.003

**Ascites**	0.572	0.572	0.573	0.572	<0.001
**Hepatic encephalopathy**	0.277	0.274	0.276	0.274	0.002
**Sepsis before LT**	0.232	0.228	0.229	0.228	0.001
**TIPS procedure**	0.054	0.058	0.055	0.058	0.002
**HCC as primary indication**	0.418	0.443	0.425	0.443	0.018
**Hepatitis C infection**	0.286	0.283	0.287	0.283	0.004
**Hepatitis B infection**	0.118	0.115	0.116	0.115	0.001
**Hepatitis D coinfection**	0.028	0.027	0.028	0.027	0.001
**Autoimmune liver disease**	0.044	0.041	0.043	0.041	0.001
**Secondary biliary cirrhosis**	0.037	0.034	0.036	0.034	0.001
**Time-varying covariates**					
**HCC recurrence**	0.023	0.031	0.024	0.031	0.007
**Renal dysfunction**	0.211	0.232	0.215	0.232	0.017

Table S7: Effects of treatments in trials ETT-1 to ETT-3: risk difference (RD), hazard ratio (HR), number needed to treat/harm (NNT/NNH), and E-value

Trial	RD median (IQR)	HR median (95% CI)	NNT / NNH	E-value (point ; lower)
**ETT-1**	−0.0646 (−0.0963; −0.0428)	0.84 (0.79–0.88)	NNT 16 (10–23)	1.67
**ETT-2**	0.0505 (0.0341; 0.0765)	1.34 (1.21–1.45)	NNH 20 (13–29)	1.84
**ETT-3**	0.1258 (0.1159; 0.1388)	1.03 (0.83–1.21)	NNH 8 (7–9)	1.16

*RD = Risk Difference; HR = Hazard Ratio; NNT = Number Needed to Treat; NNH = Number Needed to Harm; IQR = Interquartile Range; CI = Confidence Interval.

Table S8: Sensitivity analyses of ETT-2 using alternative dispensing-gap definitions (30, 60, and 180 days).

Gap definition	Outcome	HR, median (Q1–Q3)	RD, median (Q1–Q3)	NNT/NNH	E-value
**30 days**	DNM occurrence	1.40 (1.37–1.43)	0.06 (0.05–0.06)	17.28 (NNH)	2.15
**30 days**	Overall survival	1.41 (1.36–1.46)	−0.02 (−0.06 to 0.01)	43.86 (NNT)	2.17
**60 days**	DNM occurrence	1.43 (1.29–1.55)	0.08 (0.05–0.10)	12.90 (NNH)	2.23
**60 days**	Overall survival	1.88 (1.81–2.13)	0.19 (0.09–0.33)	5.19 (NNH)	3.18
**180 days**	DNM occurrence	1.60 (1.58–1.63)	0.06 (0.05–0.09)	16.10 (NNH)	2.58
**180 days**	Overall survival	2.03 (2.00–2.25)	0.03 (−0.10 to 0.07)	34.21 (NNH)	3.49

Abbreviations: HR, hazard ratio; RD, risk difference; NNT, number needed to treat; NNH, number needed to harm. Gap definitions refer to the maximum allowed interval between dispensings used to define treatment discontinuation in ETT-2.
