# Supplementary material for: Everolimus and de novo malignancies after liver transplantation: A nationwide emulated trial using real-world data
Source: JHEP Rep. 2026 Aug 7;8(9):101934. doi: 10.1016/j.jhepr.2026.101934 (PMC13476556; doi:10.1016/j.jhepr.2026.101934)
Supplement: Supplementary file 4 [file mmc2.docx]

**JHEP Reports**

**CTAT methods**

Tables for a “Complete, Transparent, Accurate and Timely account” (CTAT) are now mandatory for all revised submissions. The aim is to enhance the reproducibility of methods.

- Only include the parts relevant to your study
- Refer to the CTAT in the main text as ‘Supplementary CTAT Table’
- Do not add subheadings
- Add as many rows as needed to include all information
- Only include one item per row

**If the CTAT form is not relevant to your study, please outline the reasons why:**

Not applicable. This study is an emulated target trial using observational data from a nationwide administrative database (SNDS) and does not involve experimental laboratory reagents or biological assays.

1.1 **Antibodies**

| **Name** | **Citation** | **Supplier** | **Cat no.** | **Clone no.** |
| --- | --- | --- | --- | --- |
| **Not applicable.** |  |  |  |  |

1.2 **Cell lines**

| **Name** | **Citation** | **Supplier** | **Cat no.** | **Passage no.** | **Authentication test method** |
| --- | --- | --- | --- | --- | --- |
| **Not applicable.** |  |  |  |  |  |

1.3 **Organisms**

| **Name** | **Citation** | **Supplier** | **Strain** | **Sex** | **Age** | **Overall n number** |
| --- | --- | --- | --- | --- | --- | --- |
| **Not applicable.** |  |  |  |  |  |  |

1.4 **Sequence based reagents**

| **Name** | **Sequence** | **Supplier** |
| --- | --- | --- |
| **Not applicable.** |  |  |

1.5 **Biological samples**

| **Description** | **Source** | **Identifier** |
| --- | --- | --- |
| **Not applicable.** |  |  |

1.6 **Deposited data**

| **Name of repository** | **Identifier** | **Link** |
| --- | --- | --- |
| **French National Health Data System (SNDS)** | **Access restricted under CNIL authorization N°919274** | **Not publicly available; access via French health authorities** |

1.7 **Software**

| **Software name** | **Manufacturer** | **Version** |
| --- | --- | --- |
| **R** | **R Foundation for Statistical Computing** | **2023.12.0** |

1.8 **Other (*e.g*. drugs, proteins, vectors etc.)**

Everolimus (EVR) exposure defined using ATC-linked dispensing data

Tacrolimus (TAC) exposure defined using ATC-linked dispensing data

Mycophenolate mofetil (MMF) use from dispensing data

Liver transplantation identified using CCMP codes HLEA001 and HLEA002

Cancer outcomes defined using ICD-10 diagnosis codes combined with CCMP procedures

Socioeconomic indicators derived from French département-level data

Covariates including comorbidities defined using ICD-10 algorithms

Time-varying confounders: tumor recurrence and renal dysfunction

Cloning–censoring–weighting (CCW) framework for causal inference

Inverse probability of censoring weighting (IPCW)

1.9 **Please provide the details of the corresponding methods author for the manuscript:**

**Ilias KOUNIS, AP-HP Hôpital Paul-Brousse, Centre Hépato-Biliaire, 12 Avenue Paul Vaillant Couturier, 94800, Villejuif, France, ilias.kounis@aphp.fr**

**2.0	Please confirm for randomised controlled trials all versions of the clinical protocol are included in the submission. These will be published online as supplementary information.**

**Not applicable. This study is an emulated target trial based on observational data and does not include a randomized clinical protocol.**
